# Supplementary material for: Building bridges through dynamic coupling for organic phosphorescence
Source: Nat Commun. 2025 Jul 9;16:6339. doi: 10.1038/s41467-025-61714-0 (PMC12241521; doi:10.1038/s41467-025-61714-0)
Supplement: Supplementary file 1 — Supplementary Information [file 41467_2025_61714_MOESM1_ESM.pdf]

Supplementary Information

Building Bridges through Dynamic Coupling for Organic Phosphorescence

Xin Li[1,#], Wenlang Li[1,#], Ziqi Deng[2], Jingtian Wang[3], Shan He[1], Xinwen Ou[1], David Lee Phillips[2], Guanjun Xiao[3,*], Bo Zou[3], Ryan T. K. Kwok[1], Jianwei Sun[1], Jacky W. Y. Lam[1,*], Zhihong Guo[1,*], Ben Zhong Tang[1,4,*]

[1] Department of Chemistry and the Hong Kong Branch of Chinese National Engineering Research Center for Tissue Restoration and Reconstruction, The Hong Kong University of Science and Technology, Clear Water Bay, Kowloon, Hong Kong, 999077, China

[2] Department of Chemistry, The University of Hong Kong, Pokfulam, Hong Kong, 999077, China

[3] State Key Laboratory of Superhard Materials, College of Physics, Jilin University, Changchun, 130012, China

[4] School of Science and Engineering, Shenzhen Institute of Aggregate Science and Technology, The Chinese University of Hong Kong, Shenzhen (CUHK-Shenzhen), Shenzhen, 518172, China

[#] These authors contributed equally

* Correspondence: xguanjun@jlu.edu.cn (G.X.); chjacky@ust.hk (J.W.Y.L.); chguo@ust.hk (Z.G.); tangbenz@cuhk.edu.cn (B.Z.T.)

Contents

I. Theoretical calculation methods……………………………………………………………..2

II. Synthesis and characterization of molecules……………………………………………………3

III. Additional information for photophysical mechanism study…………...……………………22

IV. Modulation of coupling strength through pressurization…………………...……………...…34

V. Universal applicability…………………………………..…………………………………43

VI. Dynamic coupling in PY−CzBP/BPCzA system………………….………………………63

VII. Quantitative analysis of dynamic coupling…………………..…………………………80

VIII. Applications……………………………………………………………………………83

IX. Supplementary tables………..…………………………………………..………………..91

X. References…………………….………………………………………………..……….96

I. Theoretical calculation methods

The geometries of all guest molecules and host/guest dimers were optimized at the B3LYP level of theory in the gas phase with 6-31G(d) basis set for all atoms. Analytical frequency calculations were also performed at the same level of theory to confirm the local minimum point of the optimized structures. The excited-state geometries were optimized using the time-dependent density functional theory (TD-DFT) method at the same level of theory. The geometries of all host molecules in the crystalline phase were extracted from single-crystal structures and optimized based on an ONIOM model with the combined quantum mechanics and molecular mechanics methods. The central molecule was treated as the QM part and optimized at the (TD)B3LYP/6-31G(d) level, while the surrounding molecules were frozen to act as the MM part with the universal force field. All the above calculations were carried out using Gaussian 16 program (Revision A.03)[1]. The spin-orbit coupling (SOC) between singlets and triplets were evaluated by using ORCA software (version 4.2.1)[2] based on the TD-DFT results. The natural transition orbital (NTO) analysis was extracted based on TD-DFT results with the aid of Multiwfn package[3]. Noncovalent interactions (NCI) of intermolecular interactions analyses based on dimer structures were carried out by using Multiwfn software with independent gradient model (IGM)[4]. The host/guest dimer structures, NTOs, and molecular orbitals were visualized using Mercury software (version 2021.3.0)[5], VMD program (version 1.9.3)[6] and GaussView 6[7]. The center-to-center distances between aromatic ring planes in host/guest dimers were measured through Mercury and the overlaps were measured using the software ImageJ[8].

II. Synthesis and characterization of molecules

Synthesis

9-hexyl-carbazole (CzA)

Synthesis of CzA was similar to previous report[9]. Sodium hydride (1.2 g, 48.0 mmol) was added into a solution of carbazole (2.7 g, 16.0 mmol) in THF (120 mL). After stirring for 15 min under a nitrogen atmosphere, 1-bromohexane (7.9 g, 48.0 mmol) was added into the solution. The mixture was stirred at 80°C for 12 h. After cooling down to room temperature, the crude product was purified by silica gel column chromatography with *n*-hexane as eluent and then recrystallized from DCM/ethanol twice. Colorless crystals of CzA were obtained in 50% yield (2.0 g). ^{1}H NMR (400 MHz, $CDCl_3$) δ = 8.11 (d, *J* = 7.7 Hz, 2H), 7.52–7.44 (m, 2H), 7.42 (d, *J* = 8.1 Hz, 2H), 7.30–7.18 (m, 2H), 4.30 (t, *J* = 7.3 Hz, 2H), 1.95–1.80 (m, 2H), 1.45–1.26 (m, 6H), 0.87 (dd, *J* = 9.2 Hz, 4.9, 3H). ^{13}C NMR (101 MHz, $CDCl_3$) δ = 140.43, 125.56, 122.81, 120.35, 118.68, 108.66, 77.36, 77.04, 76.72, 43.10, 31.61, 28.96, 27.01, 22.57, 14.04. HRMS (m/z): $[M]^+$ calcd. for $C_{18}H_{21}N$, 251.1674; found, 251.1679.

3,6-dibromo-9-hexyl-carbazole (BrCzA)

N-Bromosuccinimide (NBS) (0.7 g, 4.0 mmol) was added into a solution of CzA (0.5 g, 2.0 mmol) in DCM (20 mL) at 0°C and then stirred at 20°C for 8 h. The reaction bottle was wrapped in tinfoil to avoid light. After that, the crude product was purified by column chromatography with DCM/*n*-hexane (v/v=1:10) as eluent and then recrystallized from DCM/ethanol twice. Colorless crystals of BrCzA were obtained in 98% yield (0.8 g).^{1}H NMR (400 MHz, $CDCl_3$) δ = 8.16 (d, *J* = 1.9 Hz, 2H), 7.58 (dd, *J* = 8.7, 1.9 Hz, 2H), 7.29 (d, *J* = 7.8 Hz, 2H), 4.26 (t, *J* = 7.2 Hz, 2H), 1.91–1.78 (m, 2H), 1.39–1.24 (m, 6H), 0.88 (t, *J* = 7.1 Hz, 3H). ^{13}C NMR (101 MHz, $CDCl_3$) δ = 139.32, 129.01, 123.45, 123.27, 111.93, 110.40, 43.37, 31.51, 28.83, 26.89, 22.51, 13.99. HRMS (m/z): $[M]^+$ calcd. for $C_{18}H_{19}Br_2N$, 408.9864; found, 408.9882.

4-(carbazol-9-yl)-benzophenone (CzBP)

Carbazole (0.2 g, 1.2 mmol), potassium *tert*-butoxide (0.2 g, 2.0 mmol) were dissolved in dry DMF (20 mL) and stirred for 15 min under a nitrogen atmosphere. 4-Fluorobenzophenone (0.2 g, 1.0 mmol) was added into the mixture and the reaction was heated to 70°C and stirred for 6 h. After cooling down to room temperature, the crude product was purified by column chromatography with DCM/*n*-hexane (v/v=1:5) as eluent and then recrystallized from DCM/ethanol twice. Colorless crystals of CzBP were obtained in 57% yield (0.2 g). ^{1}H NMR (400 MHz, $CDCl_3$) δ = 8.16 (d, *J* = 7.7 Hz, 2H), 8.12–8.04 (m, 2H), 7.96–7.87 (m, 2H), 7.79–7.71 (m, 2H), 7.65 (dd, *J* = 10.5, 4.3 Hz, 1H), 7.60–7.50 (m, 4H), 7.49–7.41 (m, 2H), 7.37–7.30 (m, 2H). ^{13}C NMR (101 MHz, $CDCl_3$) δ = 195.67, 141.67, 140.27, 137.45, 136.01, 132.67, 131.91, 130.07, 128.48, 126.31, 126.23, 123.84, 120.61, 120.49, 109.81, 99.99. HRMS (m/z): $[M+Na]^+$ calcd. for $C_{25}H_{17}NONa$, 370.1208; found, 380.1206.

3,6-dibenzoyl-9-hexyl-carbazole (BPCzA)

Synthesis of BPCzA was similar to previous report[10]. Benzoyl chloride (1.0 mL, 8.0 mmol) was added into a solution of anhydrous aluminum chloride (2.7 g, 20.0 mmol) in dry DCM (10 mL) at 0°C and then stirred for 30 min. A solution of CzA (0.5 g, 2.0 mmol) in DCM (10 mL) was added dropwise into the mixture for 15 min, followed by stirring for 30 min at 0°C. The reaction mixture was then heated to 40°C and stirred for 6 h. After cooling down to room temperature, it was then quenched by adding dilute HCl at 0°C. The mixture was extracted with DCM (3 × 100 mL), followed by washing with deionized water (3 × 100 mL). Subsequently, it was dried with anhydrous Na_2SO_4. After evaporation of the solvent, the crude product was purified by column chromatography with DCM/*n*-hexane (v/v=1:1) as eluent and then recrystallized from DCM/ethanol twice. Colorless crystals of BPCzA were obtained in

87% yield (0.8 g). ^{1}H NMR (400 MHz, $CDCl_3$) δ = 8.59 (d, *J* = 1.3 Hz, 2H), 8.08 (dd, *J* = 8.6, 1.7 Hz, 2H), 7.84 (dd, *J* = 5.2, 3.3 Hz, 4H), 7.65–7.58 (m, 2H), 7.52 (dd, *J* = 8.0, 5.7 Hz, 6H), 4.40 (t, *J* = 7.3 Hz, 2H), 2.05–1.85 (m, 2H), 1.50–1.23 (m, 6H), 0.88 (t, *J* = 7.1 Hz, 3H). ^{13}C NMR (101 MHz, $CDCl_3$) δ = 196.49, 143.72, 138.64, 131.96, 129.94, 129.59, 129.06, 128.32, 124.16, 122.70, 108.95, 43.70, 31.51, 28.96, 26.91, 22.53, 14.00. HRMS (m/z): $[M+Na]^+$ calcd. for $C_{32}H_{29}NO_2Na$, 482.2096; found, 482.2094.

2-nitro-1,1'-biphenyl (M)

The synthesis of 2-nitro-1,1'-biphenyl was similar to the previous report[11]. 1-Bromo-2-nitrobenzene (5.0 g, 25.0 mmol) and phenylboronic acid (4.2 g, 35.0 mmol) were dissolved in THF (50 mL) and stirred for 15 min under a nitrogen atmosphere. A 2.0 M aqueous K_2CO_3 solution (30.0 mL) and $Pd(PPh_3)_4$ catalyst (0.05 g) was added and stirred at 80°C for 6 h. After cooling down to room temperature, the crude product was purified by column chromatography with DCM/*n*-hexane (v/v=1:10) as eluent. Compound 2-nitro-1,1'-biphenyl was obtained as a white solid in 70% yield (3.5 g). ^{1}H NMR (400 MHz, $CDCl_3$) δ = 7.89 (d, *J* = 8.1 Hz, 1H), 7.61 (td, *J*=7.6, 1.0 Hz, 1H), 7.53–7.44 (m, 5H), 7.44–7.36 (m, 2H). ^{13}C NMR (101 MHz, $CDCl_3$) δ = 149.41, 137.61, 136.24, 132.60, 132.10, 128.89, 128.47, 128.40, 128.08, 124.18. HRMS (m/z): $[M]^+$ calcd. for $C_{12}H_9NO_2$, 199.0633; found, 199.0636.

9*H*-carbazole (Cz-Lab)

The synthesis of Cz-lab was similar to the previous report[11]. Triphenylphosphine (19.7 g, 75.0 mmol) was added to a solution of 2-nitro-1,1'-biphenyl (5.0 g, 25.0 mmol) in 1,2-dichlorobenzene, and stirred for 15 mins under a nitrogen atmosphere. The reaction mixture was kept at 180°C for 24 h. After cooling down to room temperature, the crude product was purified by column chromatography with DCM/*n*-hexane (v/v=1:5) as eluent and recrystallized from DCM/ethanol twice. Colorless crystals of Cz-Lab were obtained in 60% yield (2.5 g). ^{1}H NMR (400 MHz, $CDCl_3$) δ = 8.09 (d, *J* = 7.8 Hz, 2H), 8.06 (s, 1H), 7.49–7.40 (m, 4H), 7.30–7.21 (m, 2H). ^{13}C NMR (101 MHz, $CDCl_3$) δ = 139.47, 125.85, 123.35, 120.34, 119.45, 110.58. HRMS (m/z): $[M]^+$ calcd. for $C_{12}H_9N$, 167.0735; found, 167.0732.

Cultivation of single crystals

The single crystals of BrCzA, CzBP and BPCzA suitable for the SXRD analysis were grown by solvent diffusion with DCM/ethanol at room temperature for 5 days. The X-ray crystallographic coordinates for single-crystal structures reported in this study have been deposited at the Cambridge Crystallographic Data Centre (CCDC) under deposition numbers CCDC 2368574 (BrCzA), CCDC 2368575 (CzBP), and CCDC 2368576 (BPCzA), respectively. Their crystallographic data were summarized in Supplementary Tables 1−3. The single-crystal structure of CzA was obtained from a previous paper[9], CCDC 1833958 (CzA).

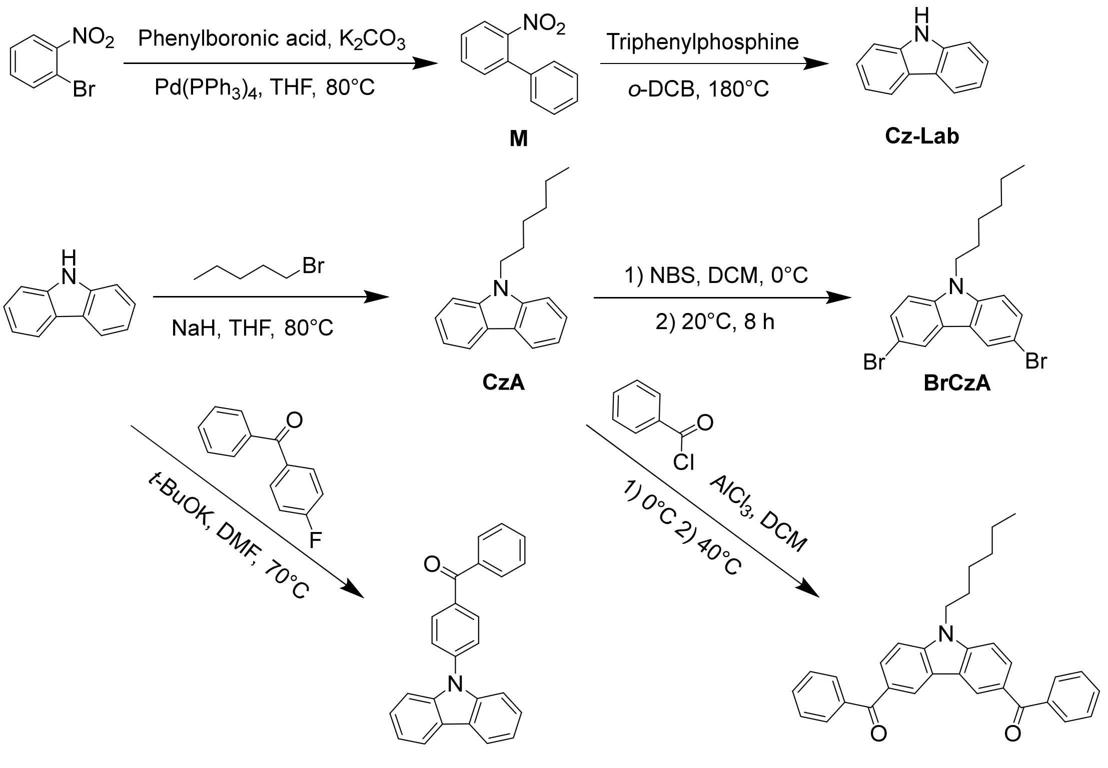

Supplementary Figure 1 | Synthetic routes of host molecules.

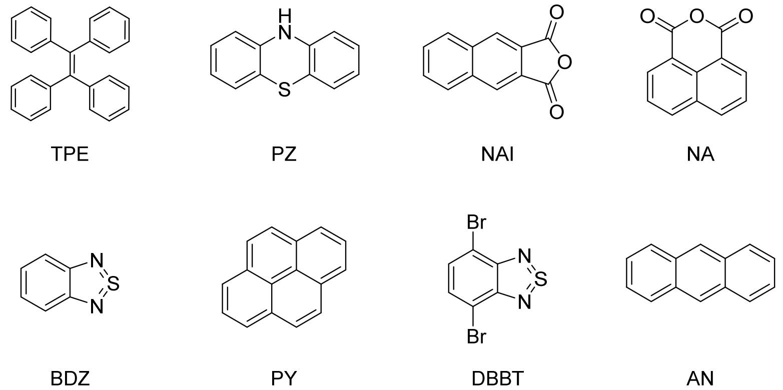

Supplementary Figure 2 | Chemical structures of guest molecules used in this work. All guest molecules were purified through column chromatography and recrystallization twice before use.

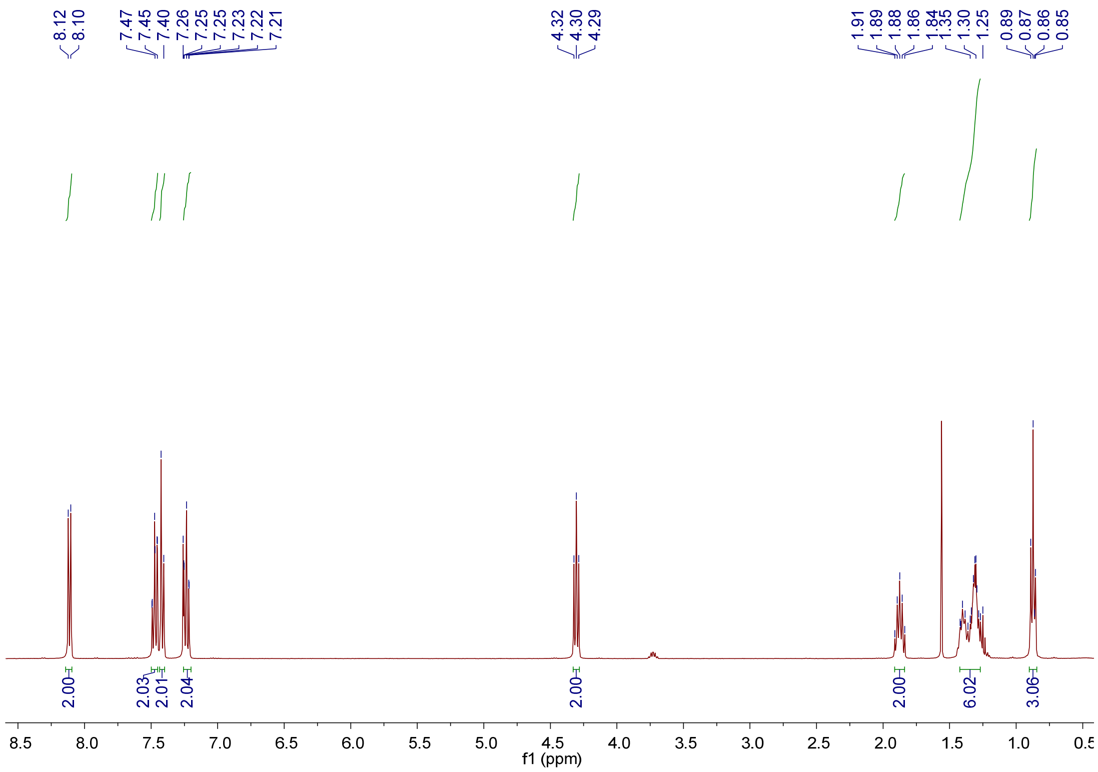

Supplementary Figure 3 | ^{1}H NMR spectrum of CzA in $CDCl_3$.

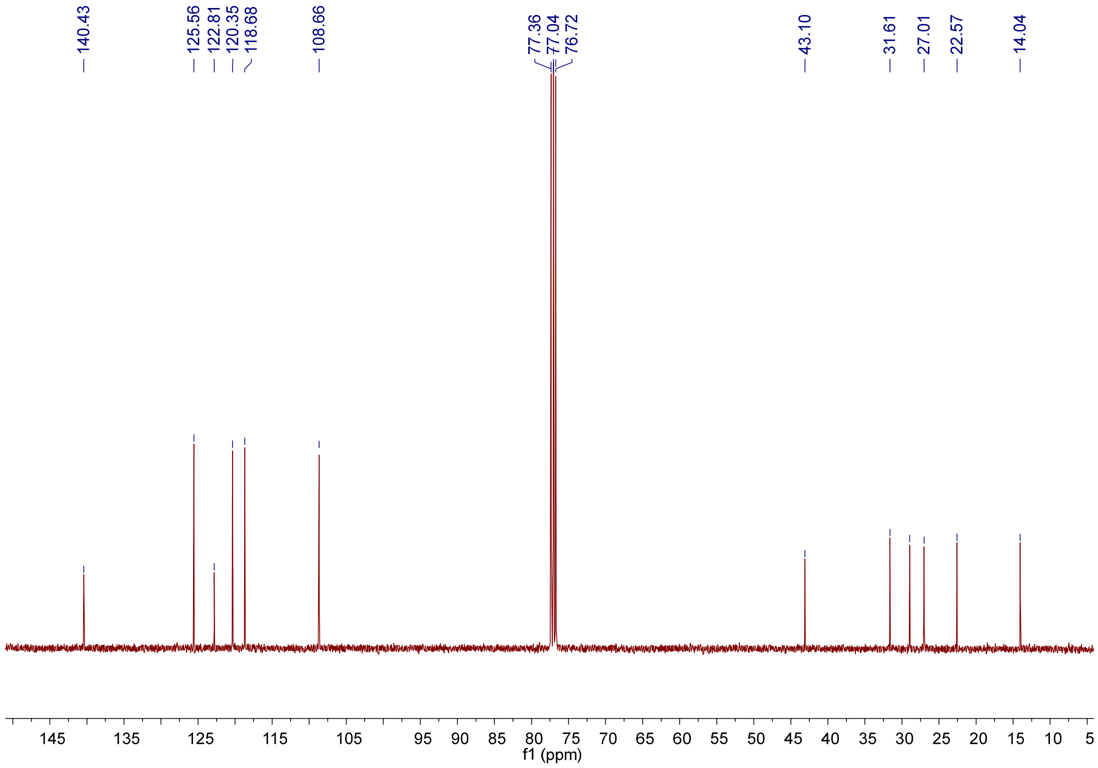

Supplementary Figure 4 | ^{13}C NMR spectrum of CzA in $CDCl_3$.

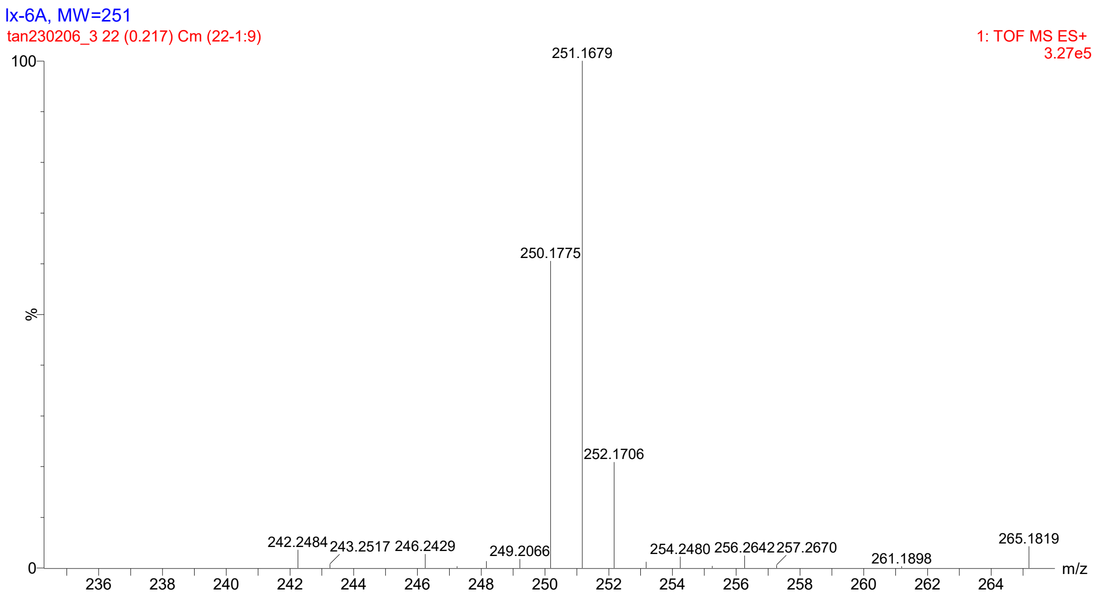

Supplementary Figure 5 | HRMS of the CzA molecule.

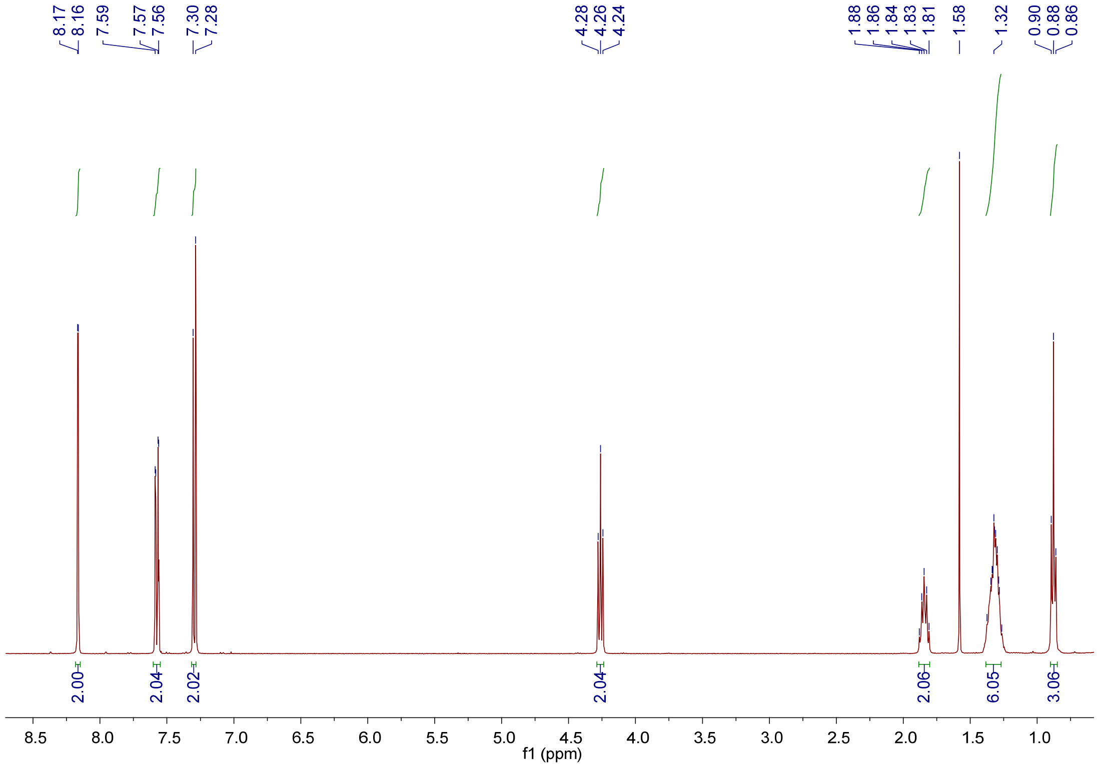

Supplementary Figure 6 | ^{1}H NMR spectrum of BrCzA in $CDCl_3$.

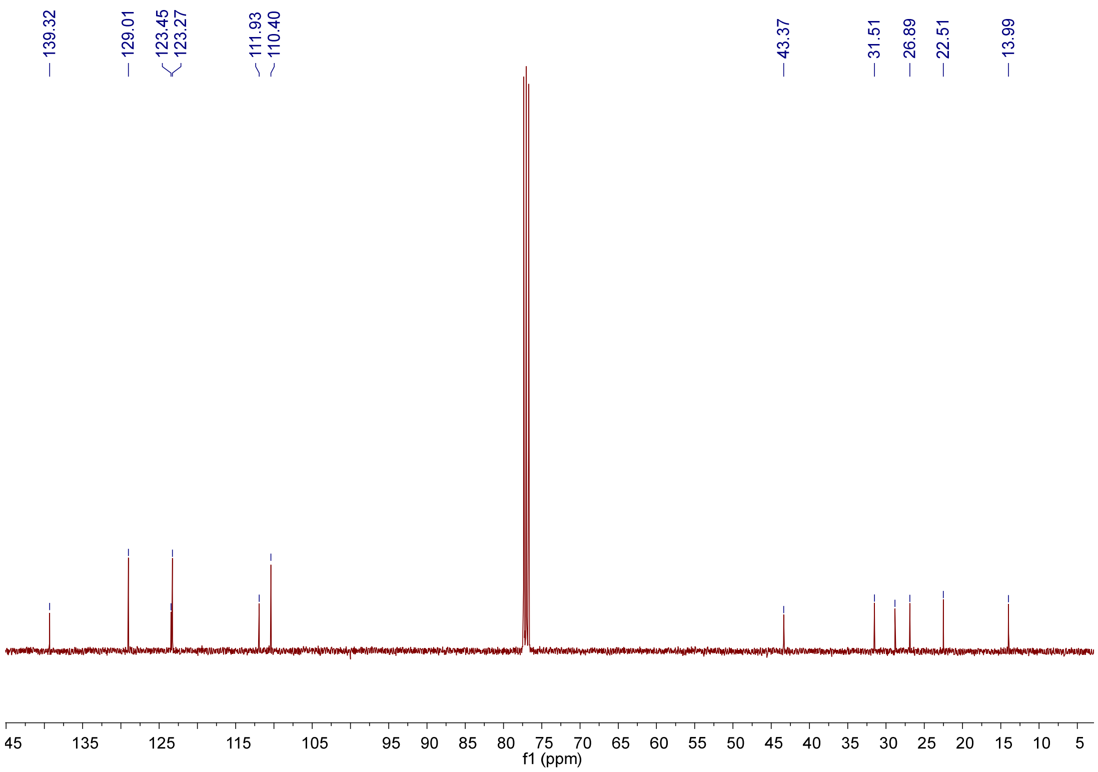

Supplementary Figure 7 | ^{13}C NMR spectrum of BrCzA in $CDCl_3$.

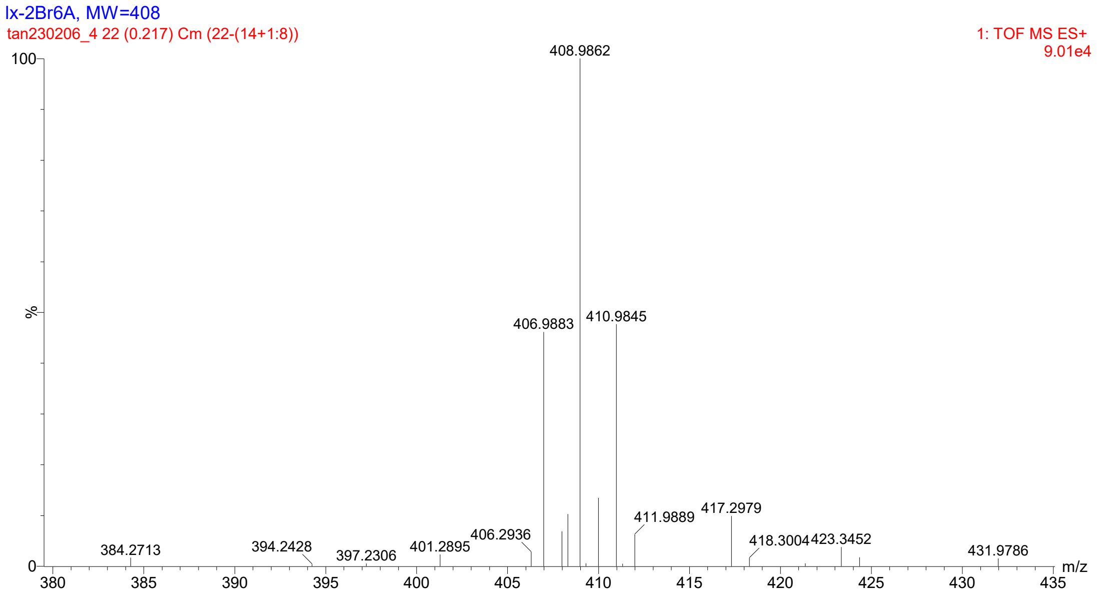

Supplementary Figure 8 | HRMS of the BrCzA molecule.

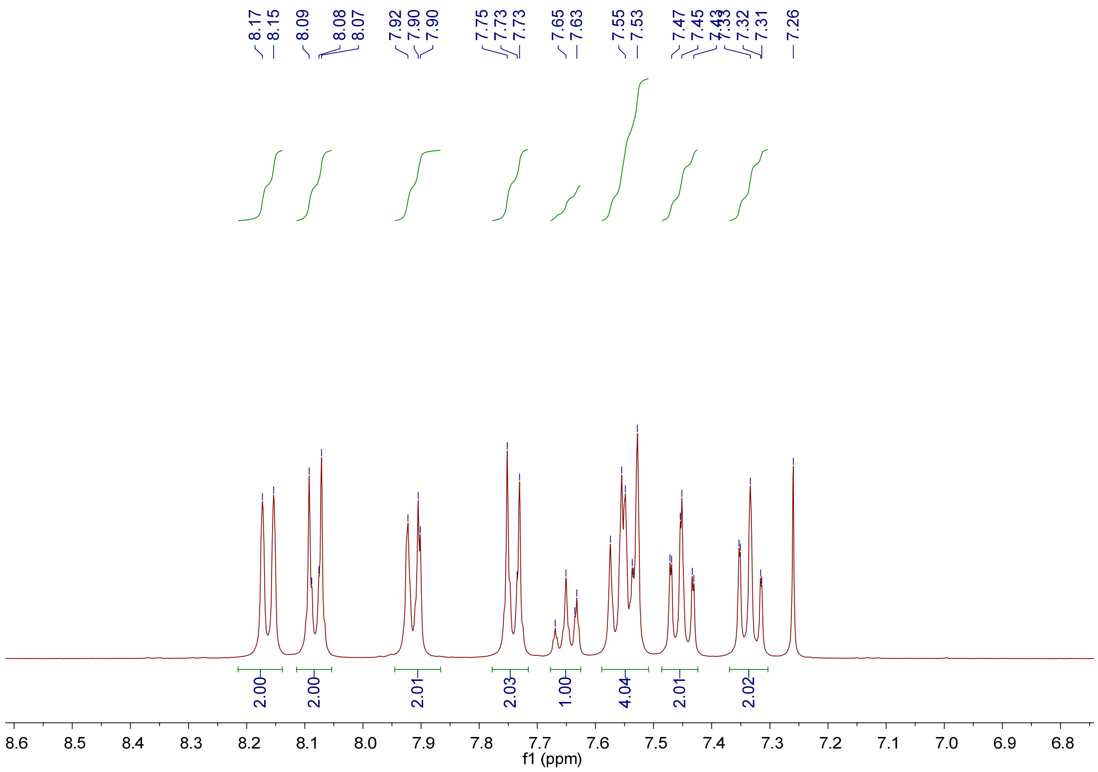

Supplementary Figure 9 | ^{1}H NMR spectrum of CzBP in $CDCl_3$.

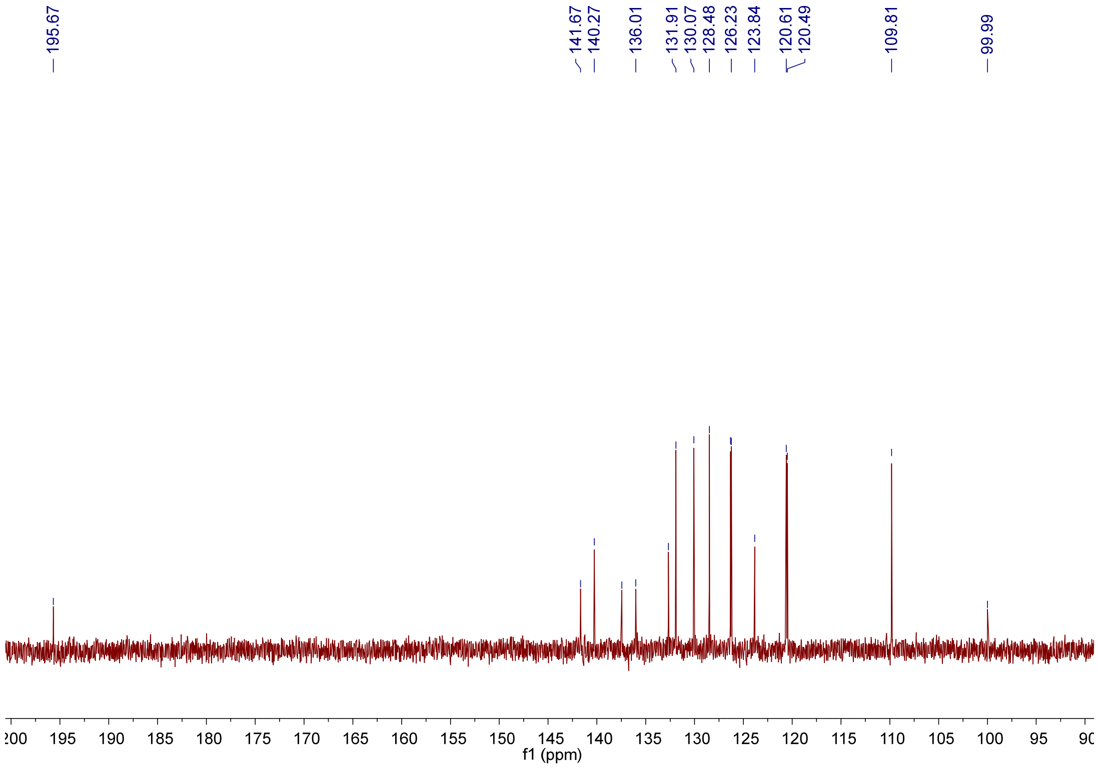

Supplementary Figure 10 | ^{13}C NMR spectrum of CzBP in $CDCl_3$.

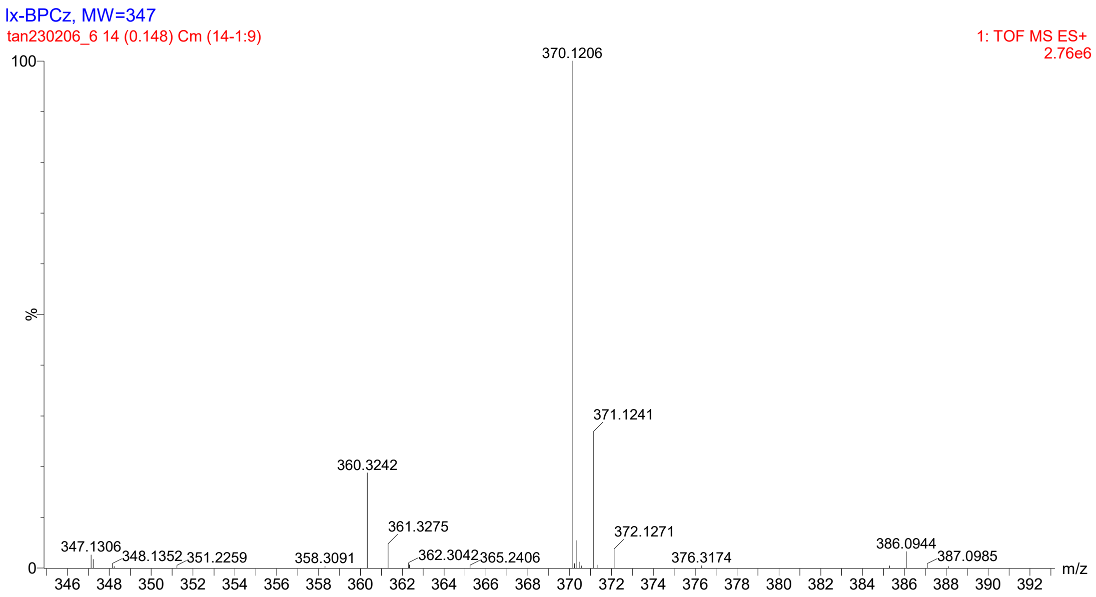

Supplementary Figure 11 | HRMS of CzBP molecules.

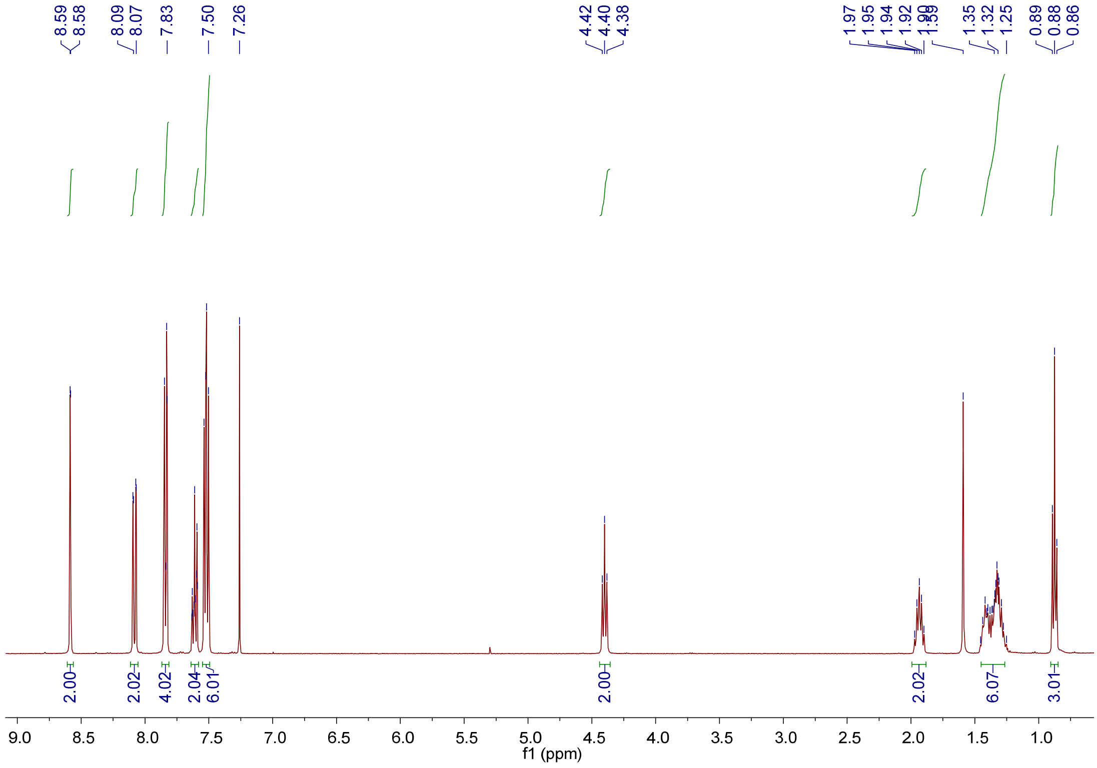

Supplementary Figure 12 | ^{1}H NMR spectrum of BPCzA in $CDCl_3$.

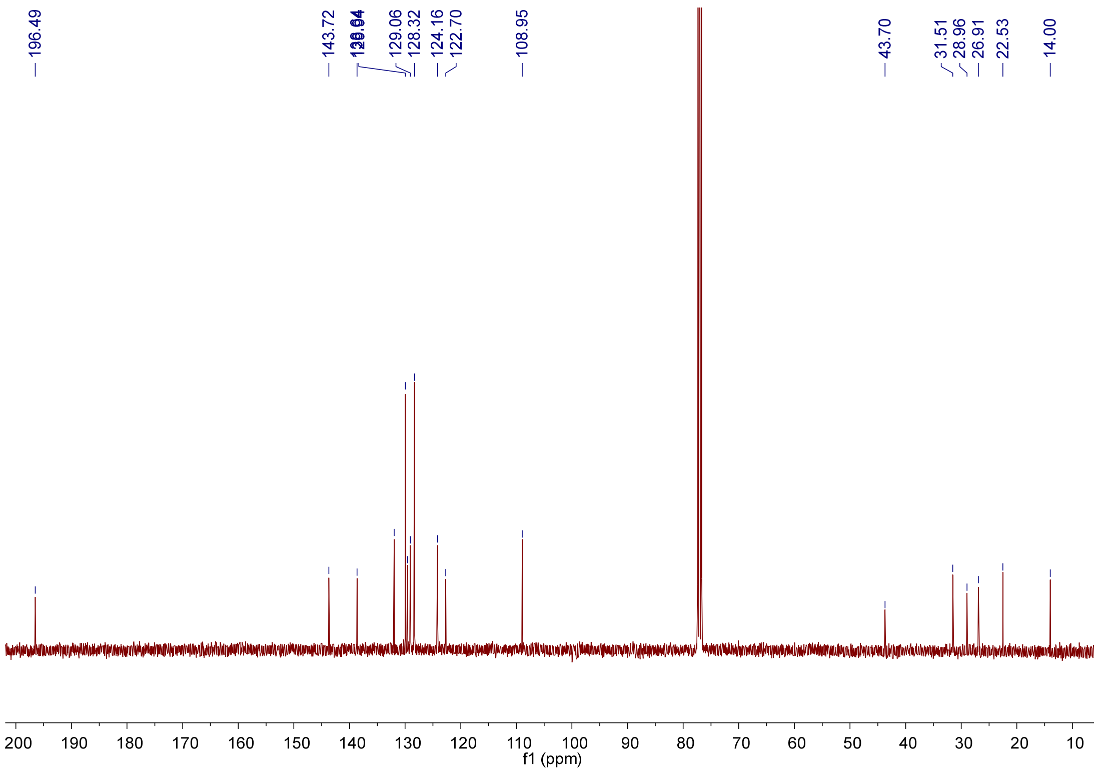

Supplementary Figure 13 | ^{13}C NMR spectrum of BPCzA in $CDCl_3$.

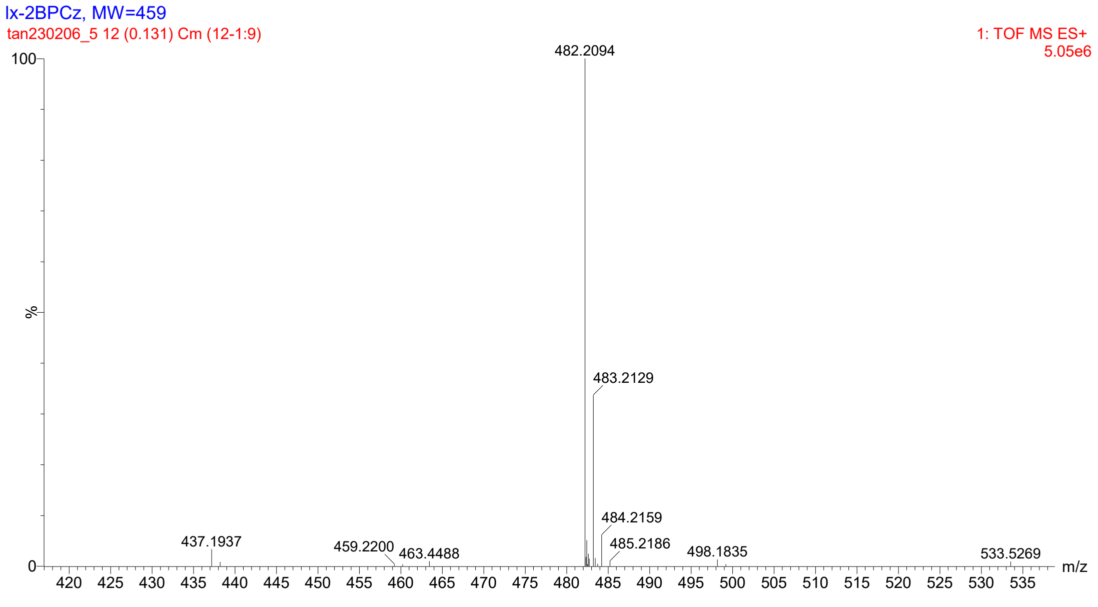

Supplementary Figure 14 | HRMS of the BPCzA molecule.

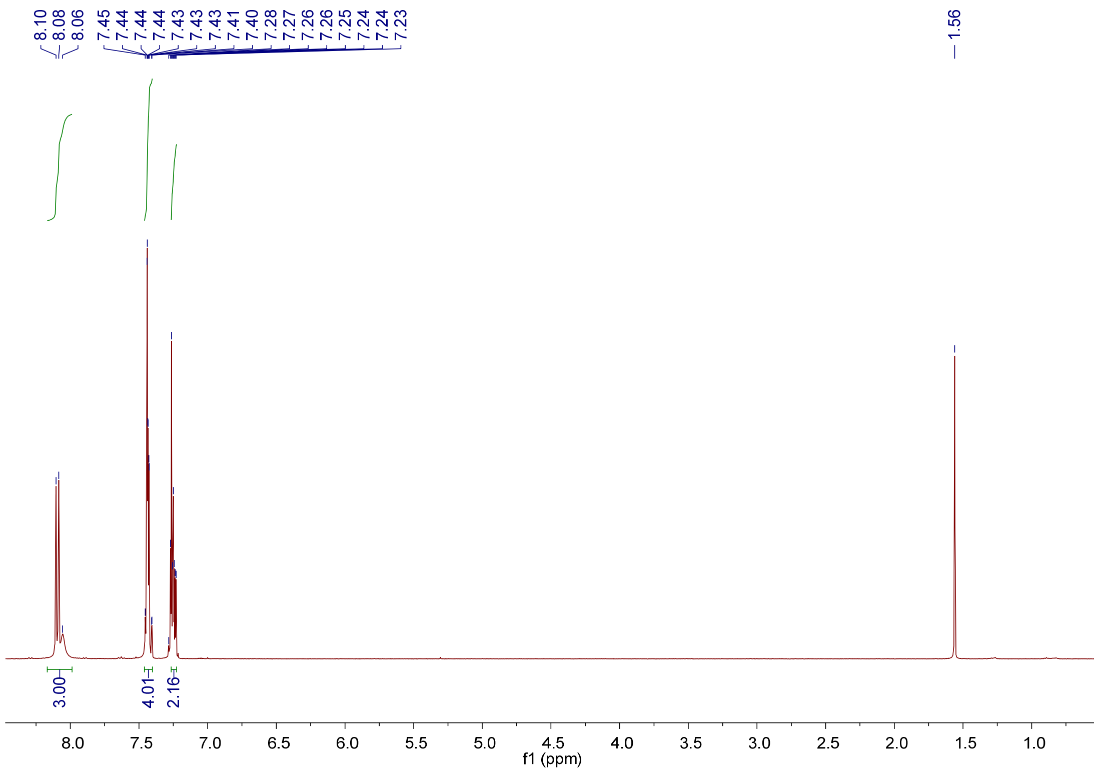

Supplementary Figure 15 | ^{1}H NMR spectrum of Cz-lab in $CDCl_3$.

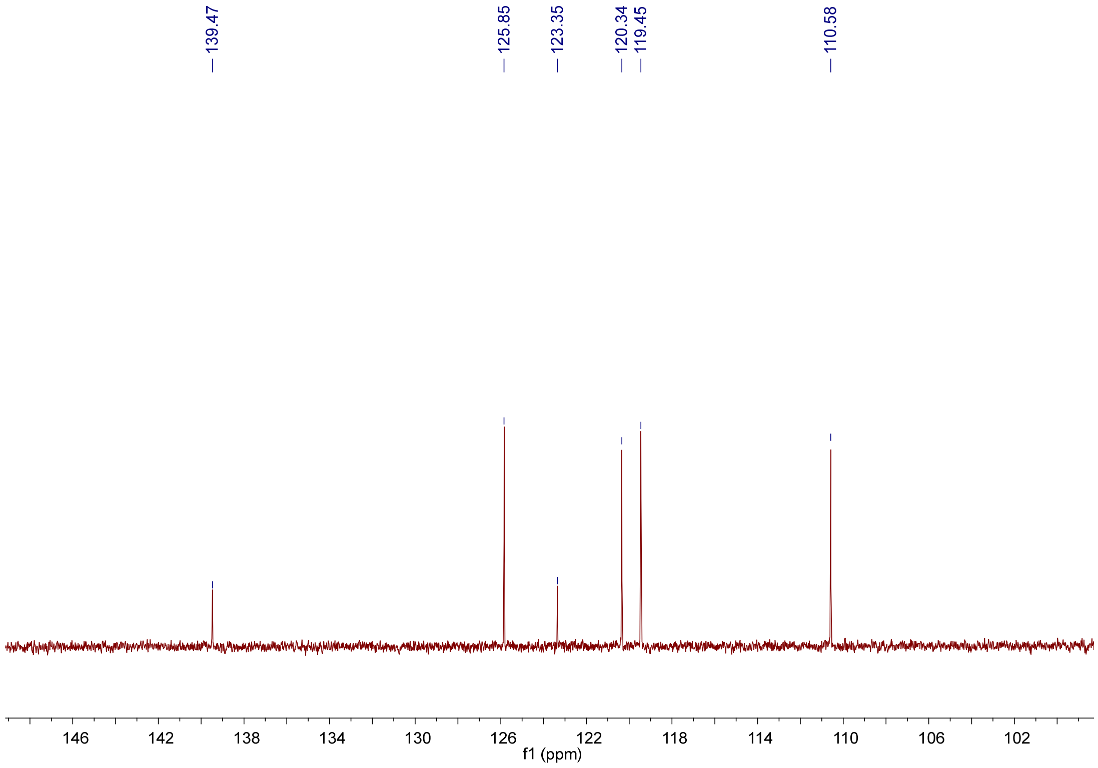

Supplementary Figure 16 | ^{13}C NMR spectrum of Cz-lab in $CDCl_3$.

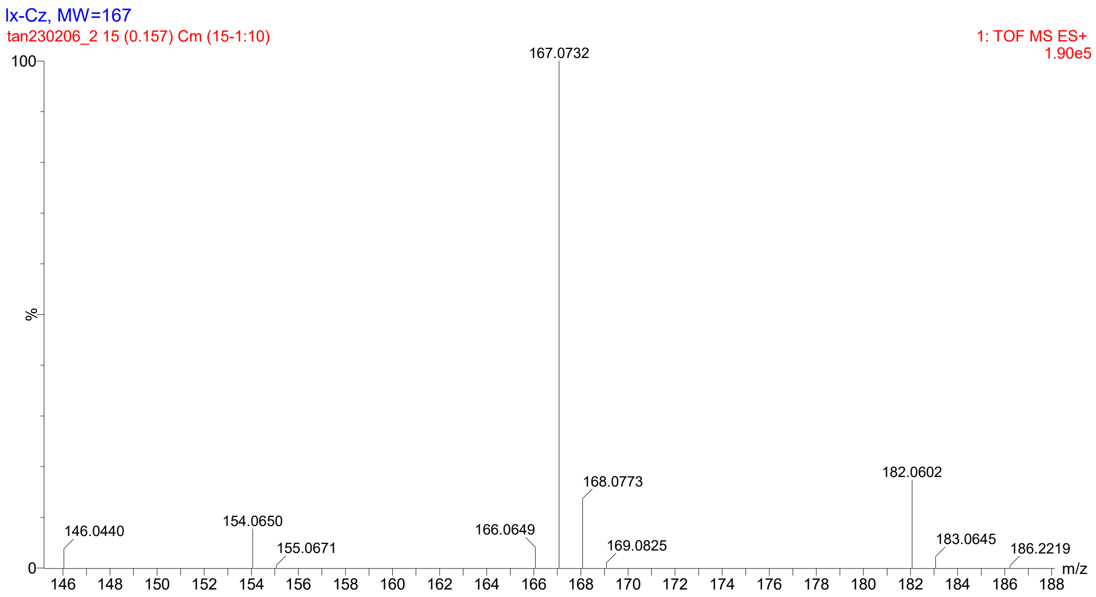

Supplementary Figure 17 | HRMS of the Cz-Lab molecule.

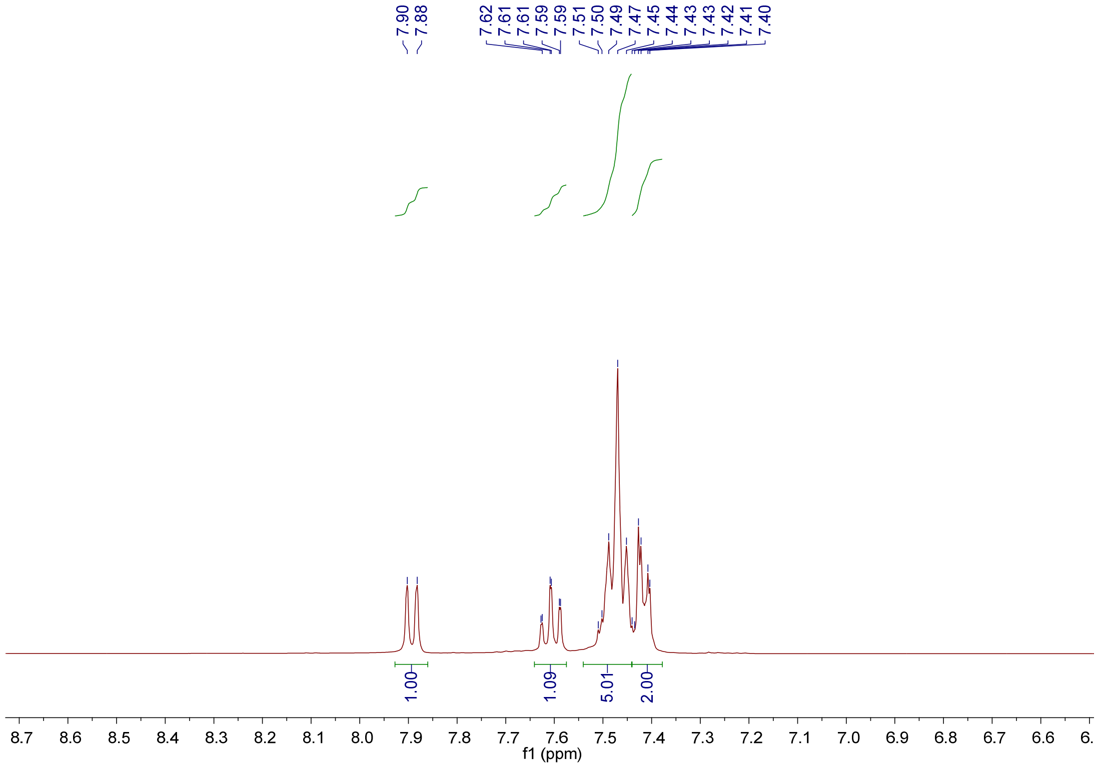

Supplementary Figure 18 | ^{1}H NMR spectrum of M in $CDCl_3$.

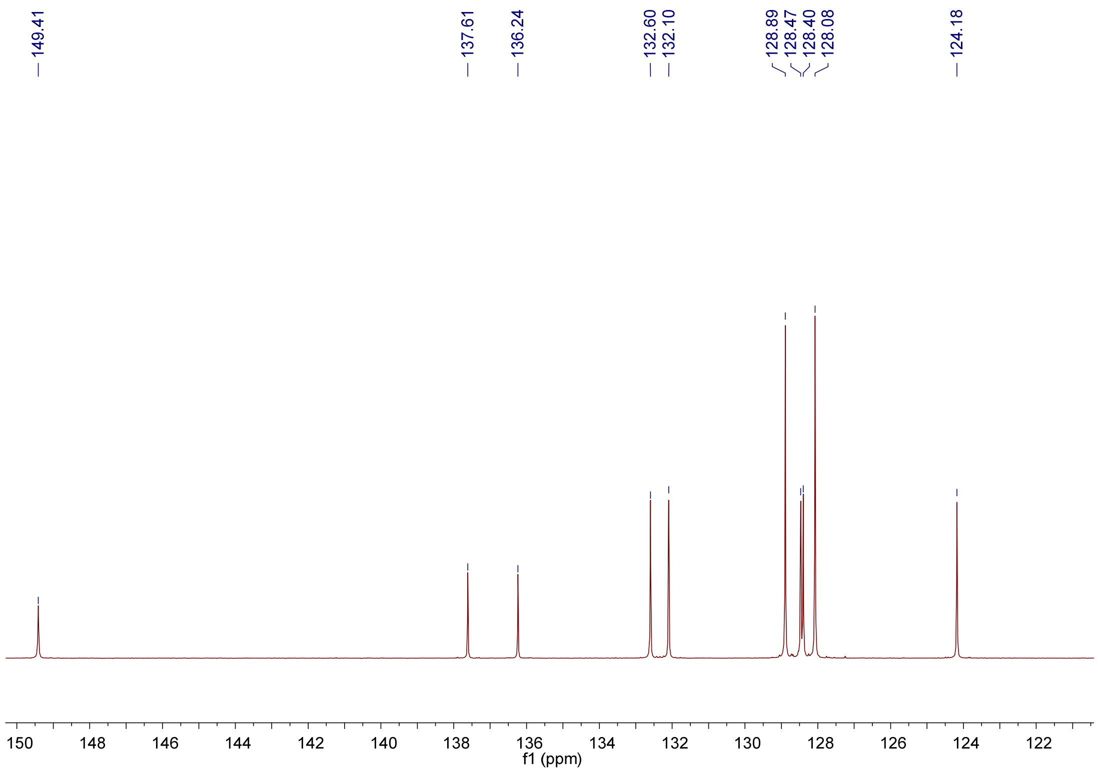

Supplementary Figure 19 | ^{13}C NMR spectrum of M in $CDCl_3$.

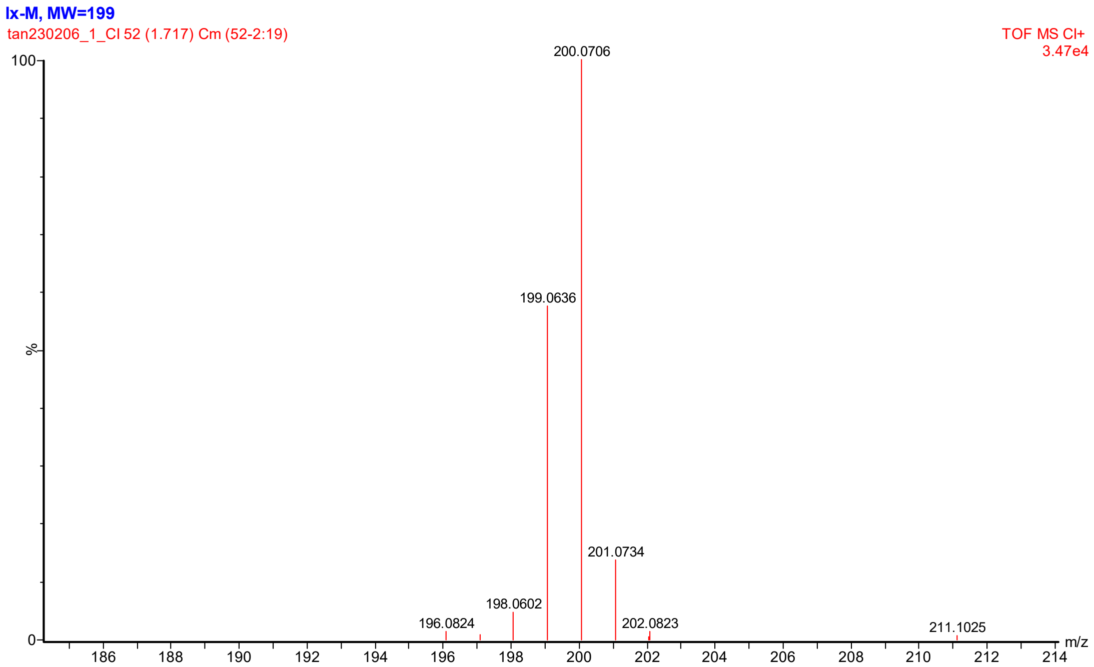

Supplementary Figure 20 | HRMS of the M molecule.

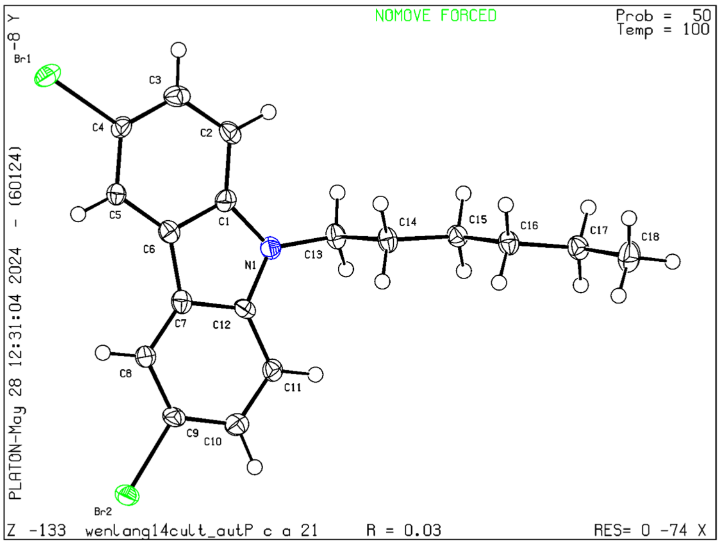

Supplementary Figure 21 | The single-crystal structure figure with probability ellipsoid of BrCzA at the 50% level. White circles represented H atoms.

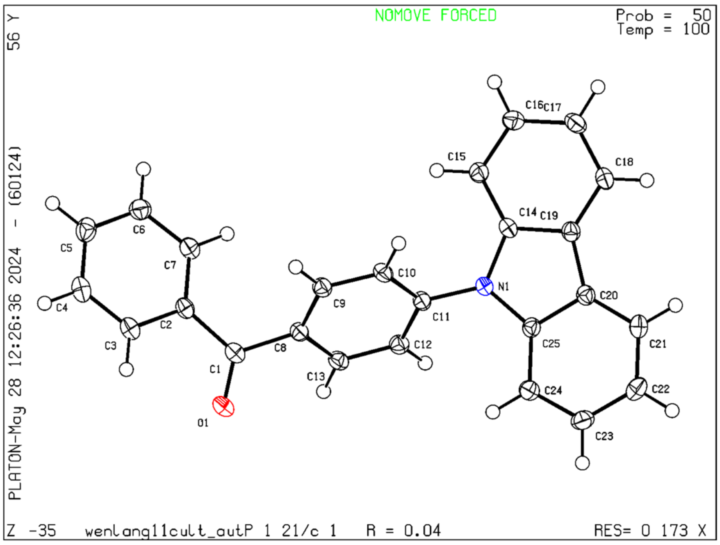

Supplementary Figure 22 | The single-crystal structure figure with probability ellipsoid of CzBP at the 50% level. White circles represented H atoms.

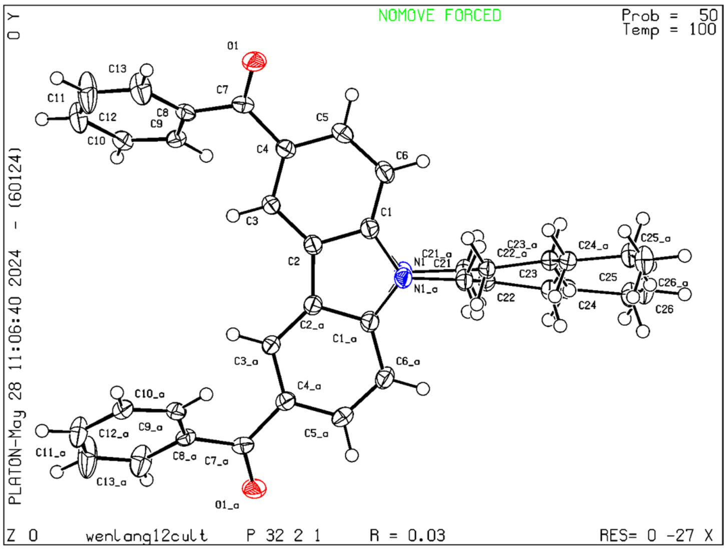

Supplementary Figure 23 | The single-crystal structure figure with probability ellipsoid of BPCzA at the 50% level. White circles represented H atoms.

Purity, thermal properties, and crystal structure analysis

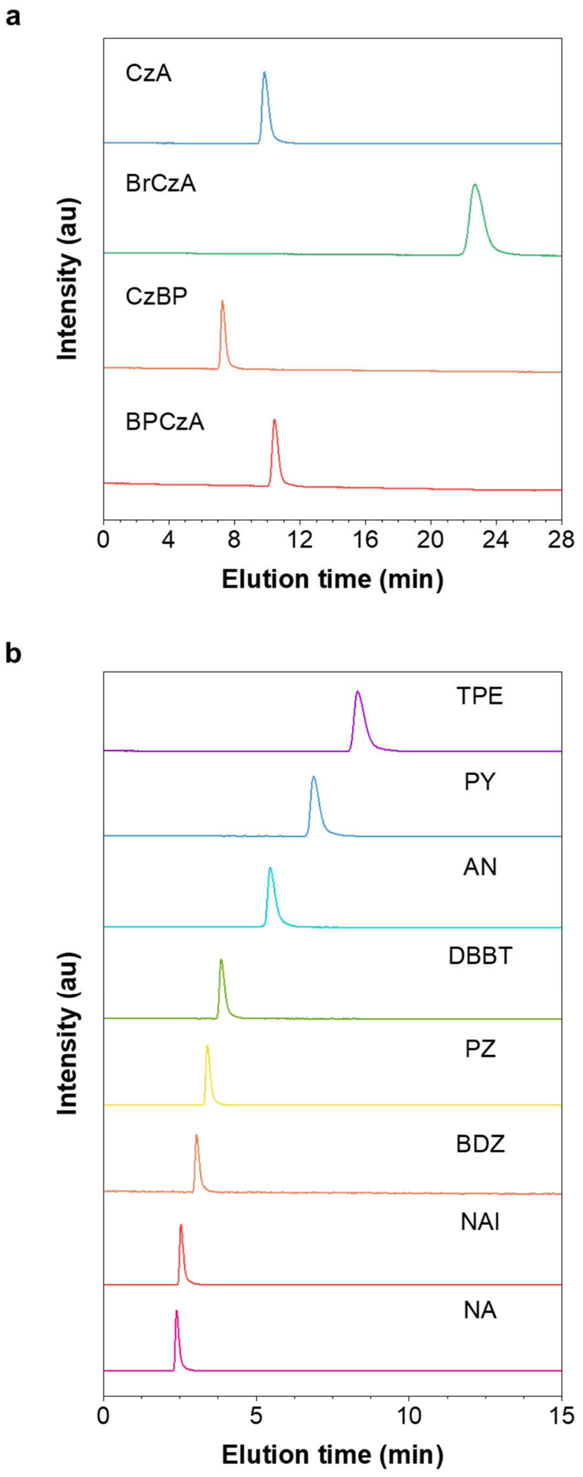

Supplementary Figure 24 | HPLC spectra of the host (a) and guest (b) molecules used in this work with acetonitrile/water as eluent in ratios of 9:1 (v/v).

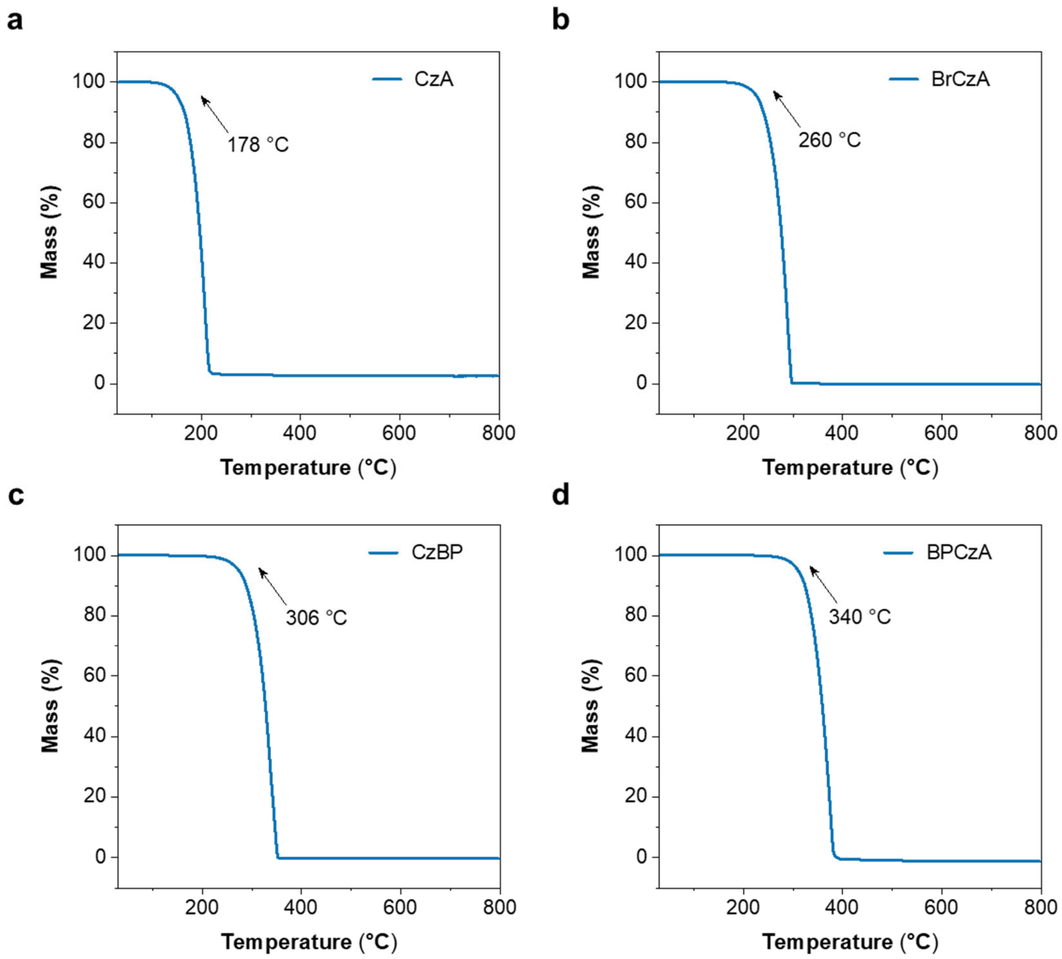

Supplementary Figure 25 | TGA curves of host molecules, CzA (a), BrCzA (b), CzBP (c), BPCzA (d). Decomposition temperature T_d was noted.

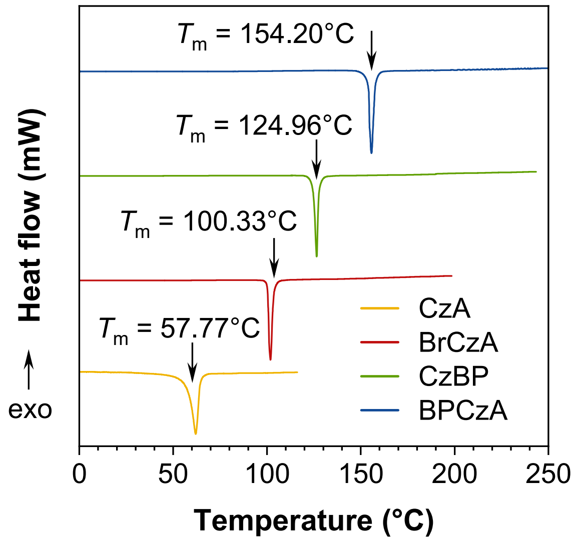

Supplementary Figure 26 | DSC curves of host molecules. Melting temperature T_m was noted.

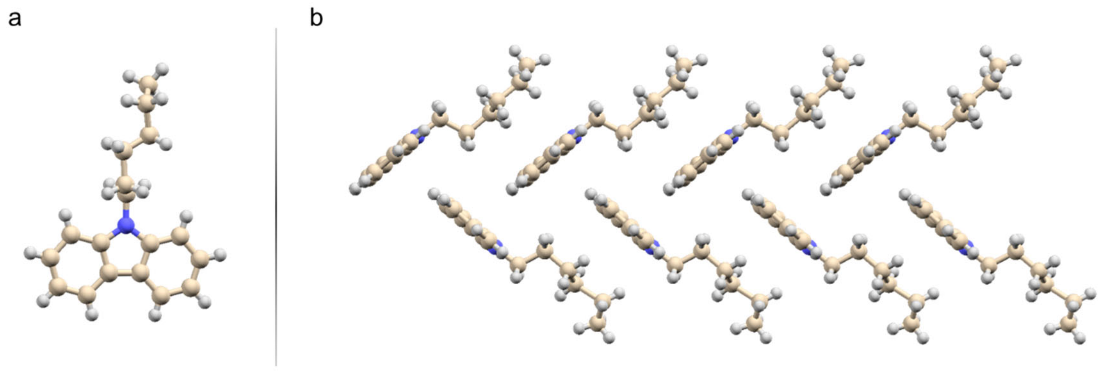

Supplementary Figure 27 | Molecular structure (a) and packing (b) in the single-crystal structure of CzA. Color code: tan, C; blue, N; gray, H.

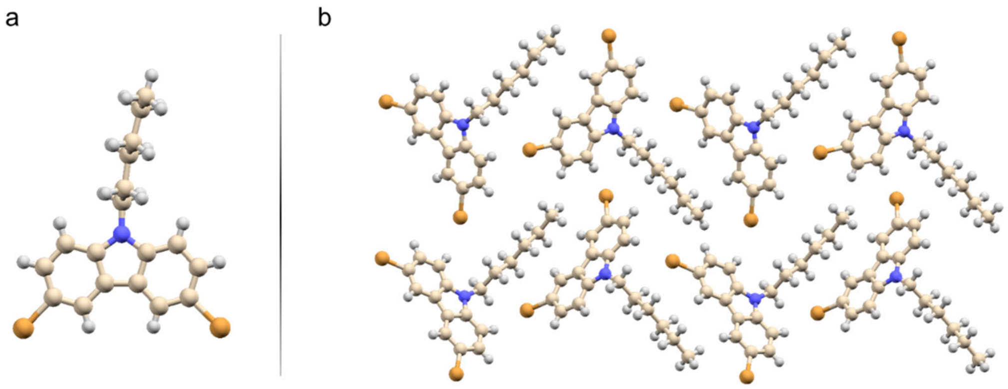

Supplementary Figure 28 | Molecular structure (a) and packing (b) in the single-crystal structure of BrCzA. Color code: tan, C; blue, N; yellow, Br; gray, H.

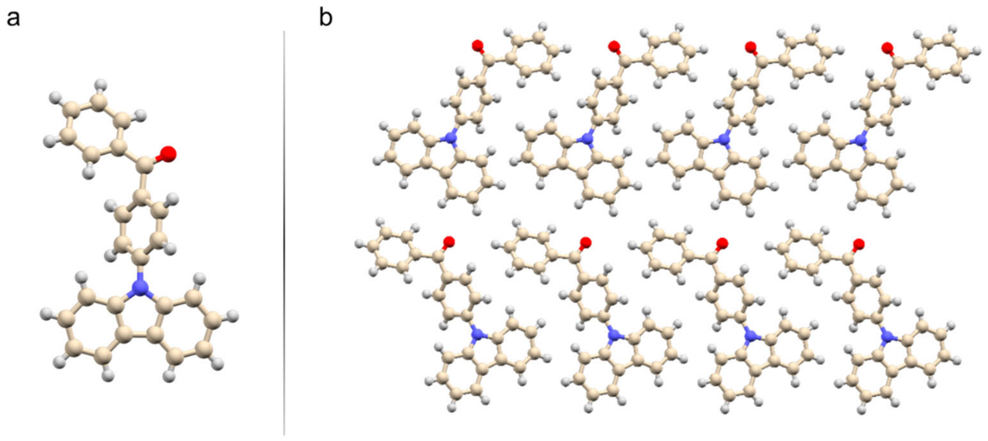

Supplementary Figure 29 | Molecular structure (a) and packing (b) in the single-crystal structure of CzBP. Color code: tan, C; blue, N; red, O; gray, H.

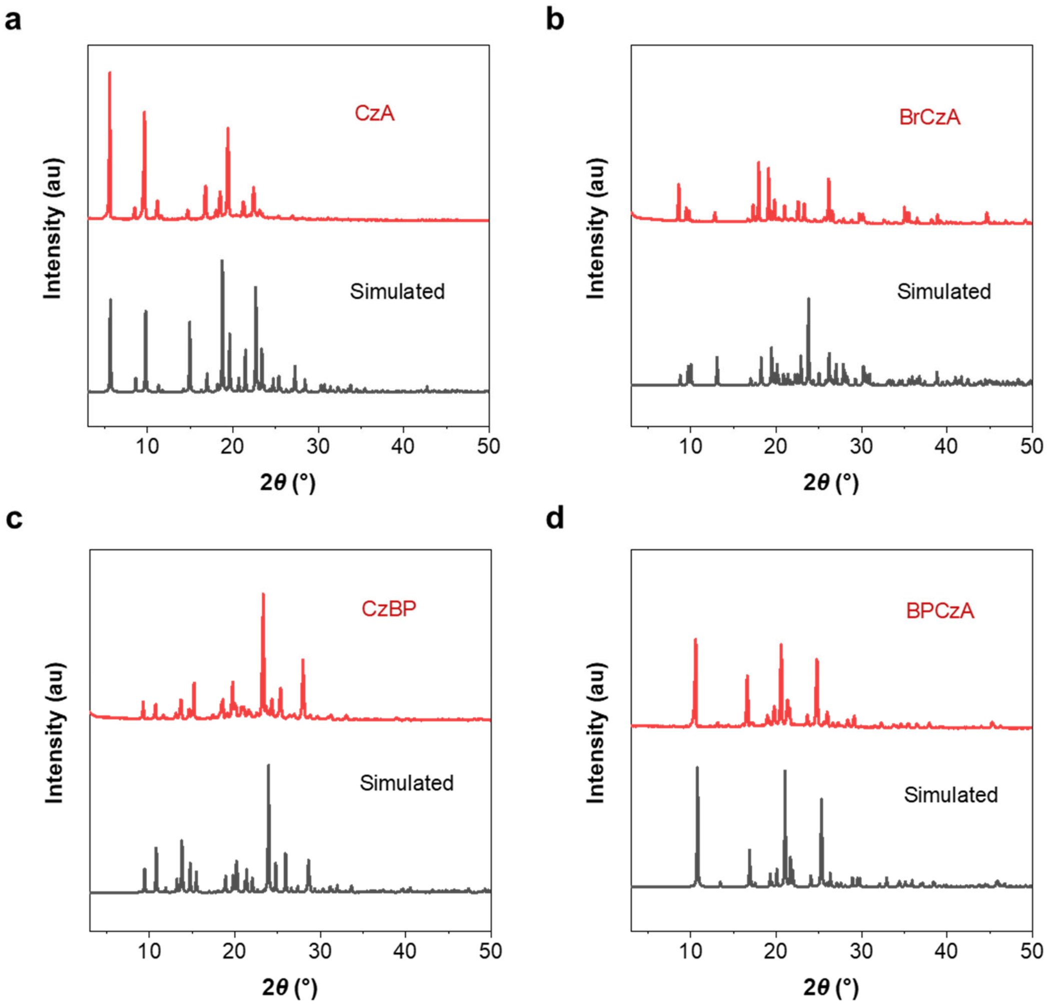

Supplementary Figure 30 | PXRD patterns of host molecules, CzA (a), BrCzA (b), CzBP (c), BPCzA (d). Simulated patterns based on their single crystals were attached for reference.

III. Additional information for photophysical mechanism study

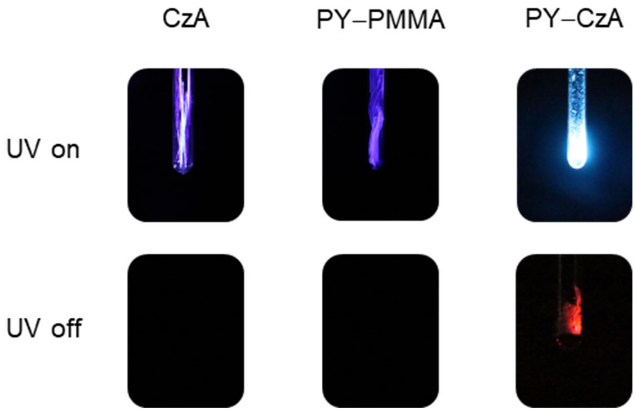

Supplementary Figure 31 | Photographs of CzA crystals, PY−PMMA film, and PY−CzA crystals taken with the 365 nm UV lamp turned on and off.

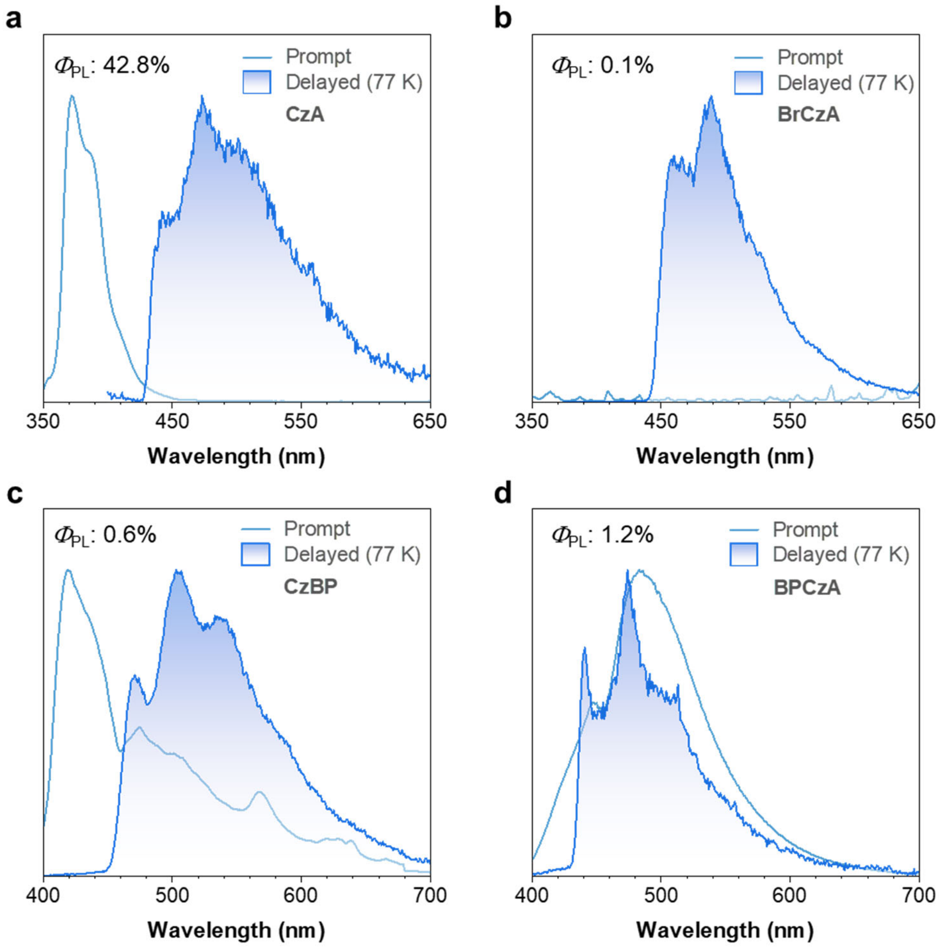

Supplementary Figure 32 | Prompt and delayed PL spectra of host molecules in the crystal state, CzA (a), BrCzA (b), CzBP (c), BPCzA (d). Delayed spectra were measured at 77 K with a delay time of 10 ms. All host molecules showed negligible RTP at room temperature. Their photoluminescence quantum yields measured at room temperature was noted.

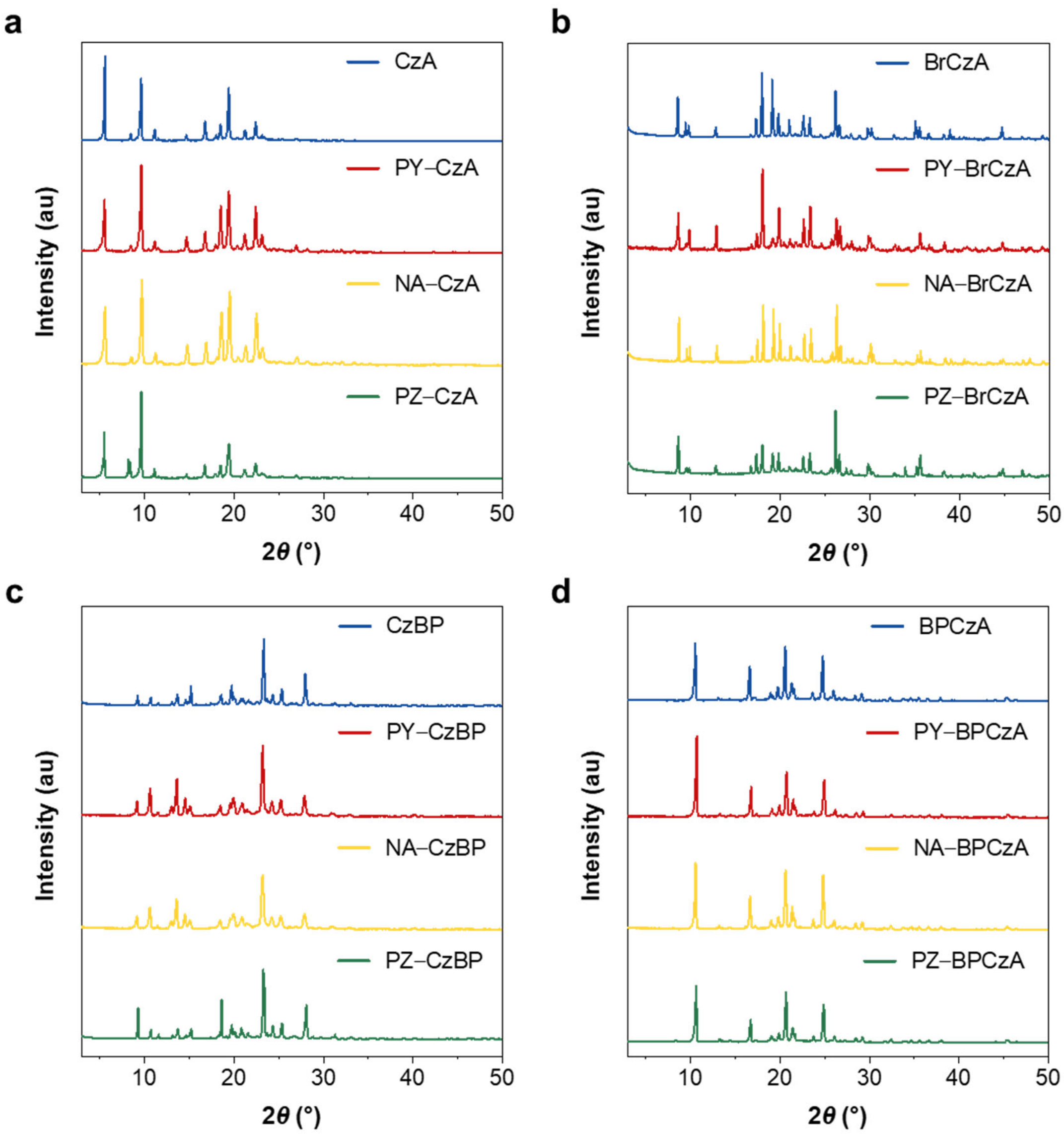

Supplementary Figure 33 | PXRD patterns of host–guest crystals with PY, NA and PZ as guests, respectively. CzA system (a), BrCzA system (b), CzBP system (c), BPCzA system (d). PXRD patterns of corresponding host molecules were displayed for reference.

PXRD patterns of host−guest crystals doped with different guest molecules all exhibited the same pattern as their corresponding host crystals, indicating the doping of guest with the ratio of 1.0% would not change the molecular packing of host crystals.

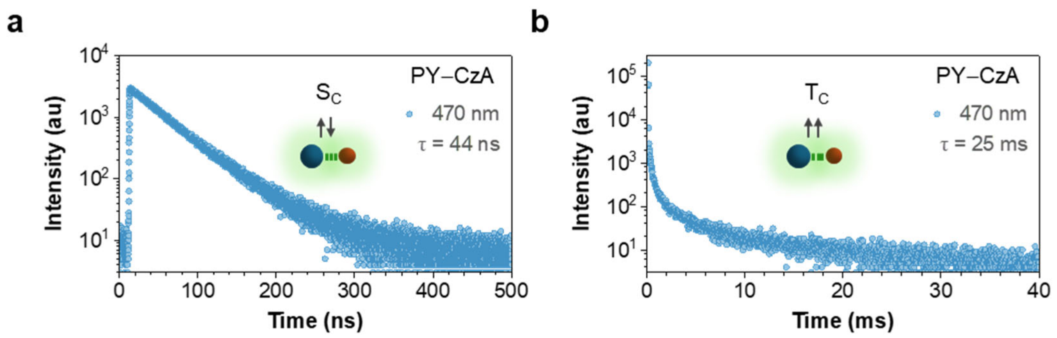

Supplementary Figure 34 | Lifetime curves of complex in the singlet state (a) and triplet state (b) in PY−CzA system.

Fs-TA spectroscopy

In Supplementary Fig. 35a, the 3D contour map of fs-TA spectra for pure CzA crystals exhibited an excited-state absorption (ESA) peak decaying at 650 nm, which was same as the initial ESA peak in PY−CzA crystals (Fig. 3a). However, the ESA peak in PY−PMMA film at 472 nm was not found in PY−CzA crystals (Supplementary Fig. 35b, and 36). Instead, a new ESA peak at 505 nm emerged (Fig. 3a and 3b). Thus, we assigned this new peak to be the ESA peak of complex. Due to the low resolution of TA spectra of PY−PMMA film, PY in acetonitrile solution was also measured for reference (Supplementary Fig. 35c, and 36).

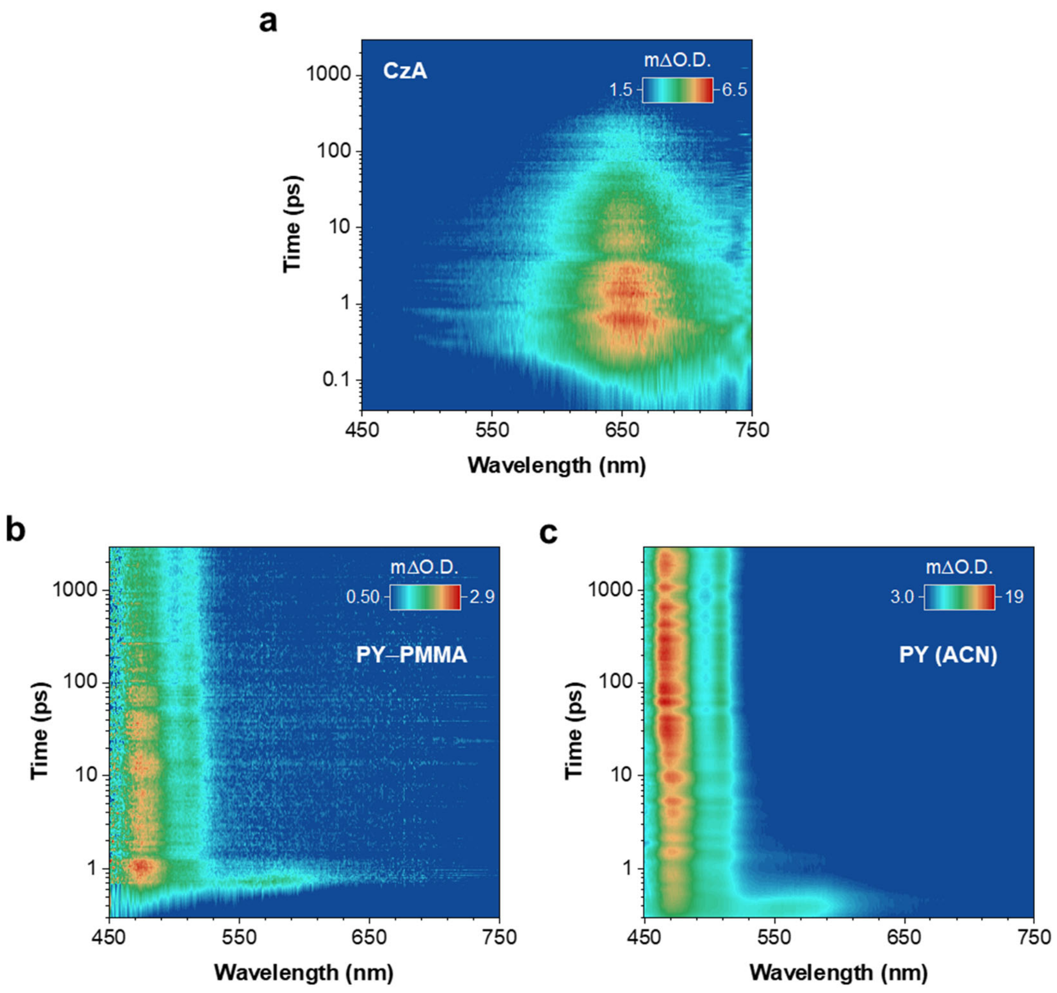

Supplementary Figure 35 | 3D contour map of fs-TA spectra for CzA crystals (a), and PY−PMMA film (b), and PY in acetonitrile solution (c), upon 267 nm excitation.

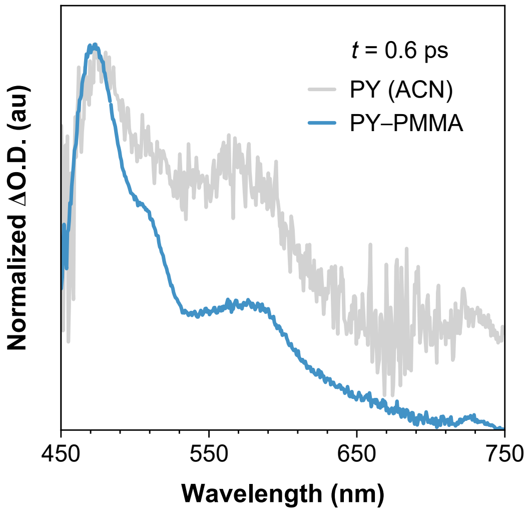

Supplementary Figure 36 | TA spectra of PY−PMMA film and PY in acetonitrile solution at a pump-probe delay time of 0.6 ps upon 267 nm excitation.

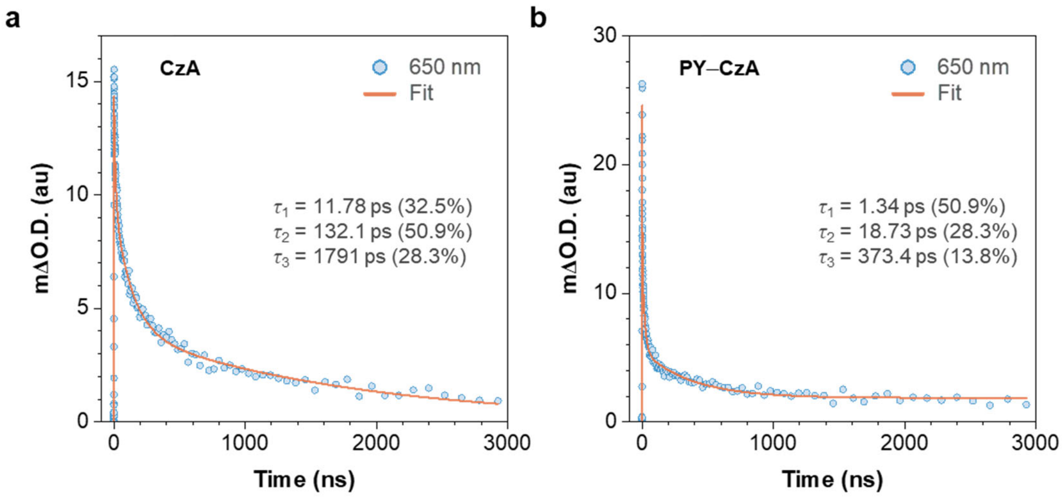

Supplementary Figure 37 | Kinetic fitting of excited-state absorption for pure CzA crystals and PY−CzA crystals at 650 nm.

Monitoring of P_G generation

In the prompt PL spectra of PY–CzA crystals (Supplementary Fig. 38a), fluorescence of host and guest were observed at around 400 nm and emission of complex, L_C, exhibited a broad peak between 430 nm to 700 nm. The weak phosphorescence, P_G, at 595 nm was hidden by bright emission of complex, remaining indistinguishable in the spectra. However, P_G could be detected when the emission kinetics were measured. In the range of 50 ns after excitation, L_C showed a slower decay at 595 nm than that at 470 nm (Supplementary Fig. 38b). This slight difference was due to the existence of P_G at 595 nm. Thus, by subtracting the intensity at 470 nm ($\boldsymbol{I}_{470}$) from that at 595 nm ($\boldsymbol{I}_{595}$) at each time point, the P_G generation process in 50 ns could be monitored (Supplementary Fig. 38c).

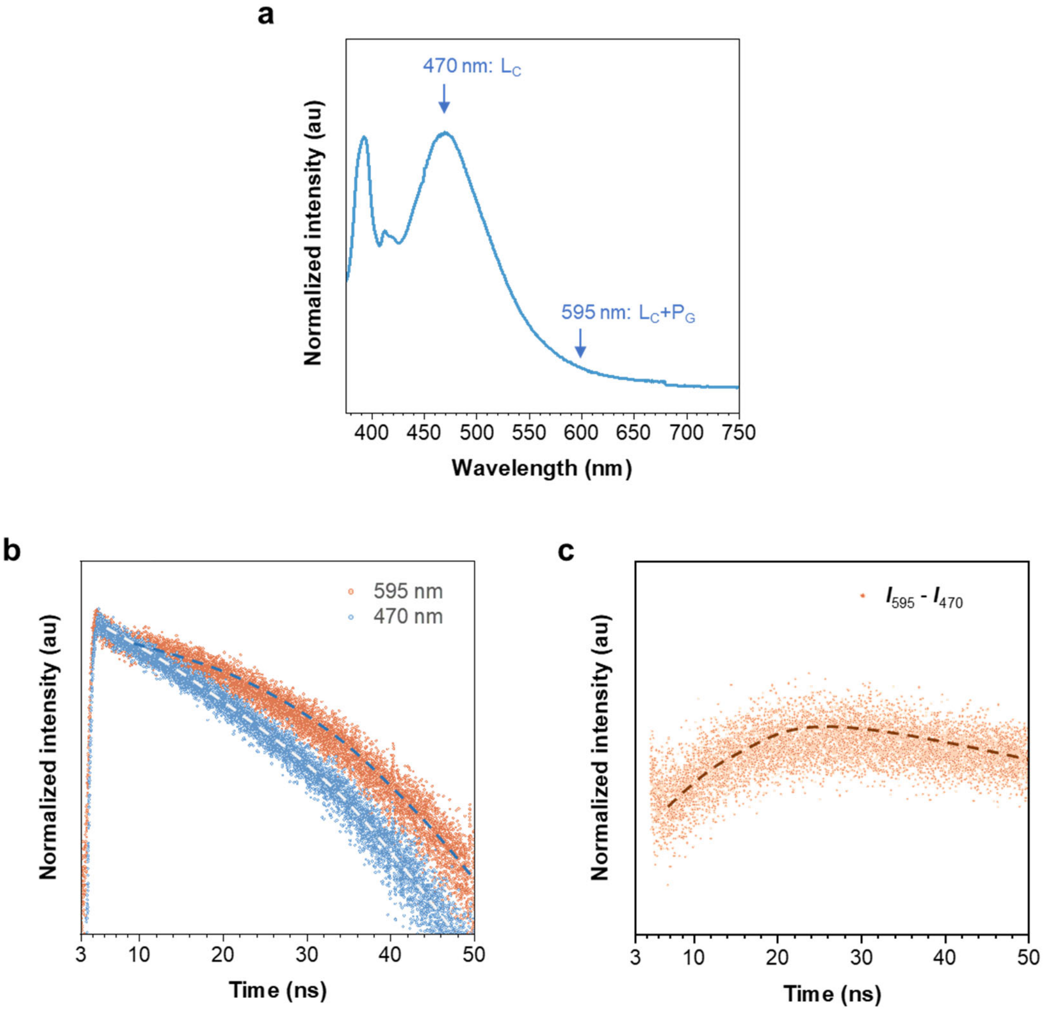

Supplementary Figure 38 | Monitoring of P_G generation process in PY–CzA crystals.

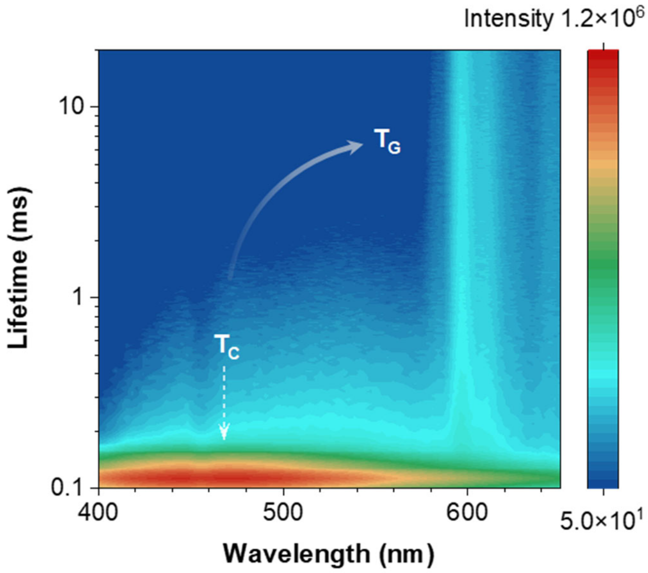

Supplementary Figure 39 | TRES mapping of PY−CzA crystals excited at 365 nm. A gradual emission shift from L_C (470 nm) to P_G (595 nm) was observed, indicating excitons transferred from T_C to T_G.

Temperature-dependent PL spectroscopy

At 77 K, PY−CzA exhibited two emission bands: one located between 420 and 540 nm, which was a mixture of phosphorescence from host crystals (P_H) and complex emission (L_C); and the other between 590 and 750 nm, originating from the phosphorescence of guest molecules (P_G). The non-radiative transitions from the triplet states were sufficiently suppressed at low temperature, resulting in P_H dominating at 77 K, while P_G was weak due to the low doping concentration of PY and its poor ISC ability. As temperature increased, both P_H and P_G slightly decreased due to the enhanced thermal vibrations. When the temperature increased to 200 K, P_H disappeared, and phosphorescence from the triplet state of complex, L_C, became observable at around 470 nm. Simultaneously, the intensity of phosphorescence of guest, P_G, started to increase. This indicated the presence of complex favored the formation of phosphorescence. As the temperature continued to rise, L_C kept weakening while P_G dramatically intensified, proving the energy transfer process from T_C to T_G was activated at elevated temperature (Supplementary Fig. 40a,b). This thermally activated process was also supported by the lifetime curves of P_G recorded at different temperature, where the component ratio of P_G kept increasing as temperature elevated (Supplementary Fig. 40c). Thus, at low temperatures, the ET process was suppressed, and the excitons were trapped in T_C, and the system emitted phosphorescence of host or complex. At elevated temperatures, the ET process was activated, and the excitons were transferred to T_G, resulting in the emission of P_G (Supplementary Fig. 40d).

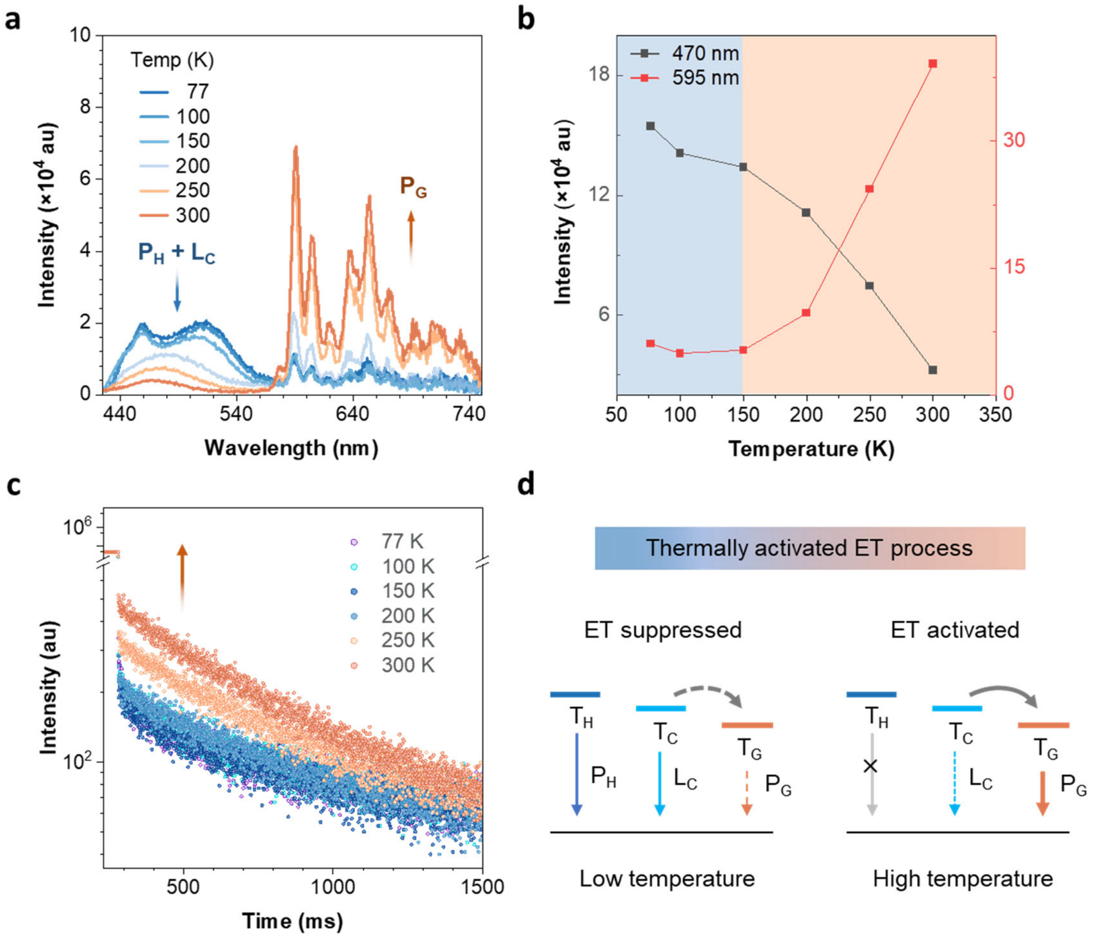

Supplementary Figure 40 | Variable temperature study on PY-CzA system. (a) Temperature-dependent delayed PL spectra of PY−CzA at a delay time of 10 ms, excited at 365 nm. (b) The intensity of L_C (470 nm) and P_G (595 nm) at different temperature. (c) Lifetime curves of P_G at different temperature, recorded at 595 nm. (d) Schematic diagram of thermally activated ET process during the variable temperature experiment in PY−CzA system.

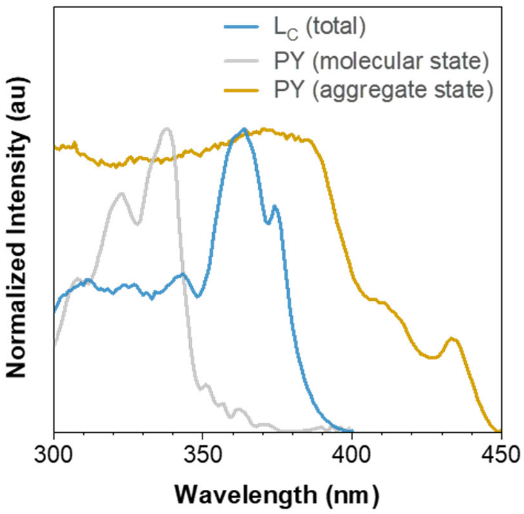

Supplementary Figure 41 | Comparison between excitation spectrum of PY−CzA measured at L_C, excitation spectra of PY measured at molecular state and aggregate state. For molecular state, excitation spectrum of PY−PMMA (1.0 wt%) was measured with emission recorded at 410 nm. For aggregate state, excitation spectrum of PY crystals was measured with emission recorded at 475 nm.

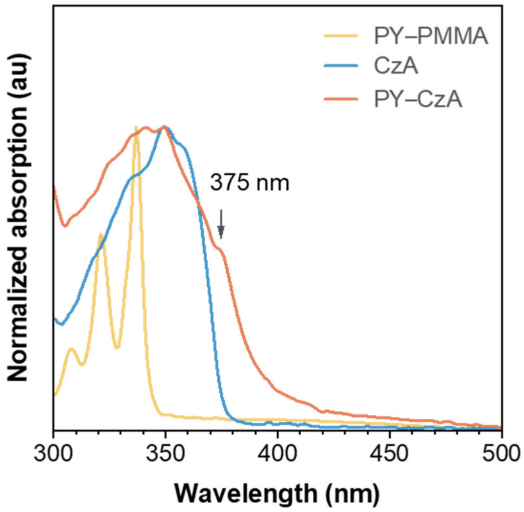

Supplementary Figure 42 | UV-Vis absorption spectra of PY−PMMA, CzA crystals and PY−CzA crystals.

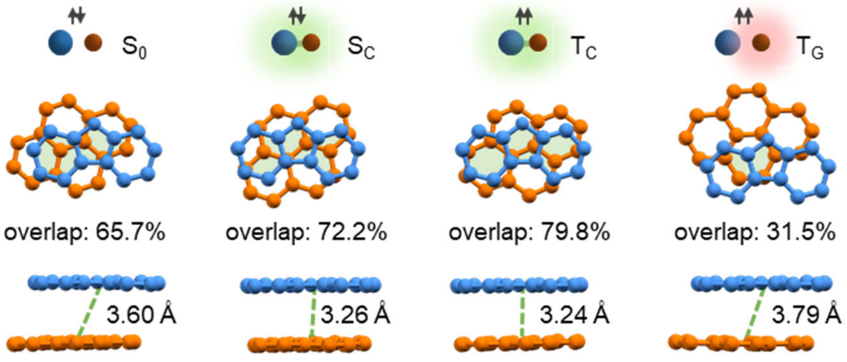

Supplementary Figure 43 | Optimized conformations of PY/CzA dimer in the ground state (S_0), singlet state of complex (S_C), triplet state of complex (T_C), and triplet state of guest (T_G), from the top view (top) and side view (bottom). Color code: blue, CzA; red, PY. Hydrogen atoms and hexyl groups were hidden for clear visualization. Overlap areas between aromatic ring planes were colored in light green. Overlap ratios and center-to-center distances were labeled.

The conformation of PY/CzA dimer was optimized at S_0, S_C, T_C, and T_G state, respectively, to stimulate the excited-state behavior of complex (Supplementary Fig. 43). Compared to S_0, complex at S_C and T_C showed increased proximity and overlap of CzA and PY, indicating the occurrence of coupling process. However, in the T_G state, CzA and PY were noticeably misaligned, which significantly reduced the extent of overlap and increased the center-to-center distance. This demonstrated a decoupling process. The intermolecular noncovalent interactions (NCI) analysis also revealed the presence of stronger π–π interactions at S_C and T_C compared to S_0, which became weakened after the transition to T_G (Supplementary Fig. 44).

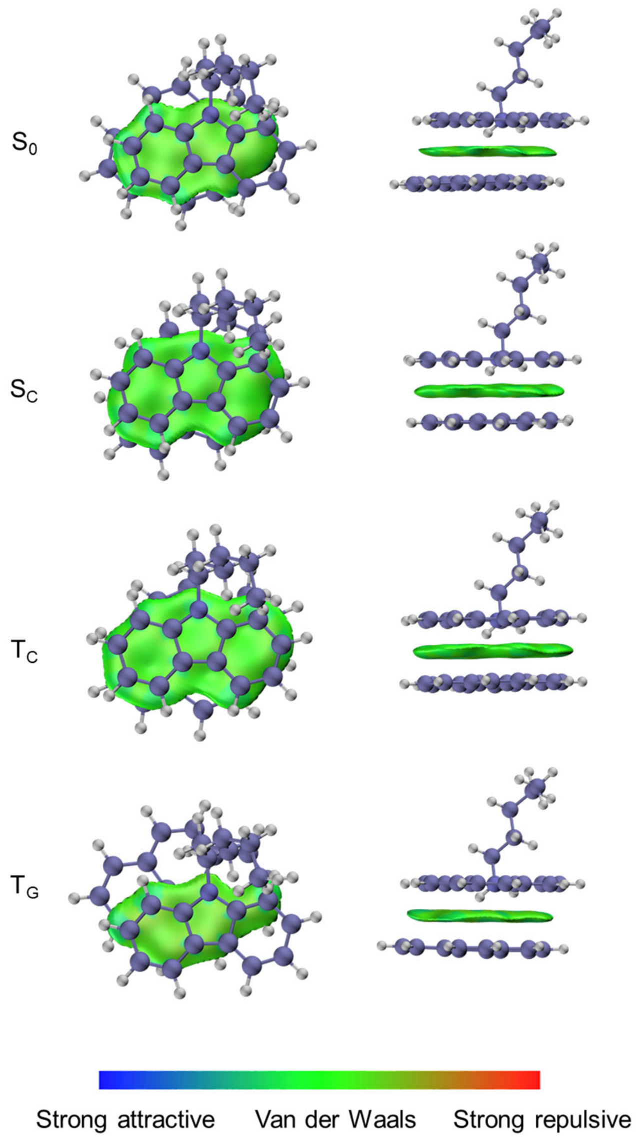

Supplementary Figure 44 | The distributions of NCI regions in PY/CzA dimer in the different states from the top view (left) and side view (right). Color code: ice blue, C; gray, H; red, O. The green areas represented the π–π interactions between the phenyl ring and carbazole moiety.

IV. Modulation of coupling strength through pressurization

Photophysical investigation on PY−BrCzA systems

As shown in Supplementary Fig. 45, the newly aroused peak, L_C, in the prompt PL spectra, suggested that coupling process also occurred in PY−BrCzA system, and the delayed PL spectrum matched well with the phosphorescence of PY. The enhanced P_G in prompt PL spectrum was due to the heavy atom effect (HAE) of bromine in the host molecule BrCzA. HAE accelerated the rate constant of ISC process, k_{ISC}, as well as radiative decay from triplet state, k_P, thus increasing the quantum yield of phosphorescence, $\Phi_{Phos.}$, and shortening its lifetime $\tau_{Phos.}$, simultaneously (Supplementary Table 4). The TRES mapping showed a red-shift from the fluorescence of PY, F_G, to complex emission, L_C, occurred in the range of 40 ns, and then transformed to phosphorescence of PY, P_G, in the millisecond range (Supplementary Fig. 46). This indicated the photophysical mechanism in Fig. 3f was also applicable to PY−BrCzA system.

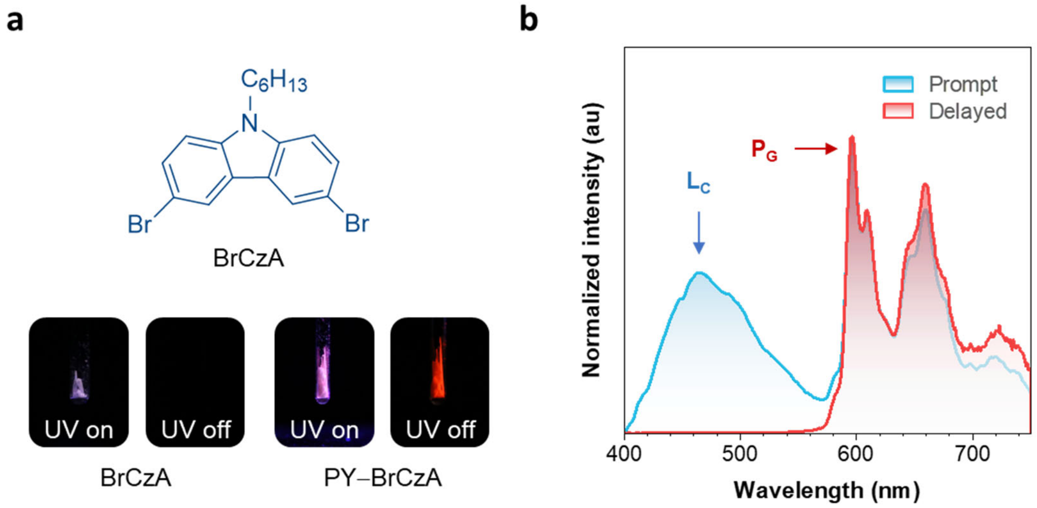

Supplementary Figure 45 | (a) Photographs of BrCzA, PY−BrCzA crystals taken with the 365 nm UV lamp turned on and off. (b) The prompt and delayed PL spectra of PY−BrCzA systems excited at 365 nm. Delay time: 10 ms. The emission of complex L_C, and guest phosphorescence, P_G, were noted.

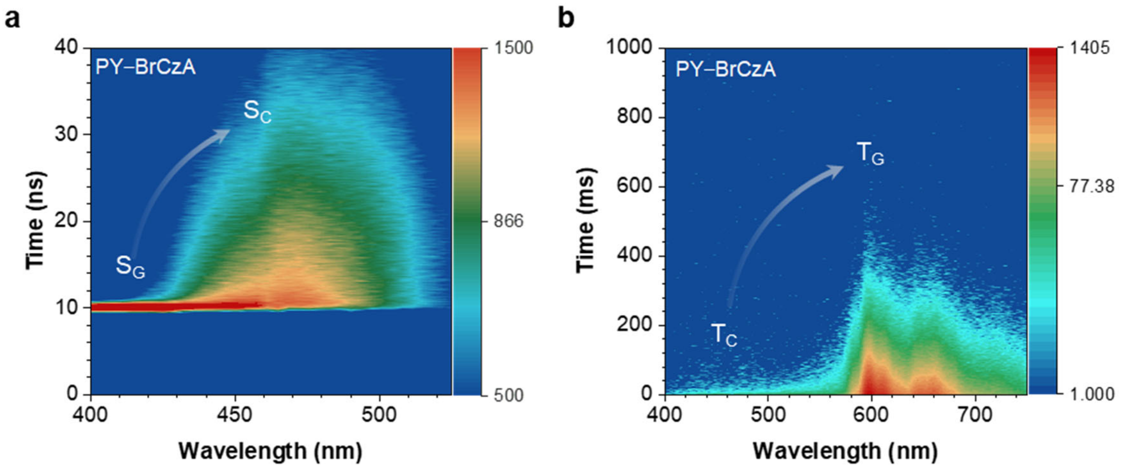

Supplementary Figure 46 | TRES mapping of PY−BrCzA crystals in the range of 40 ns (a) and 1000 ms (b).

Pressure-dependent experiments

As shown in Supplementary Fig. 47a, all diffraction peaks shifted toward higher angles with increasing pressure, suggesting an obvious reduction in the unit cell volume and interplanar spacing. The diffraction peaks became wider, and the intensity kept decreasing as pressure increased, suggesting a gradual amorphization process. Taking (004) crystal plane as an example, we calculated its interplanar spacing, which showed a continuous decreasing from 4.56 Å to 4.33 Å (Supplementary Fig. 47b). Since (004) crystal plane was perpendicular to *c* axis, the compressed rate in the direction of *c* axis could be quantified accordingly. Supplementary Fig. 47c showed that the molecular packing of BrCzA exhibited a herringbone arrangement in the crystal structure of PY−BrCzA system and Supplementary Fig. 47d illustrated how the crystal was compressed in the direction of *c* axis. Thus, no matter if the doped PY molecule replaced BrCzA molecule at its original position or filled in the space between BrCzA molecules, the intermolecular distance between PY and BrCzA in the complex decreased under pressure due to a cell volume shrinkage.

The smaller distance between PY and BrCzA would increase the overlap between their molecular orbitals, leading to stronger intermolecular charge transfer (CT) interactions. This was further supported by spectral evidence showing that the complex emission L_C exhibited a gradual red-shift and weakened intensity with increasing pressure (Supplementary Figs. 48a, 48b and 49). Surprisingly, the trend of weakening intensity of L_C was highly consistent with the trend of reduced interplanar spacing, further confirming the process of decreased intermolecular spacing and strengthened intermolecular coupling under high pressure (Supplementary Fig. 48c).

Furthermore, increasing pressure typically restricted molecular motion and suppressed non-radiative decay, thereby enhancing phosphorescence. However, in our system, despite the suppression of non-radiative transitions, phosphorescence gradually weakened and eventually quenched (Supplementary Fig. 48d). This was because the coupling interactions between host and guest molecules were strengthened with increased pressure. Additionally, the high pressure restricted molecular motion, hindering the dissociation of complex during dynamic coupling process. This made it difficult for excitons to reach T_G state, ultimately leading to the disappearance of phosphorescence. Therefore, pressure-dependent experiments provided compelling evidence for dynamic coupling-induced phosphorescence through *reductio ad absurdum*.

Additional information for pressure-dependent experiments

Due to the limitations of our pressure-dependent PL spectrometer, which could only measure prompt spectra and could not capture delayed spectra, we needed to enhance the intensity of P_G signal in the prompt PL spectra to observe its relationship with L_C as pressure increased. This necessitated the use of PY−BrCzA system instead of the CzA system, of which the P_G signal was so weak that it was hidden by L_C emission.

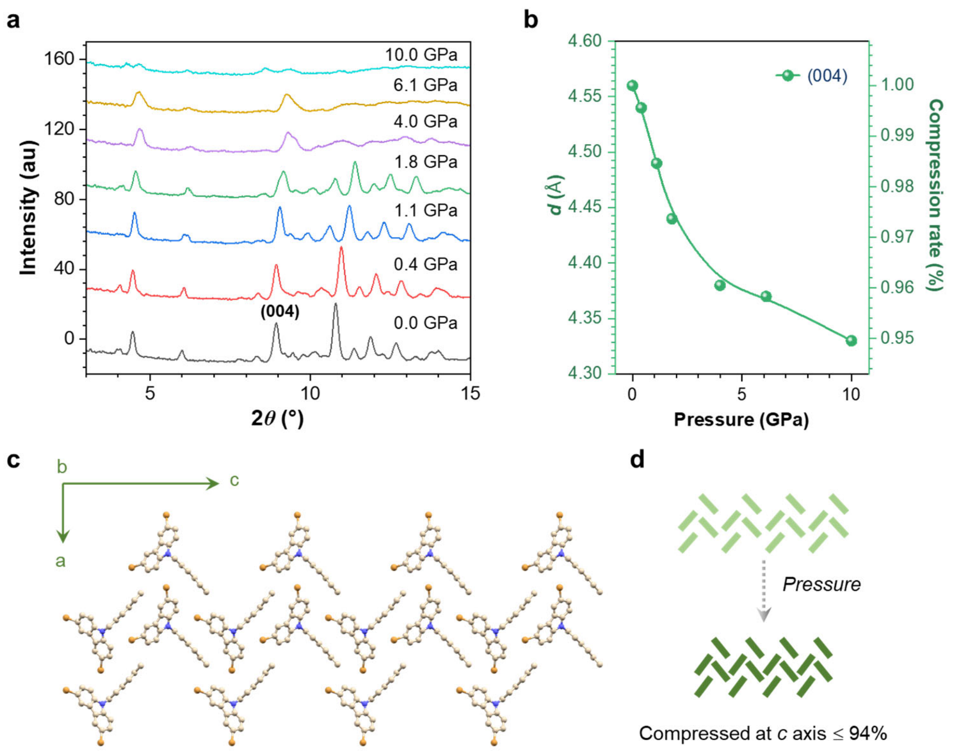

Supplementary Figure 47 | Structural evolution of PY−BrCzA system under high pressure. (a) *In situ* pressure-dependent X-ray diffraction patterns of PY−BrCzA crystals. (b) Interplanar spacing of (004) crystal plane under different pressure. The right axis indicated the compression rate in the direction of *c* axis. (c) Molecular packing of BrCzA in the crystal structure. (d) Schematic diagram of compressed crystals under high pressure.

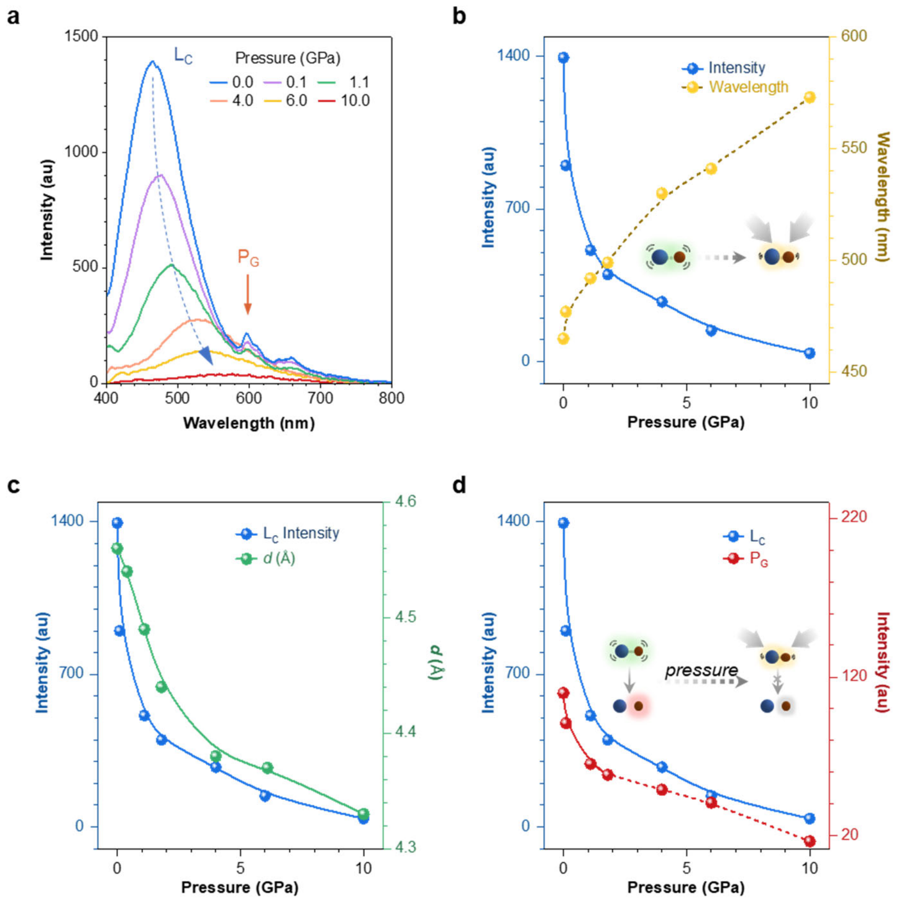

Supplementary Figure 48 | (a) *In situ* pressure-dependent PL spectra of PY−BrCzA crystals. (b) Variation of L_C in intensity and wavelength. Inset illustrated that with increasing pressure, the coupling between host and guest was strengthened. (c) Variation of L_C intensity and interplanar spacing of (004) crystal plane as external pressure increased. (d) The intensity variations of L_C and P_G as external pressure increased. Inset illustrated that as the decoupling process was suppressed, the phosphorescence was weakened and eventually quenched.

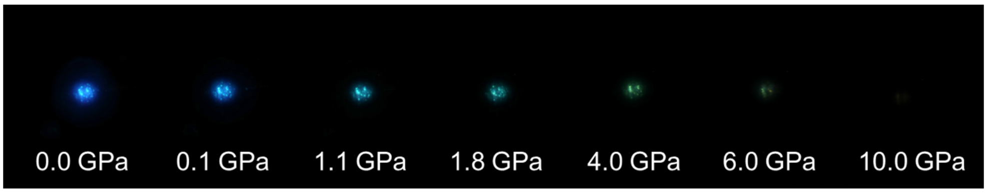

Supplementary Figure 49 | Photographs of PY−BrCzA crystals taken under different pressure while being exposed to 355 nm UV light.

Quantitative modulation of coupling strength: proximity effect

Fig. 4 demonstrates whether RTP is turned on depends on whether the coupling complex can undergo a decoupling process, enabling the excitons to transition from the T_C state to the triplet state of guest, T_G. In the T_C state, electrons are delocalized across the entire complex, exhibiting CT characteristics, whereas in the T_G state, electrons are localized on the guest molecule, displaying locally excited (LE) characteristics. Thus, the decoupling process (i.e. T_C to T_G transition) inherently involves an electron exchange process to revert the CT state to the LE state. According to the Arrhenius equation (Eq. S1), the rate constant of this electron exchange process, k_{ex}, is governed by the activation energy barrier of decoupling process, $\Delta G_{dc}^{\neq}$.

$$k_{ex} = k_0 \exp\left(-\frac{\Delta G_{dc}^{\neq}}{RT}\right) \quad \text{Eq. S1}$$

k_{ex}, rate constant of electron exchange during decoupling process; k_0, pre-exponential factor, representing the maximum k_{ex} when $\Delta G_{dc}^{\neq}$ is 0 kcal/mol. $\Delta G_{dc}^{\neq}$ is the activation energy for decoupling process.

For a certain host–guest system, since the complex is formed through coupling between partially charged donor and acceptor moieties, whose relative motion is constrained by Coulombic interactions. In this context, the activation energy required for decoupling corresponds to the energy barrier needed to overcome the Coulombic potential. According to Coulomb's law, this interaction energy (U) can be quantitatively described by Eq. S2.

$$U = \frac{1}{4\pi\varepsilon_0\varepsilon_r} \cdot \frac{q_1 q_2}{r} \quad \text{Eq. S2}$$

ε_0 is the vacuum permittivity and ε_r is the relative permittivity. q_1 and q_2 represent the charges carried by the host and guest respectively. Since the host and guest are fixed in one identical system, their electron-donating/accepting capabilities remain constant, which means the quantities of partial charges they carried are fixed. Therefore, all these terms, ε_0, ε_r, q_1, and q_2 can be grouped into constant factors. r represents the intermolecular distance. Since the volume V of a solid scales with r^3 ($V \propto r^3$), and volume is inversely proportional to pressure p ($V \propto 1/p$), we can establish the relationship between U and p as shown in Eqs. S3–S4. Eq. S4 demonstrates that by varying external pressure, we can control the intermolecular distance and thereby modulate the activation energy for decoupling ($\Delta G_{dc}^{\neq}$).

$$\frac{1}{r} \propto p^{1/3} \quad \text{Eq. S3}$$

$$\Delta G_{dc}^{\neq} = U = k_C \cdot p^{1/3} \quad \text{Eq. S4}$$

where k_c is a constant term determined by the intrinsic electronic properties of host and guest molecules, as well as the compressibility of the host crystals.

We take PY–BrCzA as an example and applied external pressure in the range of 0-10 GPa to modulate the activation energy by compressing intermolecular distances. Figure 4d shows its PXRD patterns under different pressure. All diffraction peaks shift toward higher angles with increasing pressure, indicating significant reduction in unit cell volume and interplanar spacing. Under 0-6 GPa external pressure, the main crystal diffraction peaks remain observable, demonstrating its crystal structure remain intact up to 6 GPa. At 10 GPa, the diffraction peaks weaken to near undetectable levels, indicating transition from crystalline to amorphous state. Thus, we can confirm that within the 0-6 GPa range, the proportional relationship between $1/r$ and $p^{1/3}$ in Eq. S3 is still valid, and consequently the correlation between $\Delta G_{dc}^{\neq}$ and $p^{1/3}$ in Eq. S4 remains effective.

Due to technical limitations in measuring pressure-dependent phosphorescence lifetimes, we utilize the phosphorescence intensity (I_P) as the performance metric. According to Eq. S1, we can derive an exponential relationship between I_P and $\Delta G_{dc}^{\neq}$.

$$I_P \propto \exp\left(-\Delta G_{dc}^{\neq}\right)$$

Combining with Eq. S4, a negative linear correlation should exist between ln(I_P) and $p^{1/3}$.

$$\ln(I_P) \propto -p^{1/3}$$

As demonstrated by the experimental data, Supplementary Fig. 50 shows an exceptionally strong linear correlation (**R^2 = 0.99**) between $\ln(I_P)$ and $p^{1/3}$. Given the established relationship between $p^{1/3}$ and $\Delta G_{dc}^{\neq}$ in Eq. S4, this equivalently confirms a negative linear dependence of RTP intensity on the activation energy for decoupling ($\Delta G_{dc}^{\neq}$). These results provide compelling experimental validation for our proposed mechanism: increasing external pressure elevates the decoupling activation energy through enhanced Coulombic interactions, thereby diminishing the RTP performance.

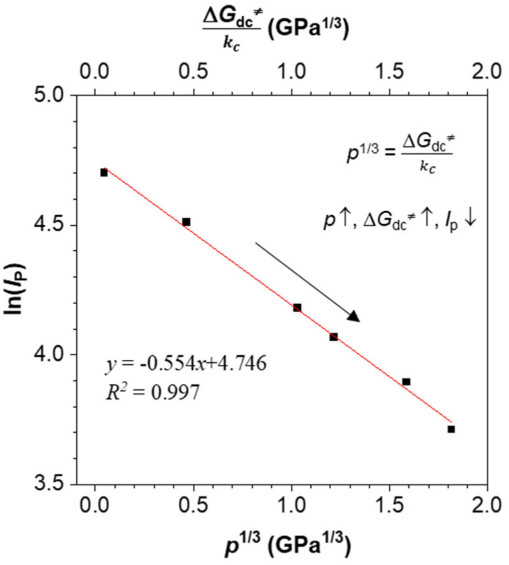

Supplementary Figure 50 | Relationship between phosphorescence intensity I_P and the activation energy for decoupling process, $\Delta G_{dc}^{\neq}$, in PY−BrCzA system under different pressure.

UV-Vis absorption spectra

Supplementary Figure 51 demonstrates that the absorption spectra of PY−BrCzA crystals exhibit a progressive bathochromic shift in the onset position from 398 nm to 475 nm under increasing pressure. This observation clearly indicates that the electronic coupling between PY and BrCzA is enhanced through compressed intermolecular distance, thereby gradually lowering the energy gap of CT complex state. These spectral changes correlate well with the redshift of complex emission (L_C) observed in Fig. 4c. The parallel bathochromic trends in both absorption and emission spectra unambiguously provide evidence for the enhanced CT interactions between PY and BrCzA. Upon releasing the pressure back to ambient conditions, the absorption spectrum fully recovered to its original state, suggesting that this pressure-enhanced coupling process is reversible.

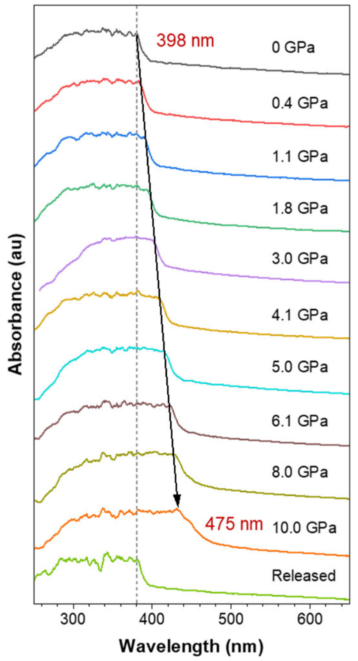

Supplementary Figure 51 | UV-Vis absorption spectra of PY−BrCzA under different pressure.

Time-resolved luminescence decay curves

To further investigate the evolution of complex emission (L_C), we measure its lifetime under varying pressure conditions. As the external pressure increases from 0 GPa to 10 GPa, the lifetime of L_C exhibits a significant decrease from 33 ns to 3 ns (Supplementary Fig. 52 and Supplementary Table 5). Detailed analysis of the radiative (k_r) and non-radiative (k_{nr}) decay rate constants (Fig. 4f) reveals a consistent trend: k_r progressively decreases while k_{nr} keeps increasing with pressure.

This phenomenon can be attributed to two primary factors: First, the enhanced CT character reduces the orbital overlap between HOMO and LUMO, leading to a more forbidden transition from S_C state (singlet state of coupling complex), thereby decreasing k_r. Second, the reduction in intermolecular distance between PY and BrCzA under pressure promotes molecular collisions and facilitates nonradiative energy transfer, resulting in the observed increase in k_{nr}. Thus, these lifetime measurements also strongly support our conclusion that increasing pressure enhances intermolecular coupling.

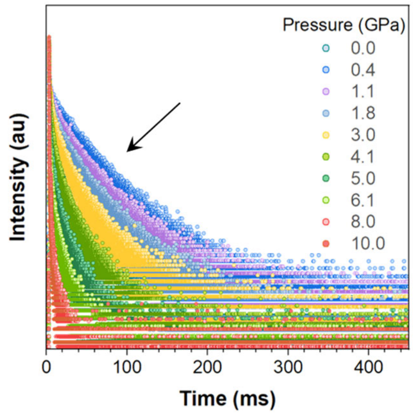

Supplementary Figure 52 | Lifetime curves of PY−BrCzA recorded at 500 nm under different pressure.

FT-IR spectra and Raman spectra

In situ FT-IR and Raman spectra under high pressure were also measured (Supplementary Figs. 53–55). The single-crystal structure of BrCzA reveals distinct C–H···Br interaction (Supplementary Fig. 53), which can be used as a sensitive indicator for monitoring the evolution of intermolecular interactions under pressure.

As illustrated in Supplementary Fig. 54, the majority of FT-IR absorption peaks exhibit blue shifts with increasing pressure, consistent with bond shortening induced by lattice compression. Some typical absorption bands are assigned. For example, the band at ~1280 cm^{-1} corresponds to in-plane ring-stretching vibrations of the benzene moiety. Its progressive splitting and broadening under pressure directly reflect enhanced π–π stacking interactions. Characteristic stretching modes of C–H and C–Br bonds (annotated in Supplementary Fig. 53) display contrasting spectral trends: a continuous blue shift for ν(C–H) from 2931 to 2983 cm^{-1}, versus a red shift for ν(C–Br) from 734 to 723 cm^{-1}. This provides compelling evidence for the strengthening of C–H···Br interactions under high pressure.

Raman spectral evolution corroborates these findings (Supplementary Fig. 55). The C–H bending mode shows a monotonic blue shift, whereas the C–Br bending mode undergoes an initial red shift followed by a blue shift at higher pressures. This might be attributed to the intensified intermolecular interaction dominates at lower pressures, while the bond compression prevails at higher pressure.

Thus, the combined evidence from FT-IR spectra, Raman spectra, complemented by the PXRD analysis, conclusively confirms three key structural changes within PY–BrCzA under high pressure: lattice contraction, shortened intermolecular spacing and amplified intermolecular interactions. These phenomena provide corroborative support for the reinforcement of coupling interaction within the system.

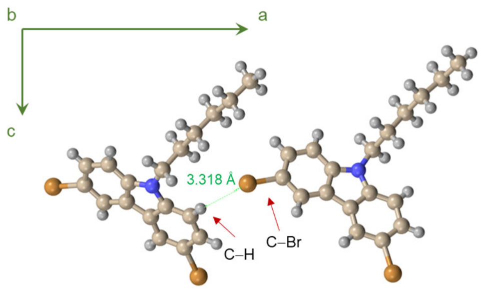

Supplementary Figure 53 | C–H···Br interaction in BrCzA crystal.

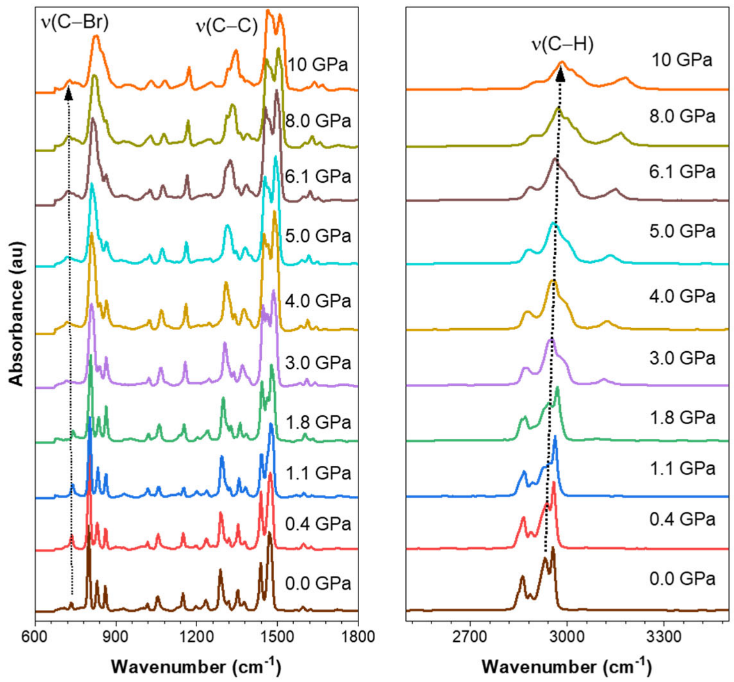

Supplementary Figure 54 | FT-IR spectra of PY−BrCzA under different pressure.

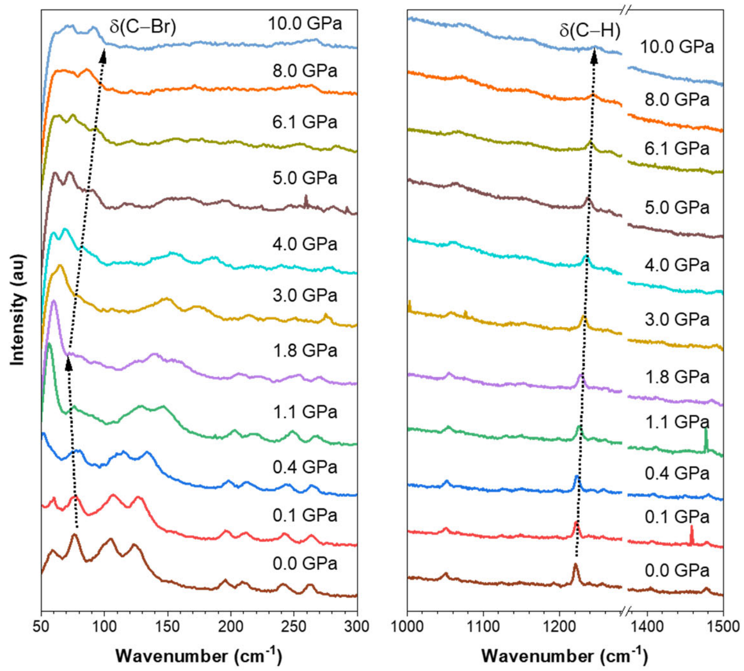

Supplementary Figure 55 | Raman spectra of PY−BrCzA under different pressure.

V. Universal applicability

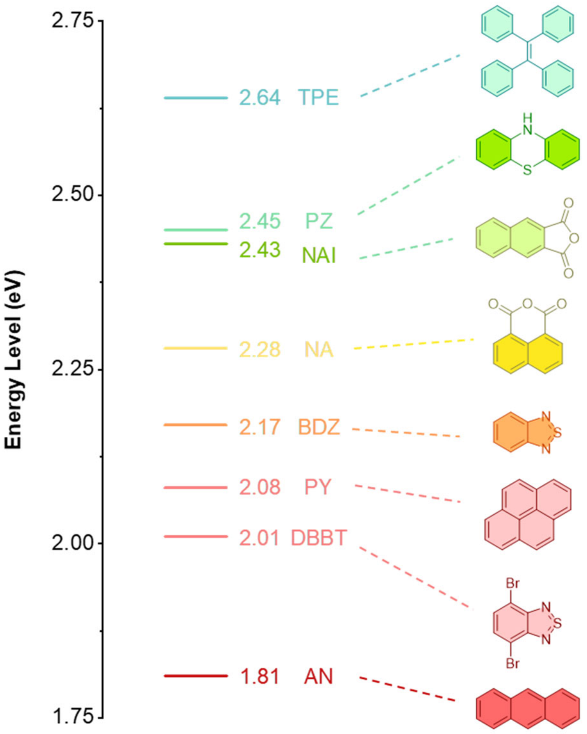

Supplementary Figure 56 | Energy levels of T_1 state of guest molecules used in the investigation of universal applicability. The values were obtained from their phosphorescence spectra.

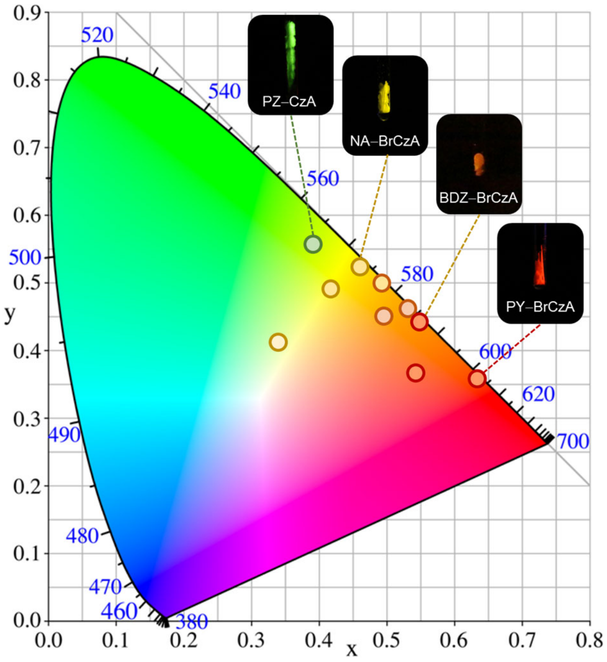

Supplementary Figure 57 | CIE coordinate diagram of host–guest RTP systems. CIE coordinates: PZ–CzA, (0.39, 0.56); NAI–BrCzA, (0.42, 0.49); NA–BrCzA, (0.46, 0.52); NA–BrFA, (0.34, 0.41); BDZ–BrCzA, (0.55, 0.44); DBBT–CzA, (0.49, 0.45); DBBT–BrCzA, (0.53, 0.46); PY–CzA, (0.54, 0.37); PY–BrCzA, (0.63, 0.36); AN–BrCzA, (0.48, 0.51). The insets showed photographs of 4 systems exhibiting green, yellow, orange and red delayed emission taken after the 365 nm UV lamp was turned off.

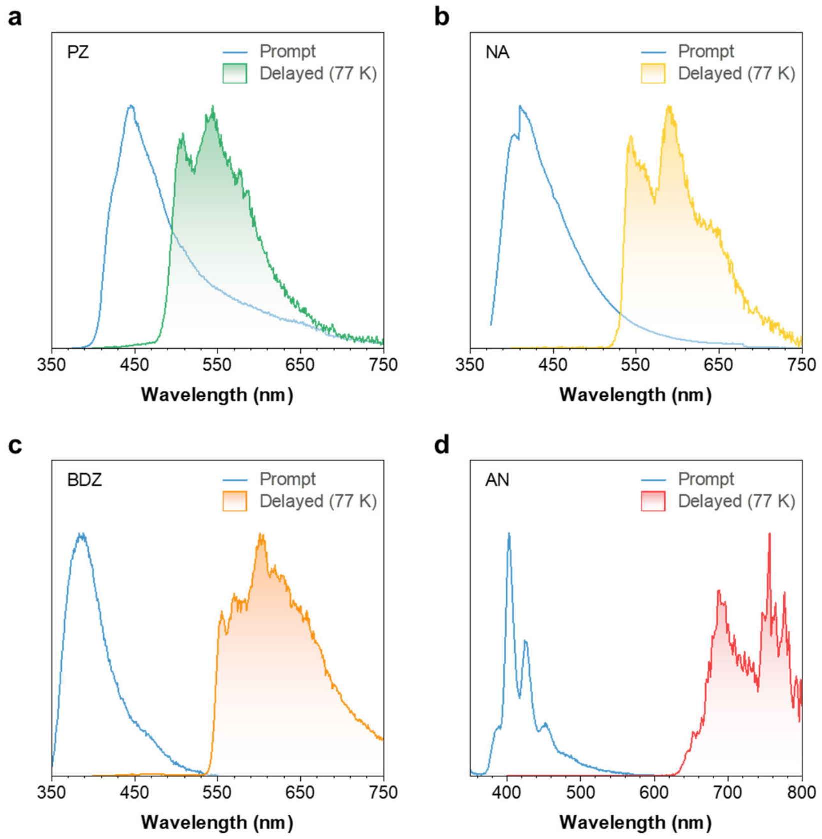

Supplementary Figure 58 | Prompt and delayed PL spectra of guest molecules in PMMA film. The delayed spectra were measured at 77 K. Delay time: 10 ms.

PL spectroscopy study on various host–guest systems

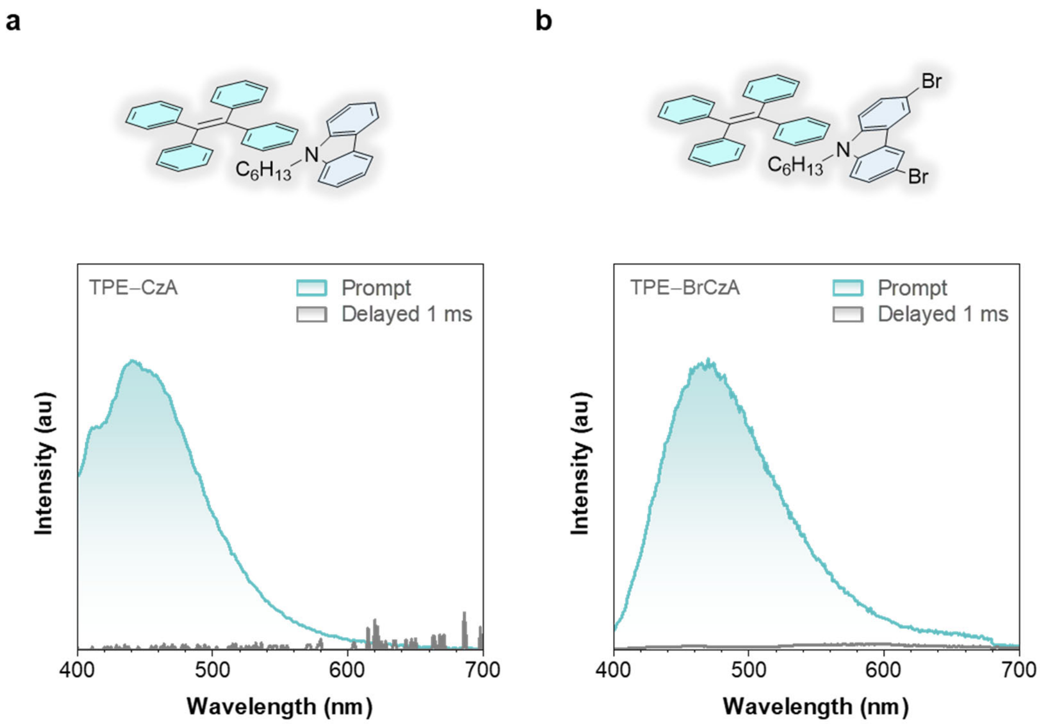

Supplementary Figure 59 | Prompt and delayed PL spectra of TPE–CzA (a) and TPE–BrCzA (b) system. The chemical structures of host and guest molecules were displayed above the spectra. The gray glowing represented there was no coupling occurred.

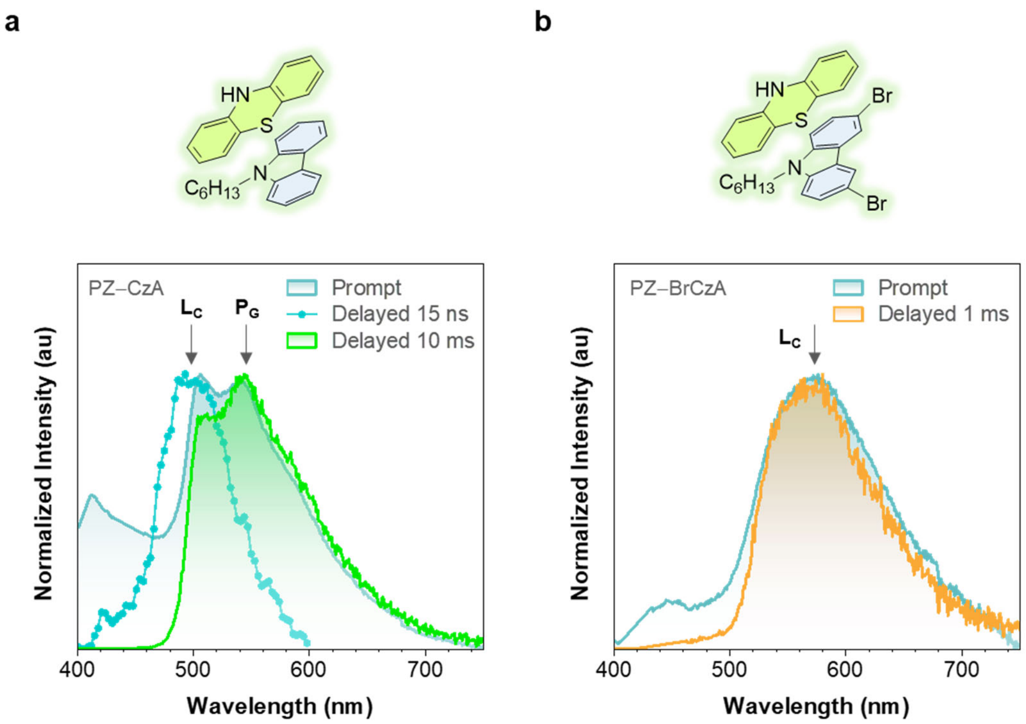

Supplementary Figure 60 | Prompt and delayed PL spectra of PZ–CzA (a) and PZ–BrCzA (b) system. The chemical structures of host and guest molecules were displayed above the spectra. The green glowing represented there were complexes formed. Since L_C was hidden by $F_{H/G}$ and P_G in the prompt PL spectrum of PZ–CzA system, TRES mapping was conducted to separate the spectrum of L_C at 15 ns as shown in Supplementary Fig. 61.

In PZ–CzA system, both L_C and P_G were simultaneously found, supporting the importance of dynamic coupling. However, in PZ–BrCzA system, only L_C was observed. This is because the complex possessed a lower energy level of T_C than that of T_G, making the ET process to T_G energetically unfavored. Thus, the excitons were trapped within the complex and decayed from T_C.

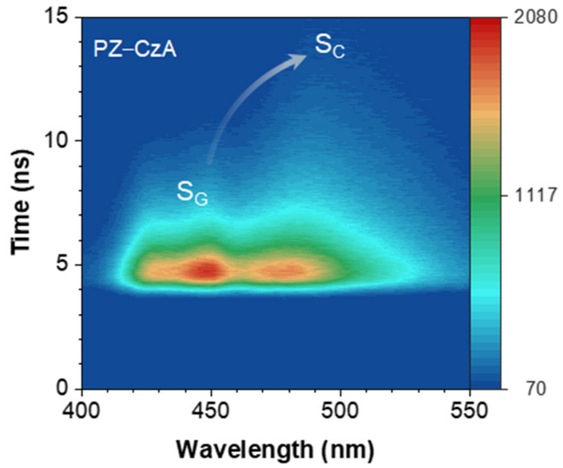

Supplementary Figure 61 | TRES mapping of PZ–CzA crystals within the range of 15 ns. The spectra gradually red-shifted from fluorescence of PZ, F_G, to complex emission, L_C.

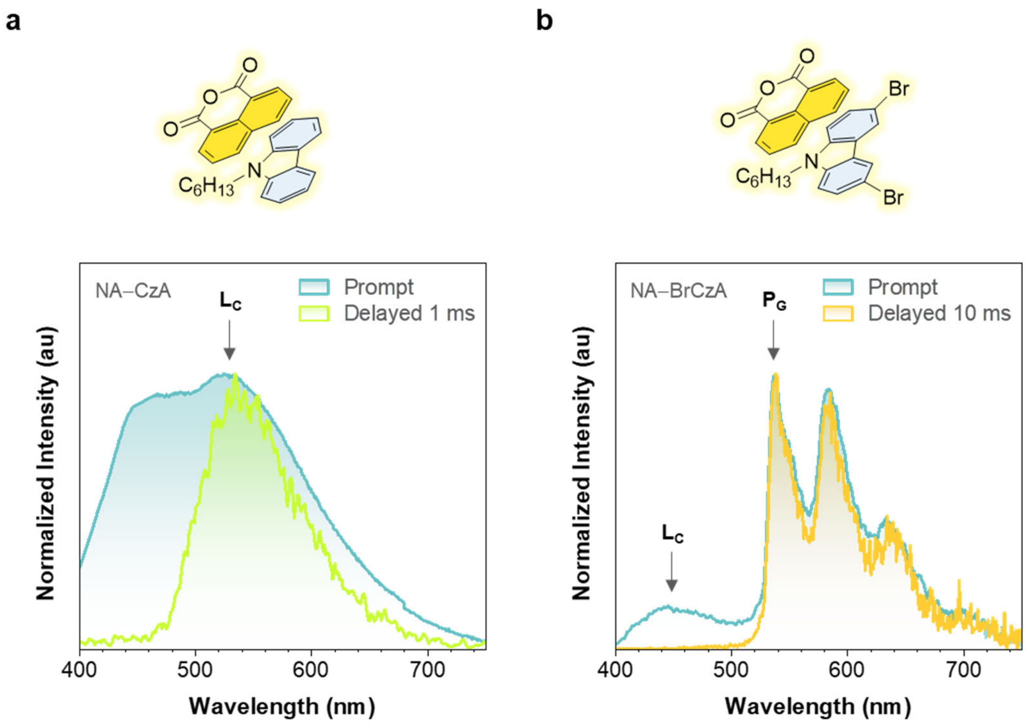

Supplementary Figure 62 | Prompt and delayed PL spectra of NA–CzA (a) and NA–BrCzA (b) system. The chemical structures of host and guest molecules were displayed above the spectra. The yellow glowing represented there were complexes formed.

In NA–CzA system, only L_C was observed because the complex possessed a lower energy level of T_C than that of T_G, making the ET process to T_G energetically unfavored. Thus, the excitons were trapped within the complex and decayed from T_C. However, in NA–BrCzA system, both L_C and P_G were simultaneously found, supporting the importance of dynamic coupling.

To confirm the peak at 450 nm was L_C in NA–BrCzA system, TRES mapping was conducted in Supplementary Fig. 63.

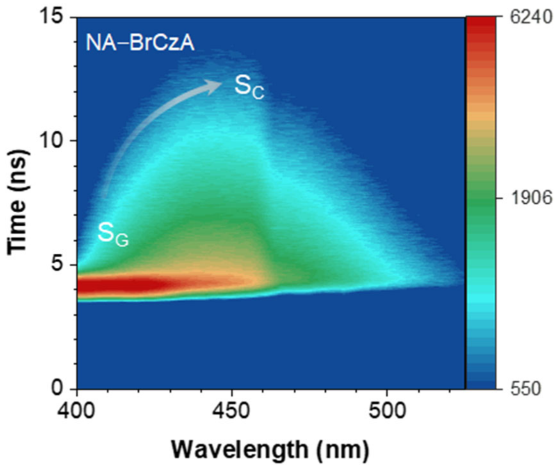

Supplementary Figure 63 | TRES mapping of NA−CzA crystals within the range of 15 ns. The spectra gradually red-shifted from fluorescence of NA, F_G, to complex emission, L_C.

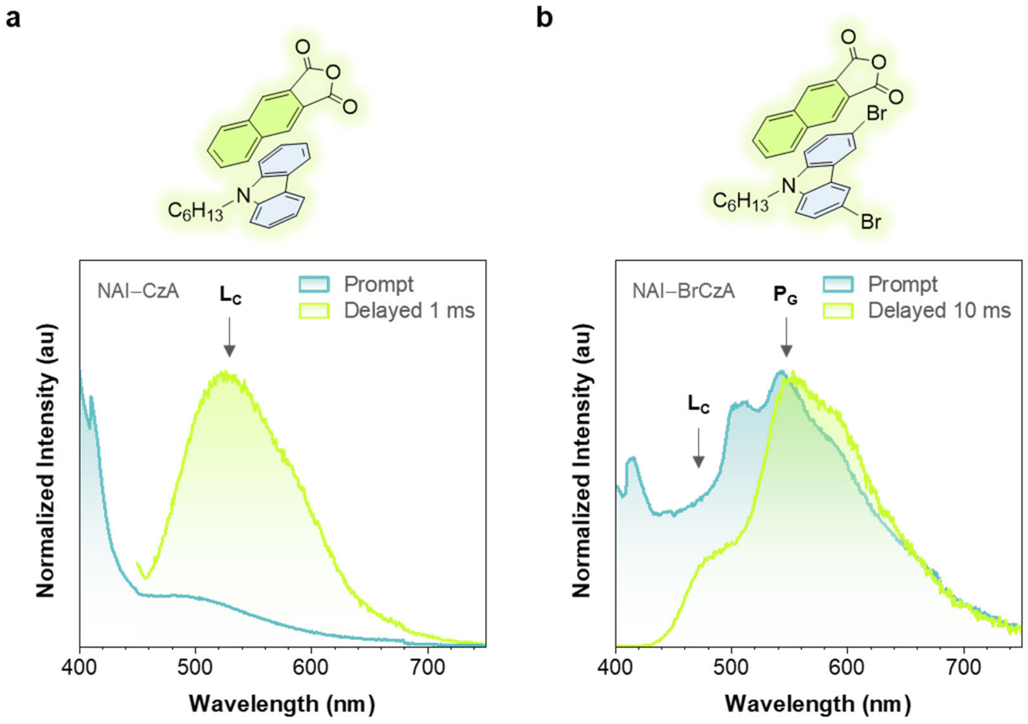

Supplementary Figure 64 | Prompt and delayed PL spectra of NAI–CzA (a) and NAI–BrCzA (b) system. The chemical structures of host and guest molecules were displayed above the spectra. The green glowing represented there were complexes formed.

In NAI–CzA/BrCzA systems, the situations were similar to NA systems. Only L_C was observed in NAI–CzA because the energy level of T_C was lower than T_G, thus trapping the excitons within the complex. Both L_C and P_G were simultaneously found in NAI–BrCzA system, supporting the importance of dynamic coupling.

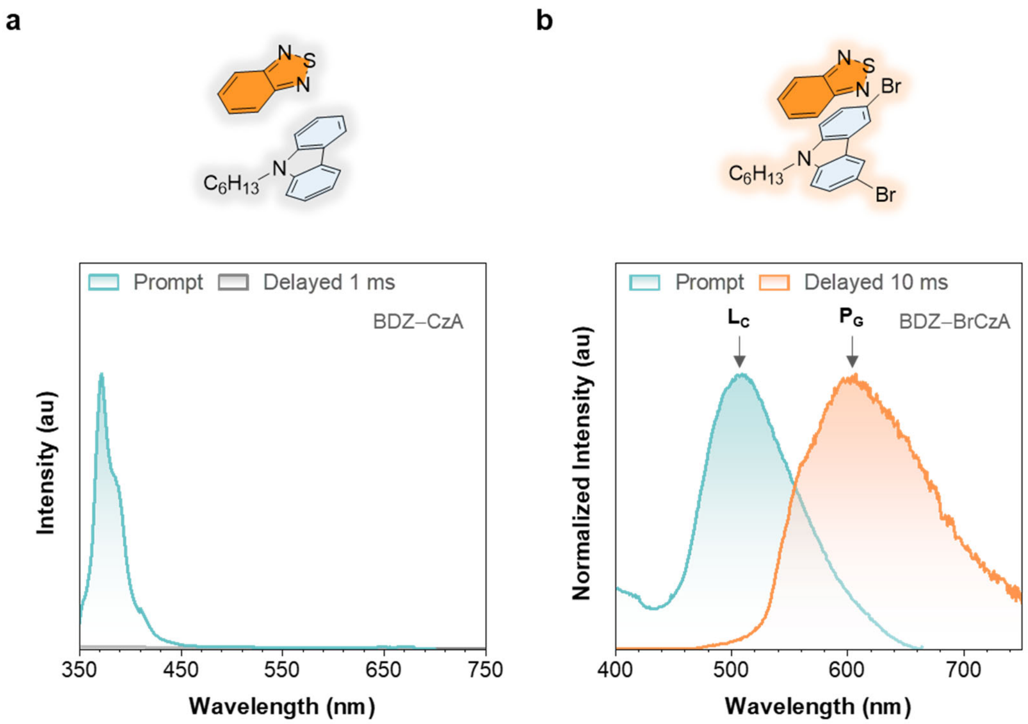

Supplementary Figure 65 | Prompt and delayed PL spectra of BDZ–CzA (a) and BDZ–BrCzA (b) system. The chemical structures of host and guest molecules were displayed above the spectra. The gray glowing represented no complex formed in BDZ–CzA and the orange glowing represented there was complex formed in BDZ–BrCzA.

Neither L_C nor P_G was found in BDZ–CzA system, but they both existed in BDZ–BrCzA system. This indicated the existence of coupling was beneficial for RTP.

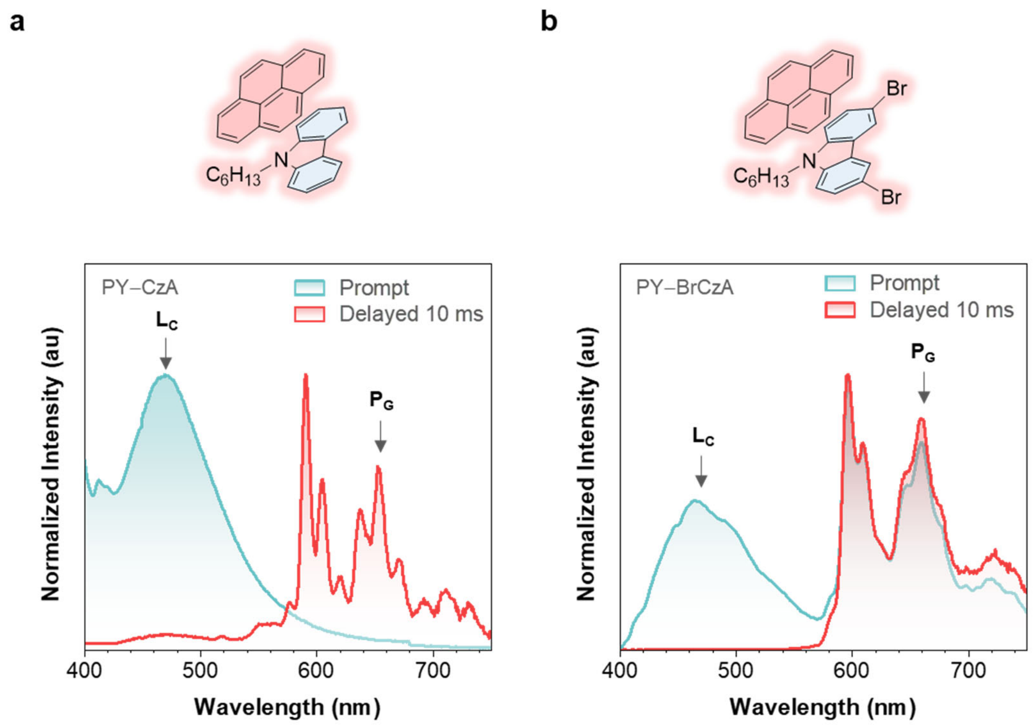

Supplementary Figure 66 | Prompt and delayed PL spectra of PY–CzA (a) and PY–BrCzA (b) system. The chemical structures of host and guest molecules were displayed above the spectra. The red glowing represented there were complexes formed.

Both L_C and P_G existed in PY–CzA/BrCzA systems, making them a good example to discuss dynamic coupling-induced RTP.

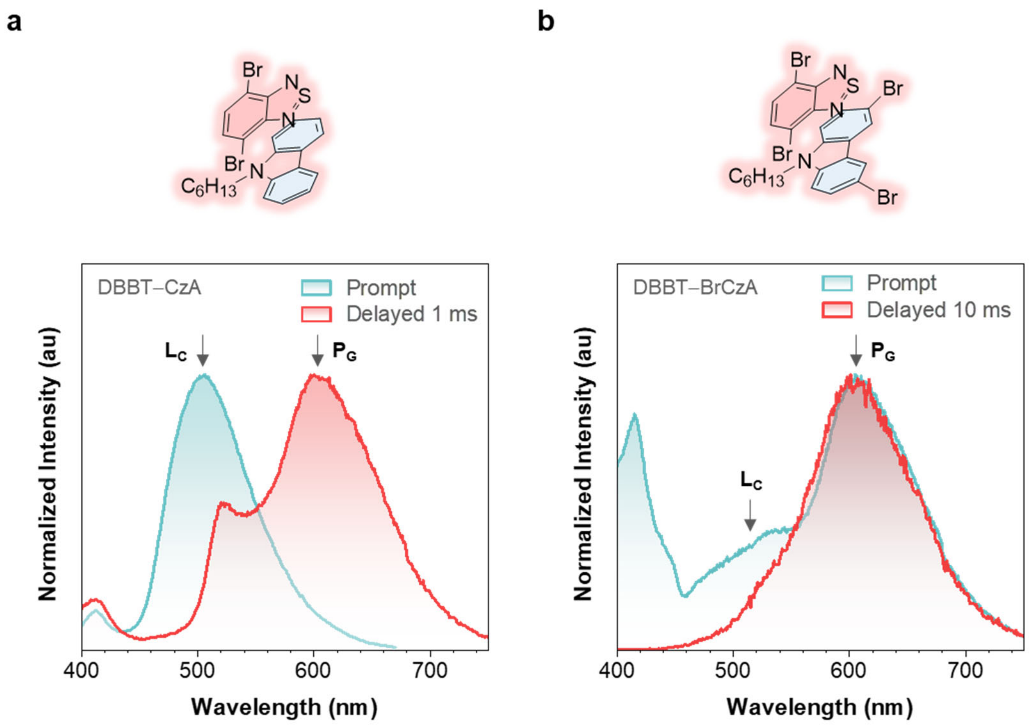

Supplementary Figure 67 | Prompt and delayed PL spectra of DBBT–CzA (a) and DBBT–BrCzA (b) system. The chemical structures of host and guest molecules were displayed above the spectra. The red glowing represented there were complexes formed.

Similar to PY system, both L_C and P_G existed in DBBT–CzA/BrCzA system, supporting the importance of dynamic coupling.

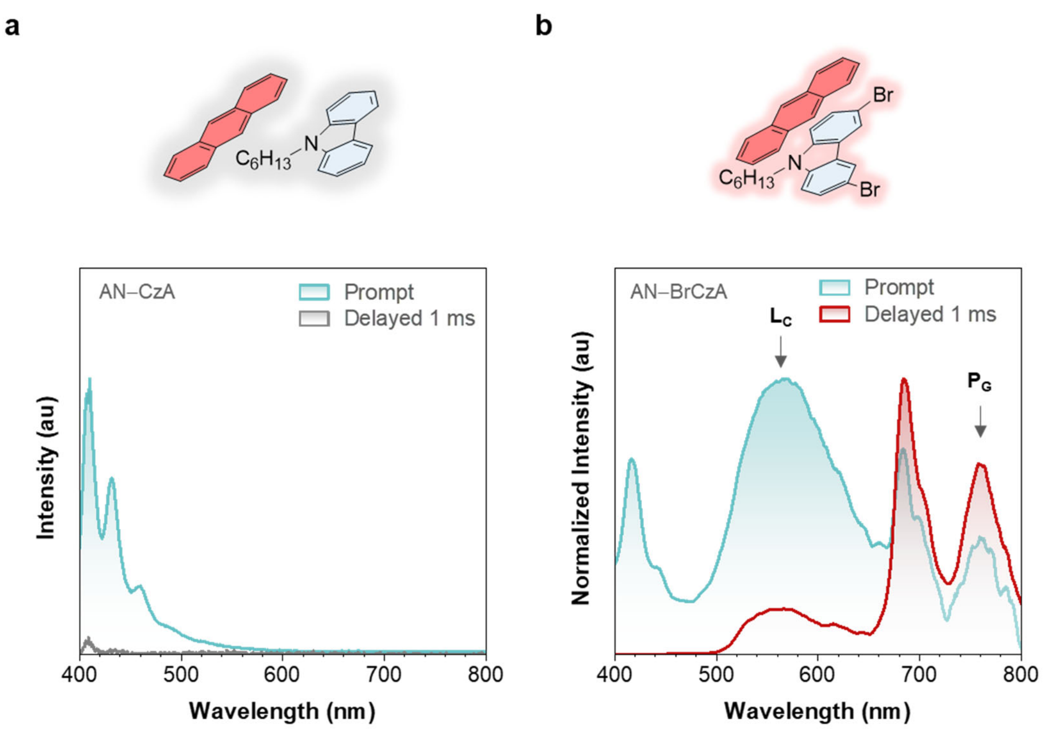

Supplementary Figure 68 | Prompt and delayed PL spectra of AN–CzA (a) and AN–BrCzA (b) system. The chemical structures of host and guest molecules were displayed above the spectra. The gray glowing suggested no complex formed in AN–CzA and the red glowing represented there was complex formed in AN–BrCzA.

Similar to BDZ systems, neither L_C nor P_G was found in AN–CzA system, but they both existed in AN–BrCzA system. The occurrence of coupling in AN–BrCzA system was further confirmed by TRES mapping in Supplementary Fig. 69. This indicated the existence of coupling was beneficial for RTP.

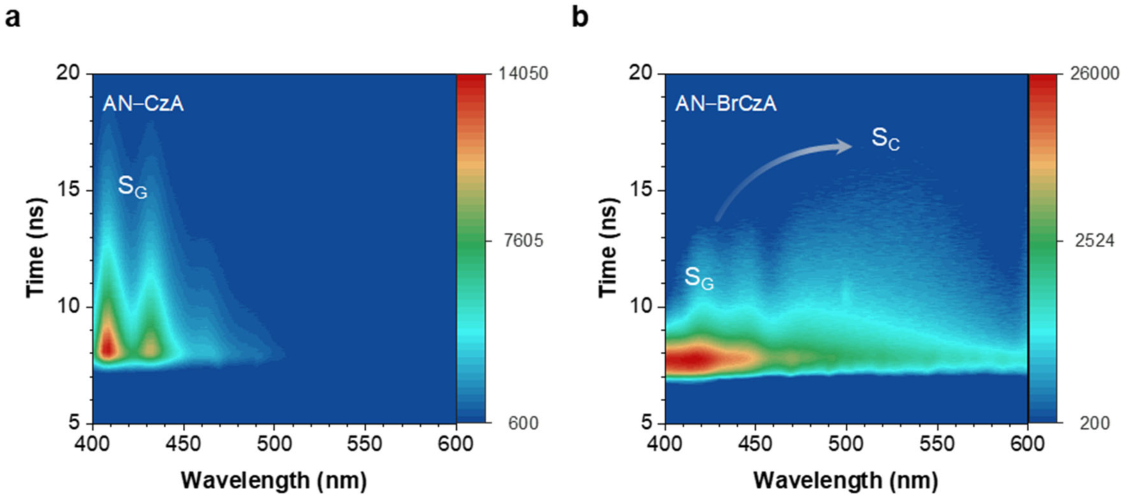

Supplementary Figure 69 | TRES mapping of AN–CzA (a) and AN–BrCzA (b) crystals within the range of 20 ns. In AN–CzA system, there was only fluorescence of AN, F_G, decaying within 20 ns. However, in AN–BrCzA crystals, the spectra gradually red-shifted from F_G to complex emission, L_C, supporting the occurrence of coupling.

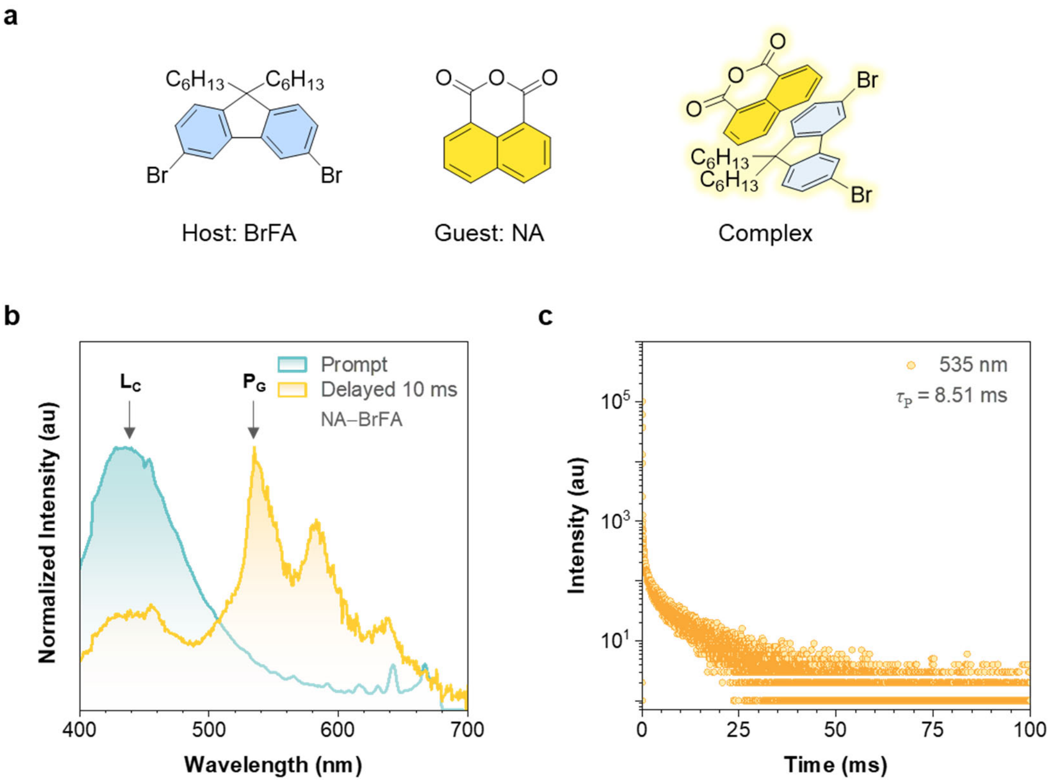

Supplementary Figure 70 | (a) The chemical structures of host, guest molecules and complex in NA–BrFA system. The yellow glowing indicated there was complex formed. (b) Prompt and delayed PL spectra of NA–BrFA system. (c) The lifetime curves of NA–BrFA crystals with emission recorded at 535 nm. The fitted lifetime of phosphorescence was indicated.

To expand the scope of dynamic coupling-induced RTP beyond carbazole derivatives, fluorene derivative, BrFA, was used as host molecules. Both L_C and P_G were found in NA–BrFA system, and the lifetime of P_G was measured as 8.51 ms. This suggested that the complex could also form between fluorene moiety and NA. To further confirm the occurrence of coupling in NA–BrFA system, TRES mapping was conducted in Supplementary Fig. 71.

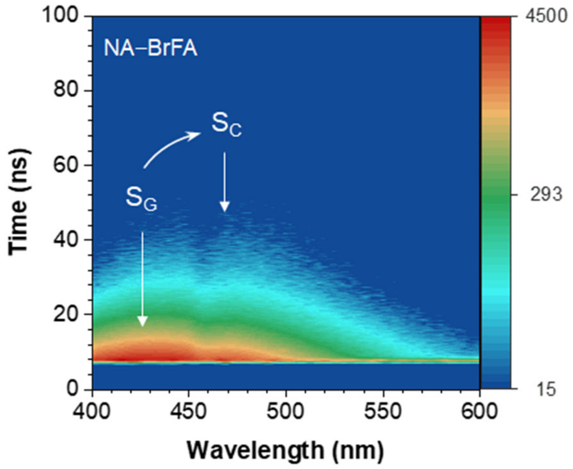

Supplementary Figure 71 | TRES mapping of NA−BrFA crystals within the range of 100 ns. The spectra gradually red-shifted from fluorescence of NA, F_G, to complex emission, L_C, supporting the occurrence of coupling.

Summary of structure-property relationship for host–guest RTP systems

In the first scenario, neither L_C nor P_G was found in these systems, indicating there was no coupling between host and guest molecules to facilitate RTP. The lack of coupling might be attributed to the non-planar structure, which hindered the proximity between the host and guest molecules (TPE), or to the inappropriate intermolecular interactions (BDZ, AN). The ISC processes in host and guest molecules themselves were inefficient. The TTET process from T_H to T_G was also inhibited due to the weak ISC ability of host and the strong non-radiative dissipation from T_H. Thus, no RTP was generated and only $F_{H/G}$ was observed (Supplementary Figs. 72a and 73a).

In the second scenario, L_C appeared in the PL spectra, but P_G was not generated. This indicated coupling occurred but the ET pathway to T_G during decoupling process was inhibited. The reason was that the intermolecular interactions between host and guest molecules were too strong, forming static complexes with T_C lower than T_G. This made the ET process to T_G energetically unfavored. Thus, the excitons were trapped within the static complex and decayed from T_C (Supplementary Figs. 72b and 73b).

In the third scenario, both L_C and P_G were observed in these systems, indicating the excitons were successfully transferred to T_G state by dynamic coupling process. This highlighted the essential role of dynamic coupling in facilitating RTP. In these systems, both the host and guest molecules exhibited planar structures. Furthermore, their electron-donating abilities and accepting tendencies matched well with each other, leading to attractive intermolecular interactions that allowed them to couple effectively without being overly strong. Both factors benefited the formation of dynamic coupling(Supplementary Figs. 72c and 73c).

The above discussion clarified the structure-property relationship for dynamic coupling-induced phosphorescence:

(1) Host and guest molecules should have a certain degree of planarity to enable close proximity to one another.

(2) Their electron-donating and accepting tendencies should align within a specific range to form suitably intermolecular charge transfer interactions without being too strong to trap the excitons.

This structure-property relationship could guide us to design host–guest RTP systems. For example, in systems where excitons were trapped in complex, weakening their interactions could activate phosphorescence (Supplementary Fig. 74a). In PZ–BrCzA, PZ had a strong electron-donating tendency, and BrCzA acted as an electron acceptor. They formed an excessively strong complex that trapped the exciton. By weakening the electron-accepting ability of BrCA by replacing with CzA, dynamic coupling was successfully achieved, thus activating phosphorescence in PZ–CzA (Supplementary Fig. 74b). Conversely, in NA–CzA, NA had a strong electron-accepting tendency, and CzA worked as an electron donor. The intermolecular interaction between CzA and NA was too strong and there was no RTP in NA–CzA. By brominating CzA to weaken its electron-donating ability, BrCzA successfully facilitated dynamic coupling and activated the RTP in NA–BrCzA (Supplementary Fig. 74c).

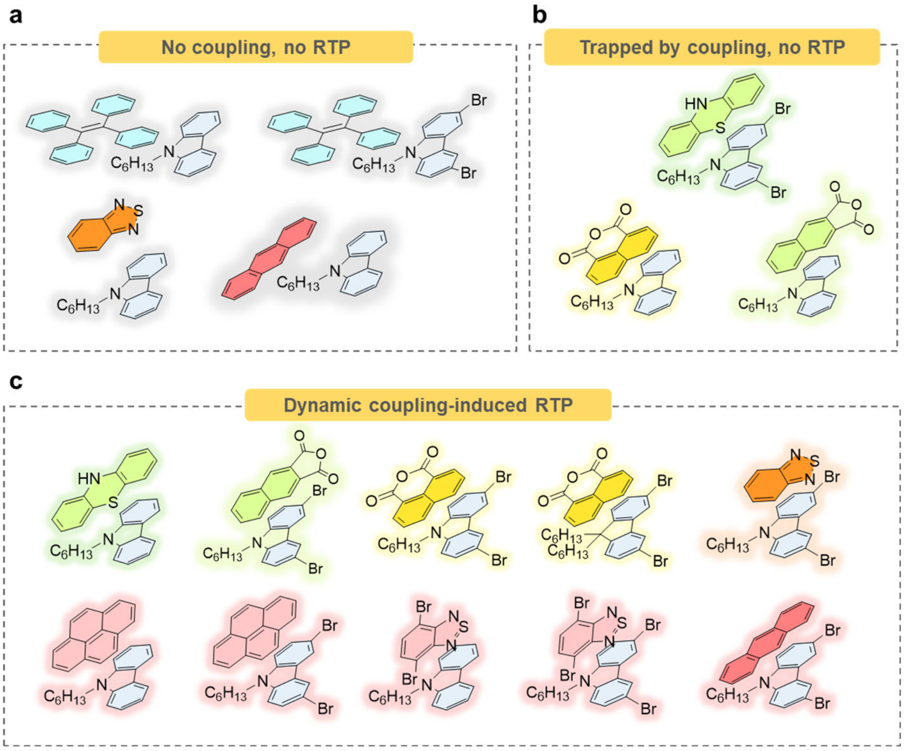

Supplementary Figure 72 | Three scenarios among 17 host–guest systems.

a

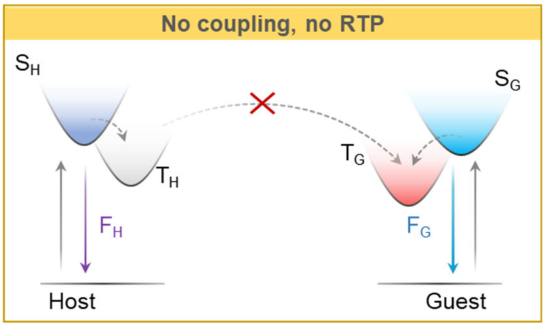

b

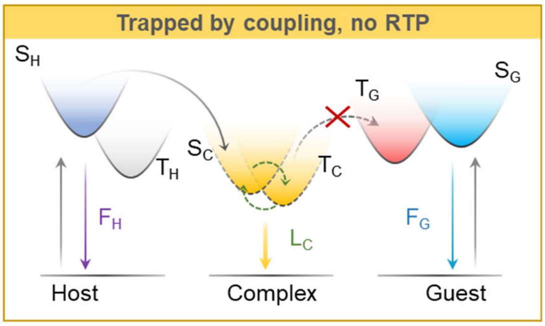

c

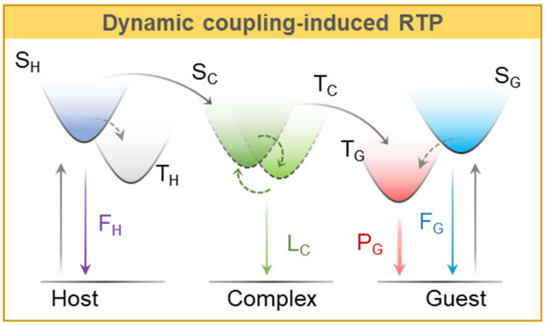

Supplementary Figure 73 | Photophysical processes in above 17 host–guest systems.

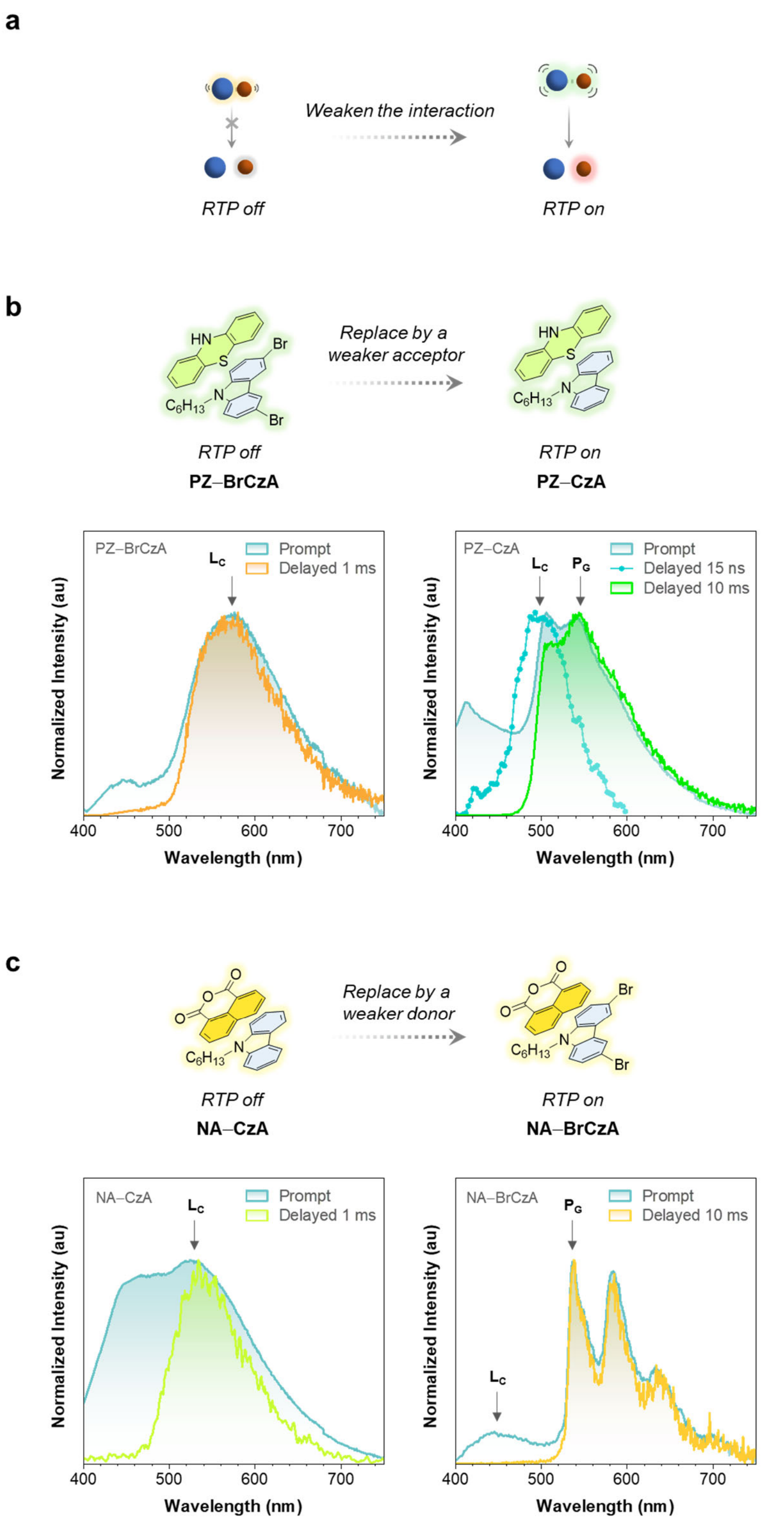

Supplementary Figure 74 | Guiding the design of host–guest RTP systems based on structure-property relationship. (a) Schematic diagram for regulation from static to dynamic coupling. (b) Prompt and delayed PL spectra of NA–CzA and NA–BrCzA system. (c) Prompt and delayed PL spectra of PZ–BrCzA and PZ–CzA system.

VI. Dynamic coupling in PY−CzBP/BPCzA system

Photographs

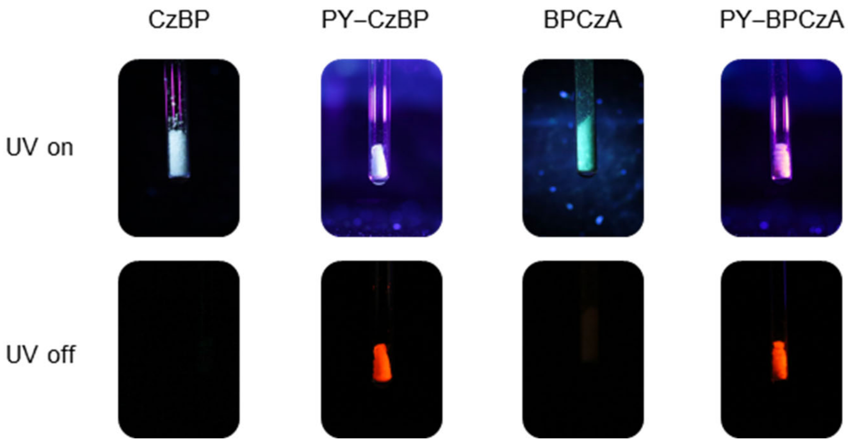

Supplementary Figure 75 | Photographs of CzBP, BPCzA, PY−CzBP, and PY−BPCzA crystals taken with the 365 nm UV lamp turned on and off.

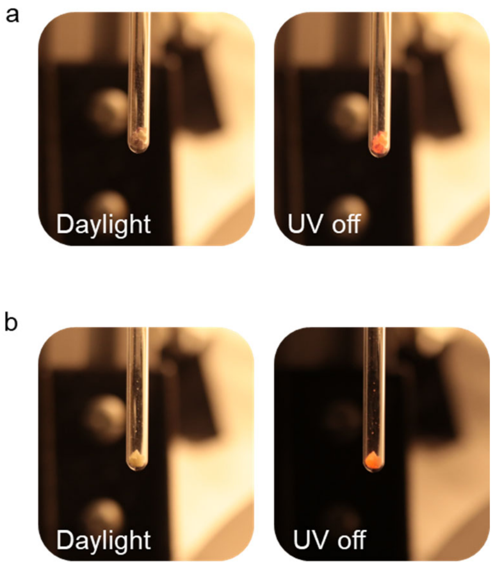

Supplementary Figure 76 | Photographs of PY−CzBP (a), and PY−BPCzA (b) crystals taken under daylight (left) and taken after the 365 nm UV lamp was turned off (right). The red RTP could be seen under ambient light.

Photophysical investigation on dynamic coupling

DFT calculations revealed the complexes in PY−CzBP/BPCzA were coupled by CT interaction. The energy level alignments of FMOs of CzBP/BPCzA and PY matched the requirements for intermolecular CT process (Supplementary Fig. 77a). However, the difference between them and PY−CzA system was that HOMO of PY was higher while LUMO of CzBP/BPCz was lower, indicating PY would serve as the donor and CzBP/BPCzA served as acceptor in these systems. This was also supported by the NTO analysis of S_C states in PY−CzBP and PY−BPCzA systems (Supplementary Fig. 77b).

The TRES mapping in the nanosecond range showed a red-shift from the fluorescence of PY or CzBP/BPCzA, $F_{H/G}$, to complex emission, L_C, and then gradually transformed to phosphorescence of PY, P_G, in the millisecond range (Supplementary Fig. 78). This supported the photophysical mechanism in PY−CzBP/BPCzA systems also involved the complex as a bridge for ET process.

The excitation spectra of PY−CzBP/BPCzA crystals with emission recorded at P_G matched well with that of the dynamic complex component in the range of 250 nm to 425 nm, indicating the excitons on T_G shared the same photophysical pathway with dynamic complex (Supplementary Fig. 79). The dynamic component was obtained by measuring the excitation spectra of CzBP/BPCzA crystals, respectively, since the dynamic complex was formed by coupling of excited-state host molecules and ground-state guest molecule. The excitation-phosphorescence mapping also exhibited a similar pattern with the excitation spectra of CzBP and BPCzA crystals from 300 nm to 400 nm, supporting the excitons on T_G were transferred from the dynamic complex (Supplementary Fig. 80).

UV-Vis absorption spectra showed a newly aroused peak located at around 450 nm (Supplementary Fig. 81), suggesting the formation of static complex in PY−CzBP/BPCzA systems. This suggested the static complex could be excited at 450 nm while dynamic complex would form when excited at 365 nm. As expected, P_G appeared in the delayed PL spectra when PY−CzBP/BPCzA crystals were excited at 365 nm but disappeared when excited at 450 nm, as shown in Supplementary Fig. 82. This supported the necessity of dynamic coupling for facilitating RTP in PY−CzBP/BPCzA systems.

The conformations of PY/CzBP and PY/BPCzA dimers were optimized in the S_0, S_C, T_C, and T_G states (Supplementary Fig. 83 and 84). In PY/CzBP, the overlap ratio increased, and the π–π interaction region expanded from S_0 to S_C state, indicating the coupling process occurred. While from T_C to T_G, the overlap ratio decreased and the NCI region shrank, corresponding to the decoupling process. In PY/BPCzA, the process was similar, except the overlap ratio in S_C decreased. This was due to the strong C−H···O interaction between the pyrene ring and carbonyl group on BPCzA (the blue area in Supplementary Fig. 84). The enhanced C−H···O interaction suggested the coupling process also occurred in the S_C state. And the decreased overlap ratio and shrunk NCI region from T_C to T_G state were consistent with the decoupling process.

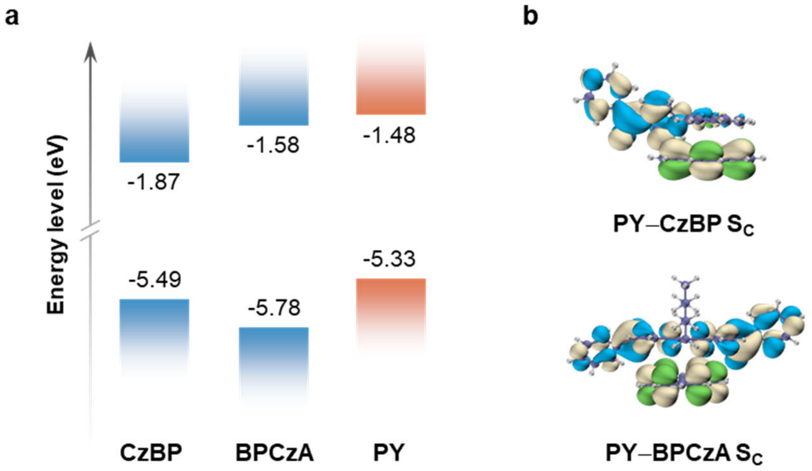

Supplementary Figure 77 | (a) Energy level alignments of frontier molecular orbitals (FMOs) of CzBP, BPCzA and PY. (b) Natural transition orbitals (NTOs) contributing to the transitions for singlet state of complex (S_C) in PY−CzBP and PY−BPCzA systems. Color code: green, hole; blue, particle.

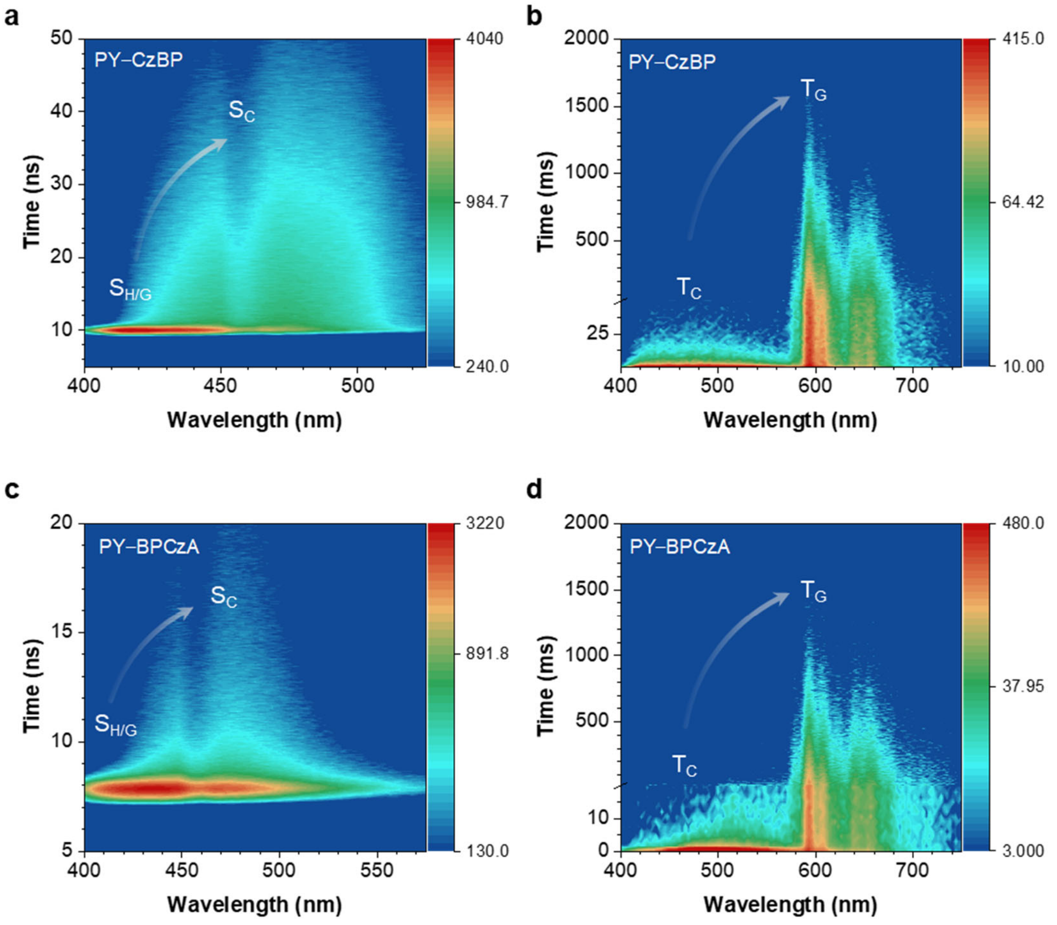

Supplementary Figure 78 | TRES mapping of PY−CzBP (a,b) and PY−BPCzA (c,d) crystals in the nanosecond range (a,c) and millisecond range (b,d).

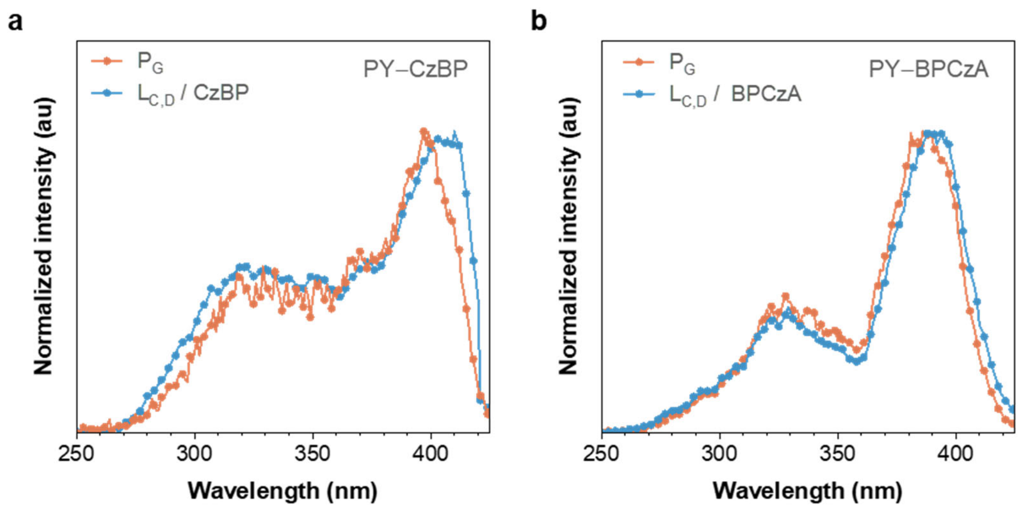

Supplementary Figure 79 | Excitation spectra of PY−CzBP (a) and PY−BPCzA (b) systems, recorded at 595 nm (P_G) with a delay time of 10 ms. Excitation spectra of CzBP and BPCzA crystals recorded at 500 nm were attached as the dynamic complex component, since the dynamic complex was formed by coupling of excited-state host molecules and ground-state guest molecule.

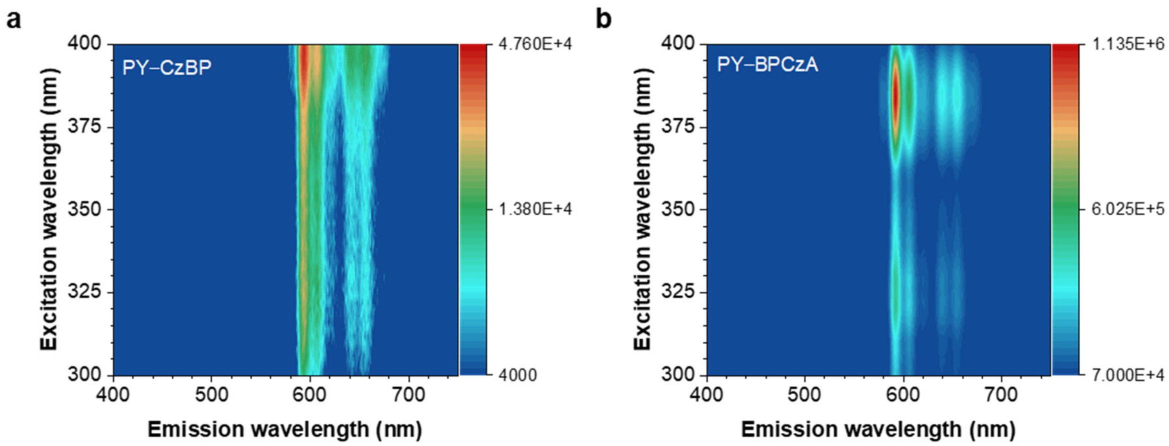

Supplementary Figure 80 | Excitation-phosphorescence mapping of PY−CzBP (a) and PY−BPCzA (b) systems with a delay time of 10 ms.

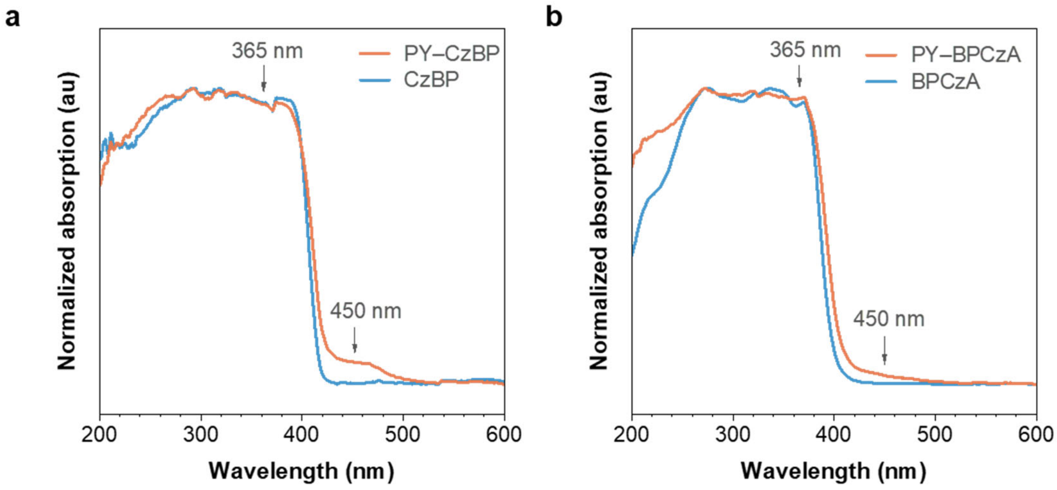

Supplementary Figure 81 | UV-Vis absorption spectra of CzBP, PY−CzBP crystals (a), and BPCzA, PY−BPCzA crystals (b).

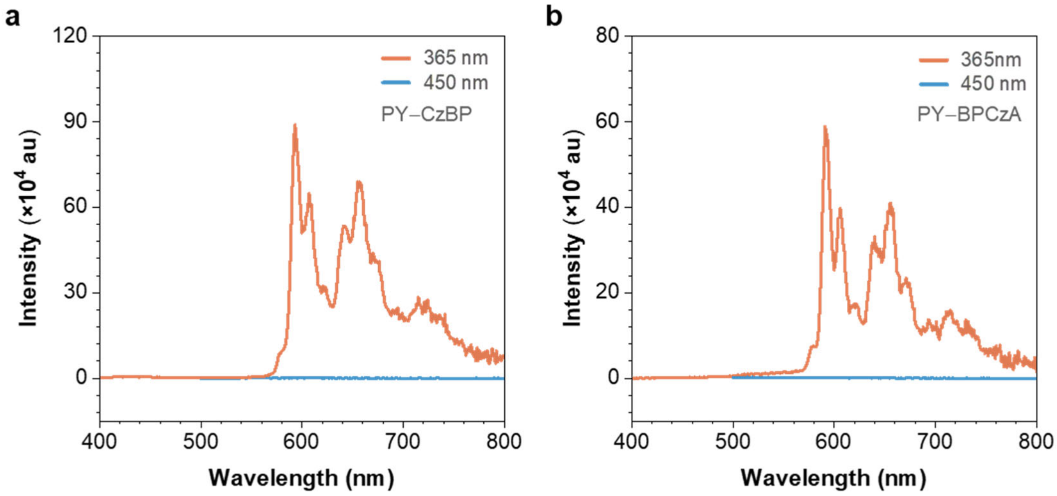

Supplementary Figure 82 | Delayed PL spectra of PY−CzBP crystals (a), and PY−BPCzA crystals (b), excited at 365 nm and 450 nm.

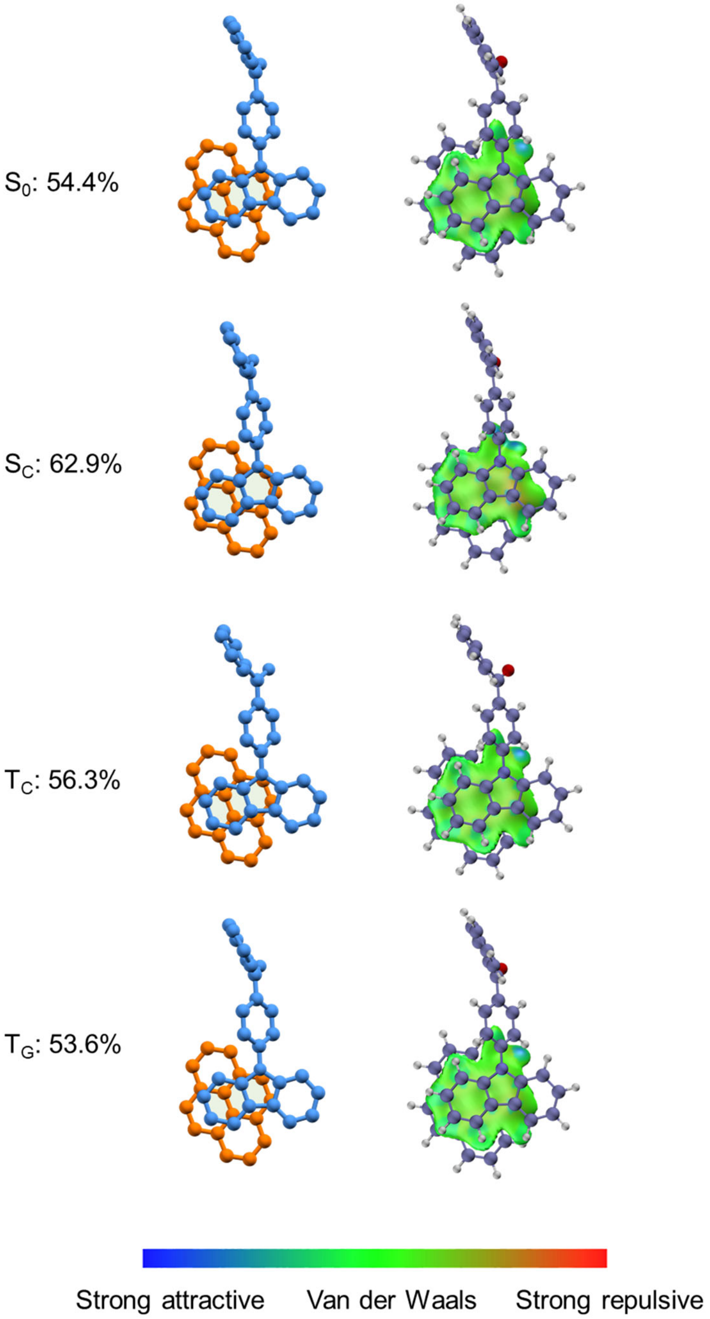

Supplementary Figure 83 | The optimized conformations of PY/CzBP dimer (left) and the distributions of NCI regions (right) in the different states. Color code in the conformations: blue, CzBP; red, PY. Hydrogen atoms were hidden for clear visualization. The overlap areas between aromatic ring planes were colored in light green. The overlap ratios were calculated by the proportion of the overlap areas to the areas of the carbazole moiety and labeled on the left side. Color code in the NCI analysis: ice blue, C; gray, H; red, O. The green areas represented the π–π interactions between the phenyl ring and carbazole moiety.

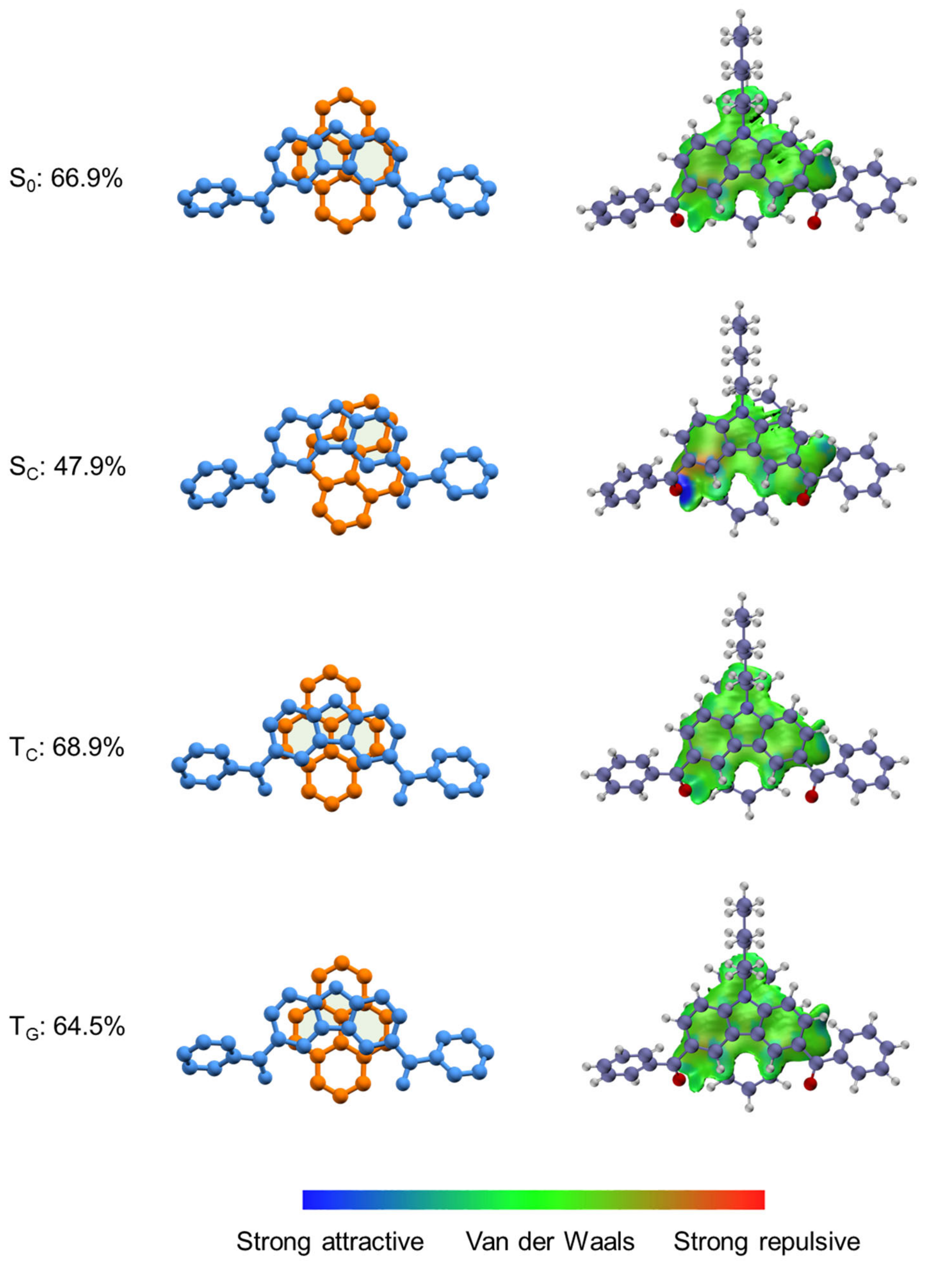

Supplementary Figure 84 | The optimized conformations of PY/BPCzA dimer (left) and the distributions of NCI regions (right) in the different states. Color code in the conformations: blue, BPCzA; red, PY. Hydrogen atoms and hexyl groups were hidden for clear visualization. The overlap areas between aromatic ring planes were colored in light green. The overlap ratios were calculated by the proportion of the overlap areas to the areas of the carbazole moiety and labeled on the left side. Color code in the NCI analysis: ice blue, C; gray, H; red, O. The green areas represented the π–π interactions between the phenyl ring and carbazole moiety. The blue areas represented the C–H···O interactions.

Enhanced ISC process through intramolecular D-A structure

The intramolecular donor-acceptor (D-A) structure in the PY−CzBP/BPCzA system combined benzophenone moiety and carbazole moiety together, generating more triplet states within the range of ±0.3 eV from S_1 state of complex to serve as ISC channels. For example, there were only intermolecular CT transitions (T_2, T_5) and locally-excited (LE) transitions located on CzA moiety (T_3, T_4) available for ISC process in PY−CzA system (Supplementary Fig. 85). But in PY−CzBP/BPCzA system, besides the transitions above, there were also intramolecular CT transitions (T_2 in PY−CzBP; T_2, T_7 in PY−BPCzA) and LE transitions located on BP moiety (T_4 in PY−CzBP; T_6 in PY−BPCzA), thus providing more ISC channels and accelerating ISC process (Supplementary Figs. 86 and 87).

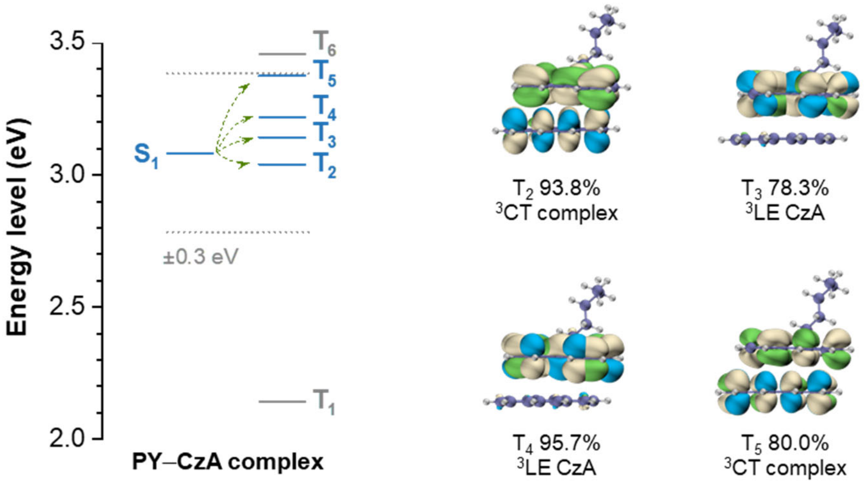

Supplementary Figure 85 | Calculated energy levels of PY−CzA complex and NTOs contributing to triplet states available for ISC process (energy levels located within the range of ±0.3 eV from S_1 state). Their spatial distributions, transition characteristics, and corresponding proportions were noted. Color code: green, hole; blue, particle.

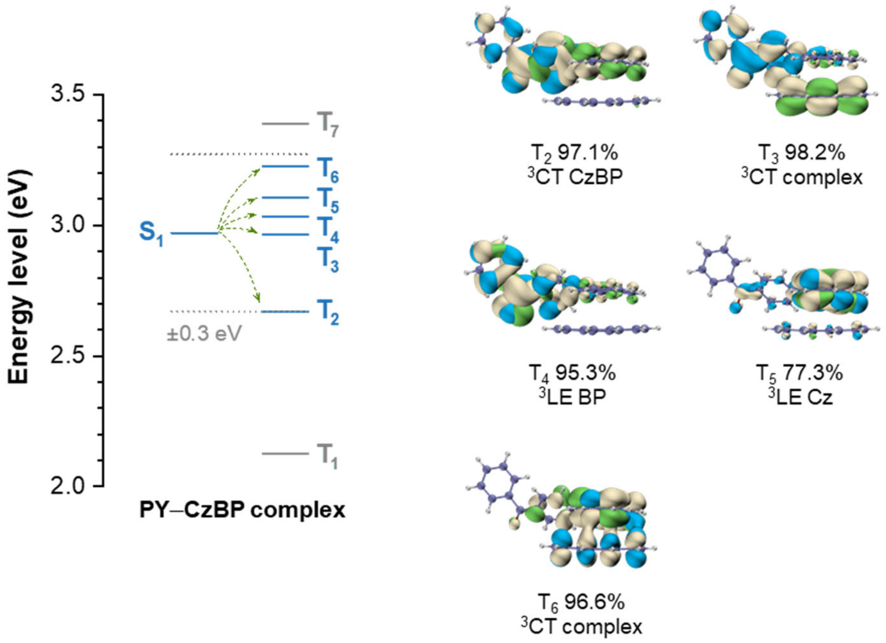

Supplementary Figure 86 | Calculated energy levels of PY–CzBP complex and NTOs contributing to triplet states available for ISC process (energy levels located within the range of ±0.3 eV from S_1 state). Their spatial distributions, transition characteristics, and corresponding proportions were noted. BP, benzophenone moiety; Cz, carbazole moiety. Color code: green, hole; blue, particle.

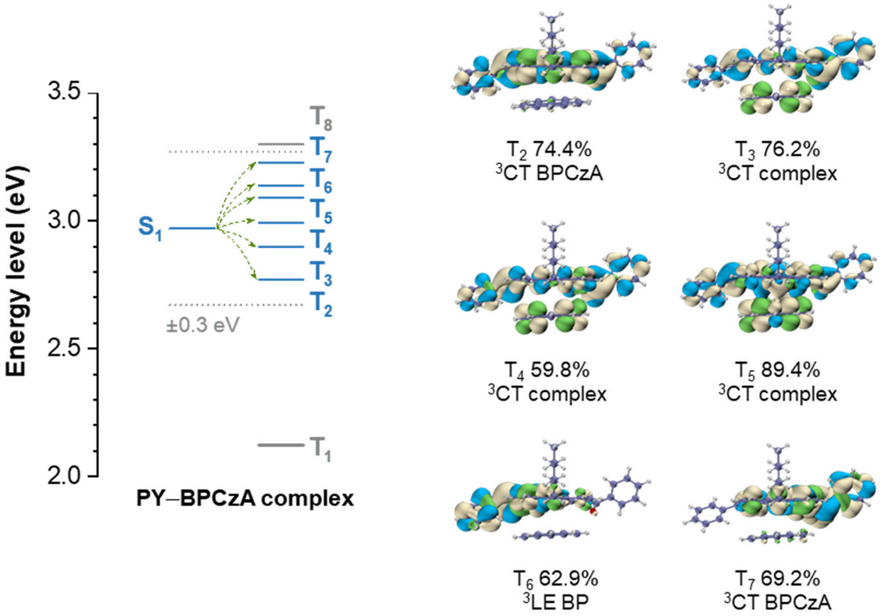

Supplementary Figure 87 | Calculated energy levels of PY−BPCzA complex and NTOs contributing to triplet states available for ISC process (energy levels located within the range of ±0.3 eV from S_1 state). Their spatial distributions, transition characteristics, and corresponding proportions were noted. BP, benzophenone moiety. Color code: green, hole; blue, particle.

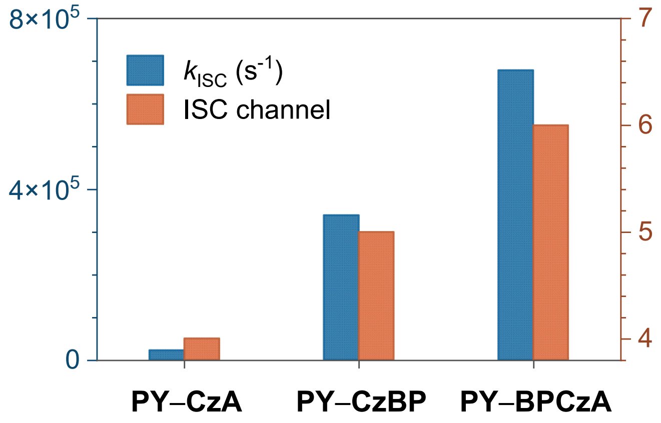

Supplementary Figure 88 | The relationship between the experimental rate constants of ISC process, k_{ISC}, and the calculated quantities of ISC channels. The values were also summarized in Supplementary Table 5.

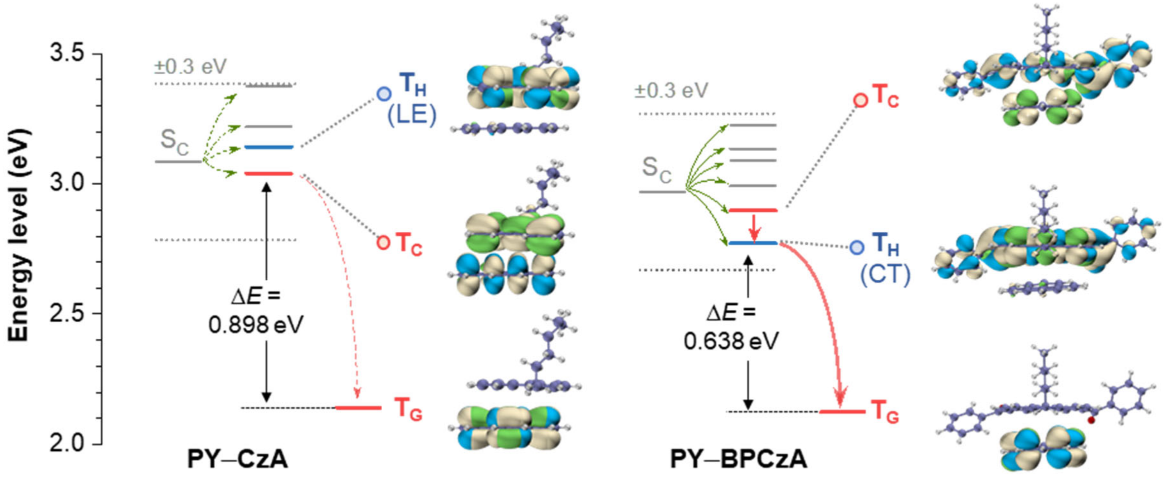

Supplementary Figure 89 | Calculated energy levels of PY−CzA and PY−BPCzA systems. Possible ISC channels were shown in green arrows and Δ*E* values for ET processes were noted. Insets: NTOs contributing to T_H, T_C, and T_G transitions in these systems. Color code: green, hole; blue, particle.

Similar to PY−CzBP system shown in Fig. 5f, the energy level of T_H in PY−BPCzA was also lowered by CT interaction of intramolecular D-A structure. Thus, it could work as an additional intermediate state to facilitate the decoupling process through lowering the energy gap.

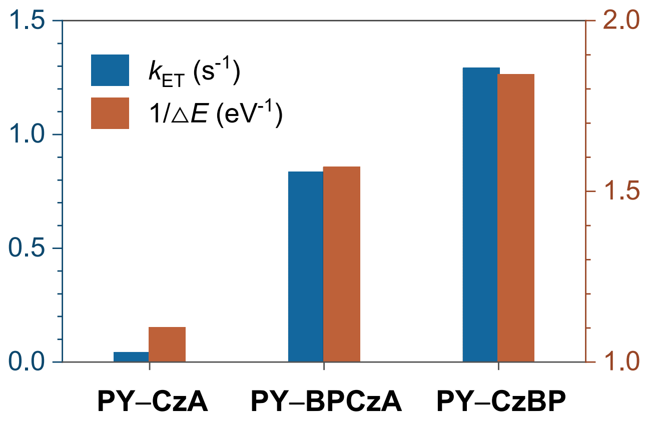

Supplementary Figure 90 | The relationship between the experimental rate constants of energy transfer process during decoupling, k_{ET}, and the reciprocal of the energy gap, $1/\Delta E$. ΔE represented the energy gap during decoupling process, corresponding to the energy difference between T_C and T_G for PY−CzA, and between T_H and T_G for PY−CzBP/BPCzA. The values were also summarized in Supplementary Table 6.

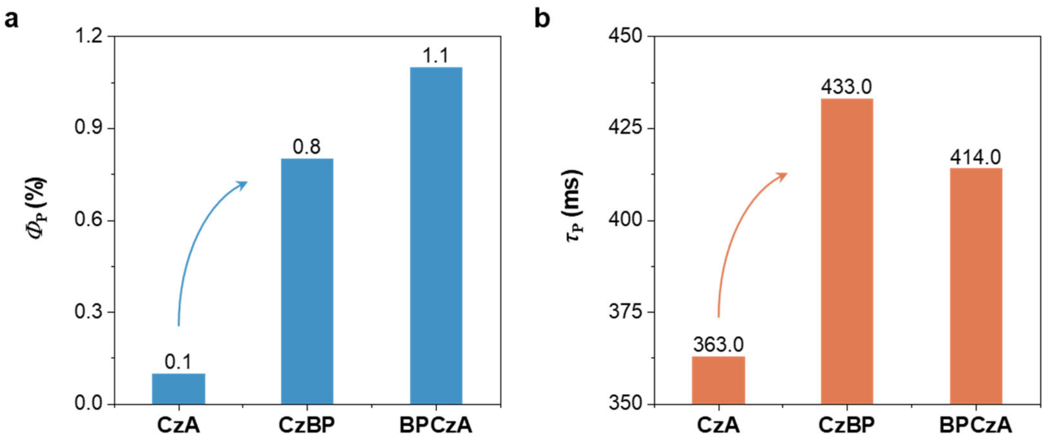

Supplementary Figure 91 | Phosphorescence quantum yields (a) and lifetime (b) of host−guest systems with PY as guest molecules.

Photophysical investigation on deuterated systems

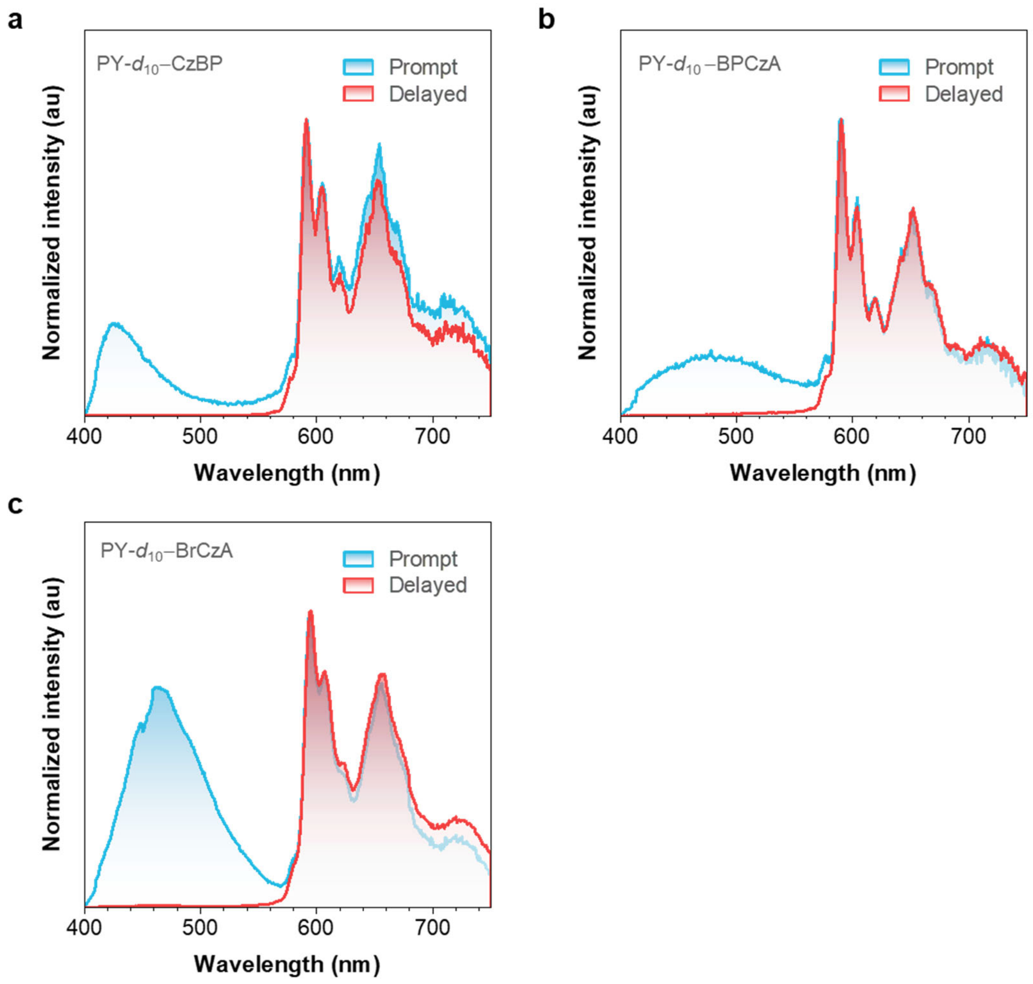

Supplementary Figure 92 | Prompt and delayed PL spectra of PY-d_{10}–CzBP (a), PY-d_{10}–BPCzA (b), and PY-d_{10}–BrCzA crystals excited at 365 nm. Delay time: 10 ms.

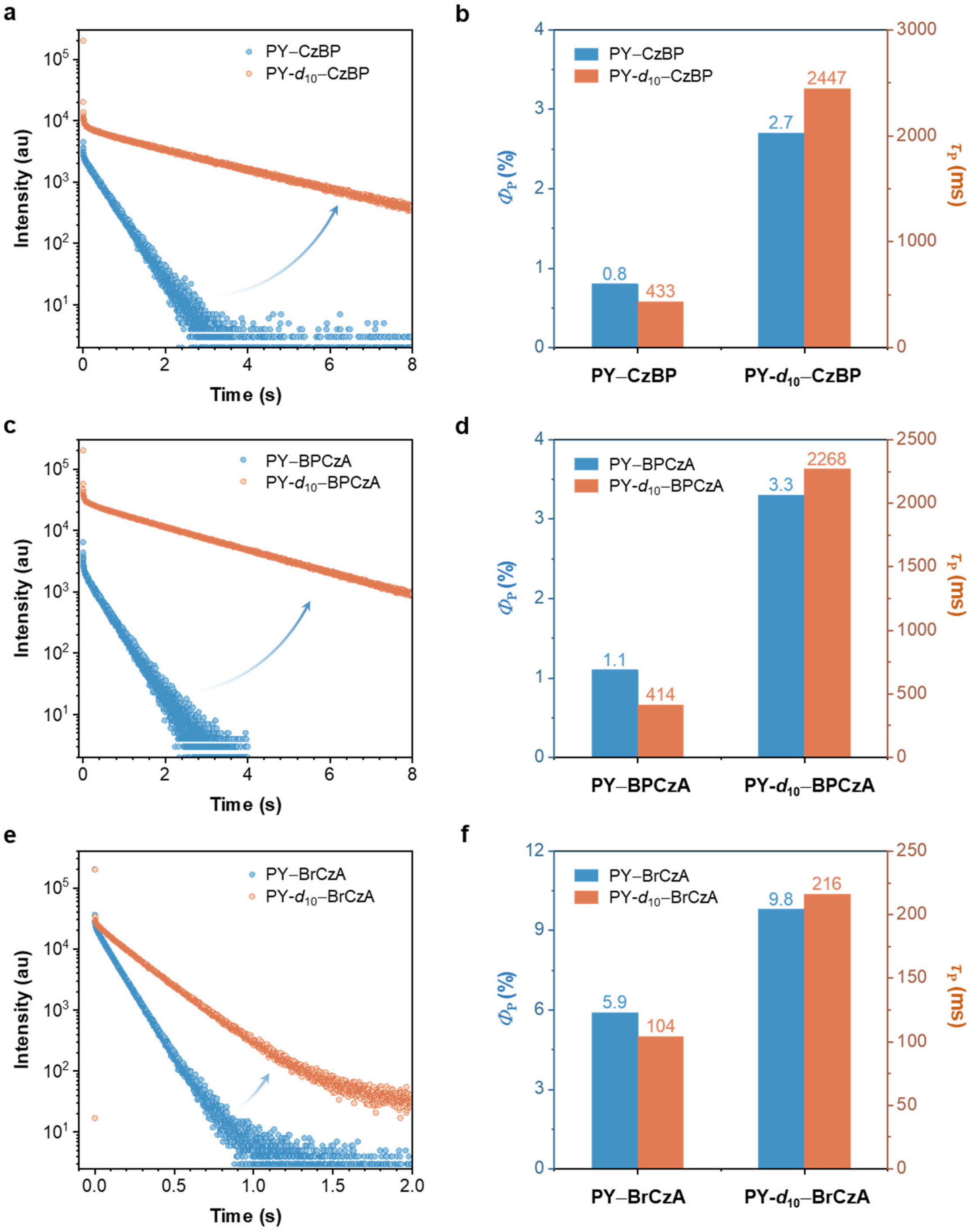

Supplementary Figure 93 |. Improvement of lifetime and quantum yields after deuteration of guest molecules. PY-d_{10}–CzBP (a,b), PY-d_{10}–BPCzA (c,d), and PY-d_{10}–BrCzA (e,f).

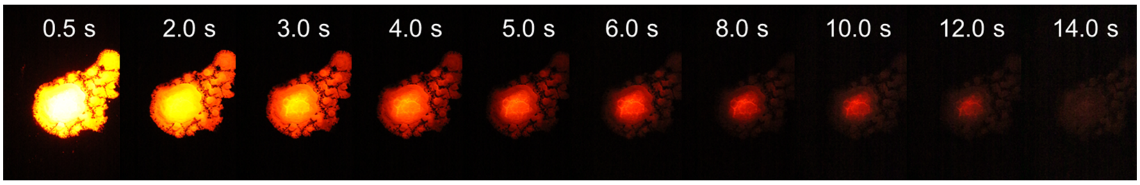

Supplementary Figure 94 | Photographs of PY-d_{10}–BPCzA crystals taken in air after 365 nm UV lamp was turned off.

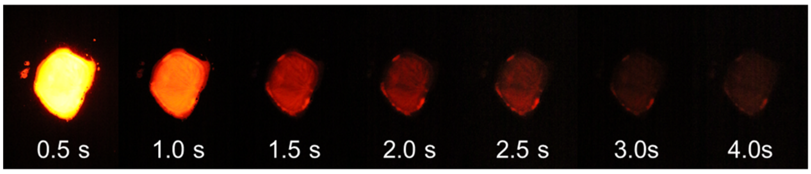

Supplementary Figure 95 | Photographs of PY-d_{10}–BrCzA crystals taken in air after 365 nm UV lamp was turned off.

VII. Quantitative analysis of dynamic coupling

As we discussed in Supplementary Section IV, the activation energy $\Delta G_{\mathrm{dc}}{}^{\neq}$ can be used as a semi-empirical parameter to quantify the dynamic coupling interaction and correlate it with RTP performance (Supplementary Fig. 96). According to Eq. S1, a higher activation energy corresponds to a slower electron exchange rate, thereby suppressing the decoupling process and weakening RTP emission from the $\mathrm{T_G}$ state, and *vice versa*.

$$k_{ex} = k_0 \exp\left(-\frac{\Delta G^{\neq}_{dc}}{RT}\right) \qquad \text{Eq. S1}$$

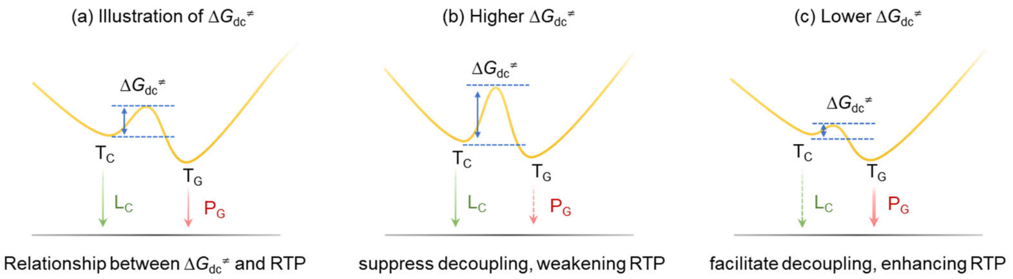

Supplementary Figure 96 | Illustration of the relationship between $\Delta G_{\mathrm{dc}}{}^{\neq}$ and RTP performance.

Below we will elaborate in detail on the application of this parameter to quantify dynamic coupling interactions in a series of host–guest systems investigated in our study. Specifically, we systematically analyzed the electronic effect in dynamic coupling process based on Marcus theory and compared $\Delta G_{\mathrm{dc}}{}^{\neq}$ across different host–guest systems.

Given that the decoupling process intrinsically involves electron exchange, the relative electron-donating and electron-accepting capabilities of the host and guest components will influence the activation energy barrier and ultimately govern the decoupling propensity of the coupling complex. According to Marcus theory[12], $\Delta G_{\mathrm{dc}}{}^{\neq}$ is associated with the thermodynamic free energy difference, $\Delta G_{\mathrm{dc}}{}^{0}$, and reorganization energy, λ, during decoupling process (Eq. S5).

$$\Delta G^{\neq}_{dc} = \frac{(\Delta G^{0}_{dc} + \lambda)^2}{4\lambda} \qquad \text{Eq. S5}$$

$\Delta G_{\mathrm{dc}}{}^{0}$, representing the energy difference between coupling complex and decoupled host/guest pairs after electron exchange, can be estimated by measuring the electrochemical potentials for the oxidations $E^{0}_{(D^{\cdot+}/D)}$ and reductions $E^{0}_{(A/A^{\cdot-})}$ via cyclic voltammetry experiments[12], as per Eq. S6. The organizational energy (λ) of the system, representing internal molecular reorganization from $\mathrm{T_C}$ state to $\mathrm{T_G}$ state, since external solvent reorganization is negligible in the crystal state, can be estimated using DFT[13].

$$\Delta G^{0}_{dc} \approx \mathcal{F}E^{0}_{(D^{\cdot+}/D)} - \mathcal{F}E^{0}_{(A/A^{\cdot-})} - E_{D^*/A^*} \qquad \text{Eq. S6}$$

E_{D^*/A^*} denotes the excitation energy of either the donor or acceptor, depending on which molecular entity is actually excited in the photophysical process.

Based on Eqs. S5–S6, we can determine the semi-empirical physical parameter $\Delta G_{\mathrm{dc}}{}^{\neq}$ for each system by calculating the corresponding $\Delta G_{\mathrm{dc}}{}^{0}$ and λ values. Thus, we select four representative systems in our work with PY as guest and carbazole derivatives as hosts (PY–CzA, PY–BrCzA, PY–CzBP, and PY–BPCzA). To strengthen our dataset, we incorporate additional PY–BP system where benzophenone (BP) serves as the host. This is because BP represents the fundamental structural unit of CzBP/BPCzA, and PY–BP has been widely reported as an efficient RTP system[14-

[16]. Their ΔG_{dc}^0 , λ and $\Delta G_{dc}^{\neq}$ values are listed in Supplementary Table 7. Cyclic voltammetry curves used for determining the electrochemical potentials are provided in Supplementary Fig. 97 and Supplementary Table 8.

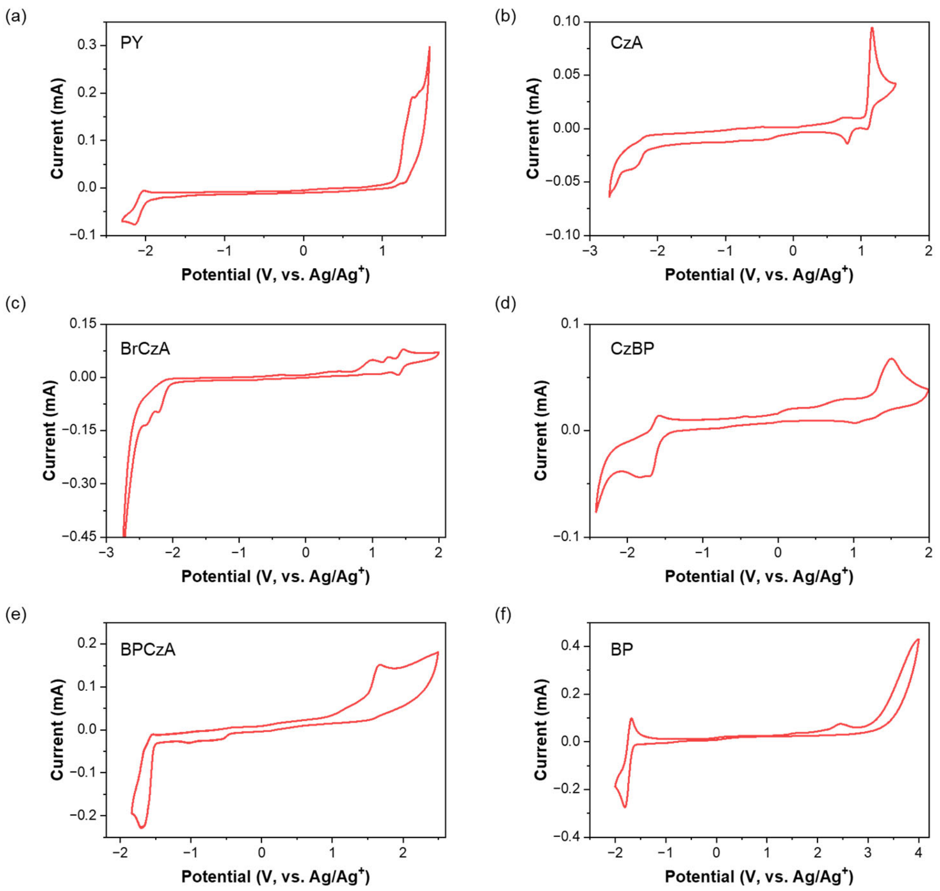

Supplementary Figure 97 | Cyclic voltammetry curves of PY and host molecules in MeCN.

Correlation of $\Delta G_{dc}^{\neq}$ with RTP performance

As established in Section IV, the rate constant of electron exchange during decoupling process (k_{ex}) directly controls the population of excitons reaching T_G (Φ_T). For systems exhibiting comparable triplet decay rates ($k_P + k_{nr,P}$), the observed phosphorescence lifetime (τ_P) demonstrates a linear dependence on both Φ_T, and consequently, on k_{ex}.

$$\tau_P \propto \phi_T \propto k_{ex} \qquad \text{Eq. S7}$$

Therefore, we selected τ_P as the key performance metric for RTP, while $\Delta G_{dc}^{\neq}$ serves as our semi-empirical parameter to quantify the decoupling tendency in the dynamic coupling process.

Based on Eqs. S1 and S7, we can derive an exponential relationship between τ_P and $\Delta G_{dc}^{\neq}$:

$$\tau_P \propto \exp\left(-\Delta G_{dc}^{\neq}\right)$$

Consequently, a negative linear correlation exists between $\ln(\tau_P)$ and $\Delta G_{dc}^{\neq}$:

$$\ln(\tau_P) \propto -\Delta G_{dc}^{\neq}$$

Supplementary Fig. 98 reveals an excellent linear correlation (***R^2* = 0.99**) between $\ln(\tau_P)$ and $\Delta G_{dc}^{\neq}$, which strongly supports the proposed mechanism of dynamic coupling-induced RTP. The observed negative slope reveals a systematic trend across the series from PY–CzBP to PY–BPCzA to PY–CzA to PY–BP: as the activation energy

increases, the decoupling efficiency progressively decreases, resulting in corresponding reduction of RTP lifetimes, which is well consistent with our discussion in Supplementary Fig. 96. For the PY–BrCzA system, the heavy-atom effect (HAE) significantly increases the triplet decay rates ($k_P + k_{nr, P}$), which makes the proportional relationship between τ_P and k_{ex} in Eq. S7 invalid. Thus, this deviation can be excluded reasonably from the linear fitting analysis (Supplementary Fig. 99). Such an excellent linear correlation observed in Supplementary Fig. 98 strongly suggests that the phosphorescence performance in different host–guest systems can be quantitatively predicted by calculating the activation energy $\Delta G_{dc}^{\neq}$.

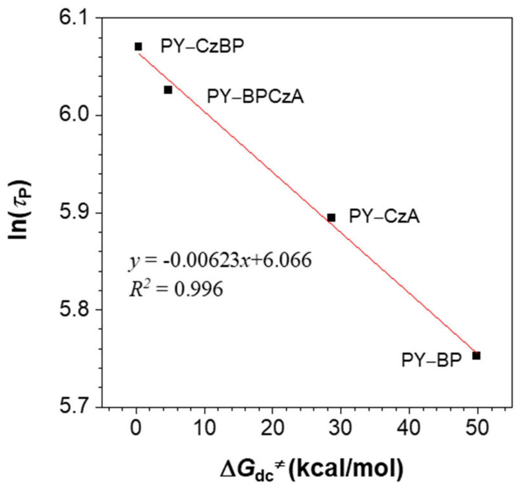

Supplementary Figure 98 | Relationship between phosphorescence lifetime τ_P and the activation energy for decoupling process, $\Delta G_{dc}^{\neq}$, for various host–guest systems.

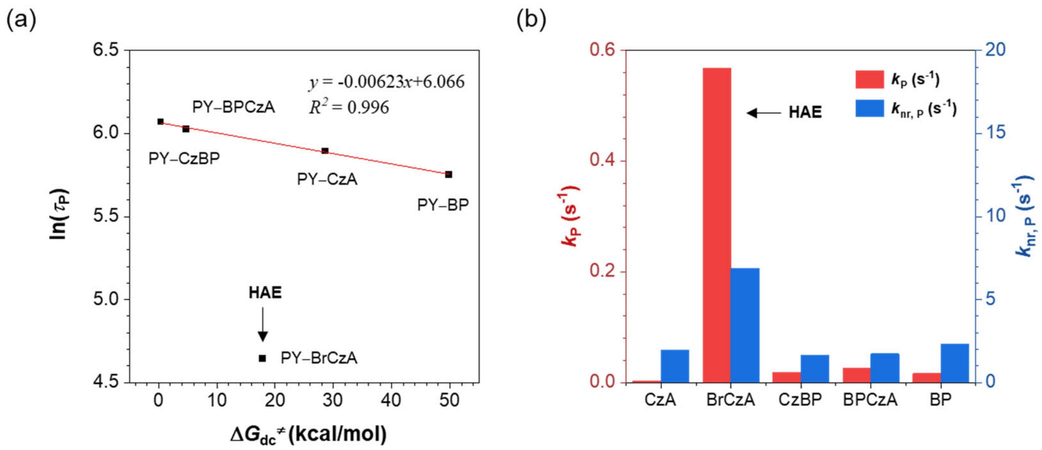

Supplementary Figure 99 | (a) Deviation of PY–BrCzA system from fitted linear relationship due to heavy atom effect. (b) Experimental rate constants of radiative decay, k_P, and nonradiative decay, $k_{nr, P}$, from T_G state in these systems.

VIII. Applications

Demonstration of multicolor patterning

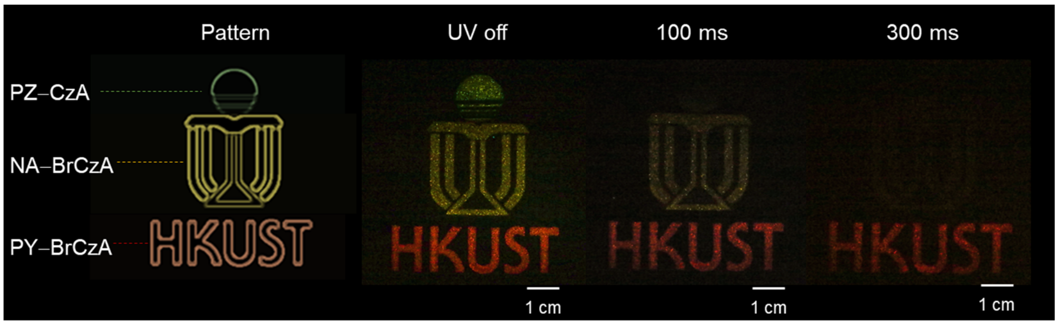

Supplementary Figure 100 | Photographs of HKUST logo taken after the 365 nm lamp was turned off. The pattern was fabricated through screen printing with PZ–CzA, NA–BrCzA, and PY–BrCzA crystals (left) as ink, respectively.

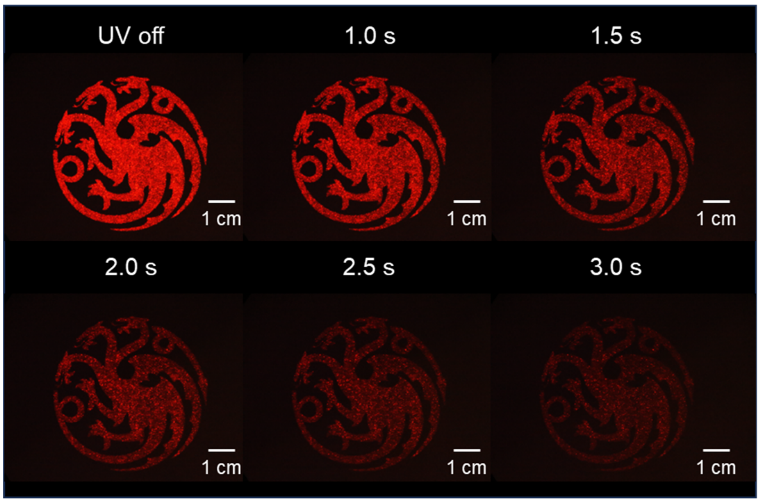

Supplementary Figure 101 | Photographs of Targaryen dragon symbol taken after the 365 nm lamp was turned off. The pattern was fabricated through screen printing with PY–BPCzA crystals as ink.

Photophysical properties of host–guest RTP systems used in 4D encryption

The design concept of 4D encryption involved arranging samples with different RTP emission into a 2D pattern (Fig. 6a), and subsequently incorporating the dimensions of time and letter encryption. Thus, 8 samples exhibiting red, yellow and green RTP emissions were chosen (Supplementary Fig. 102).

Their PL spectra could be found in Supplementary Figs. 60, 62, 66, 92, 103 and 104. The lifetime curves of samples displaying red, yellow and green RTP were shown in Supplementary Figs. 105–107, respectively. Their quantum yields were summarized in Supplementary Table 4.

In the temporal dimension, the emission from samples with shorter lifetime gradually diminished over time, resulting in a time-dependent pattern composed of various color combinations in Fig. 6c.

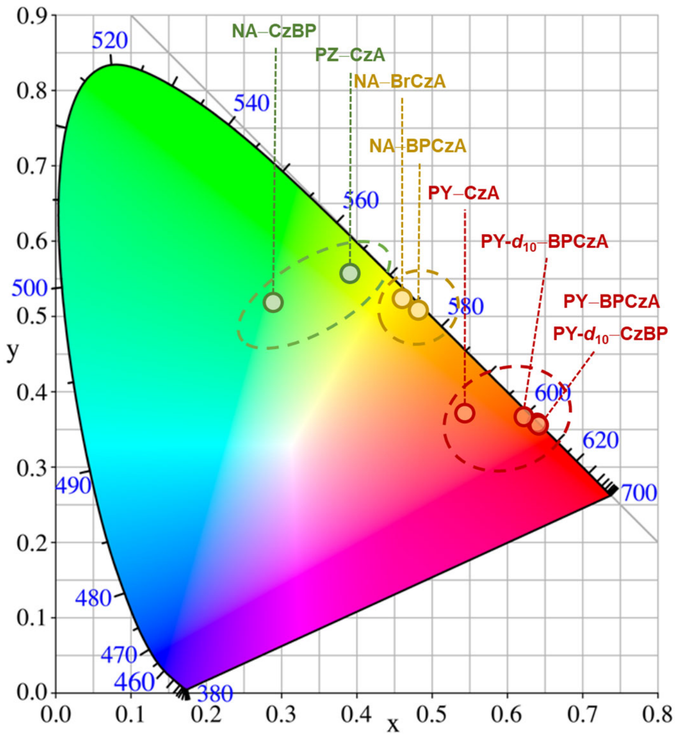

Supplementary Figure 102 | CIE coordinate diagram of 8 host–guest RTP samples used in 4D encryption application. CIE coordinates: PY–CzA, (0.54, 0.37); PY–BPCzA, (0.64, 0.36); PY-d_{10}–CzBP, (0.64, 0.36); PY-d_{10}–BPCzA, (0.62, 0.37); NA–BrCzA, (0.46, 0.52); NA–BPCzA, (0.48, 0.51); NA–CzBP, (0.29, 0.49); PZ–CzA, (0.39, 0.56).

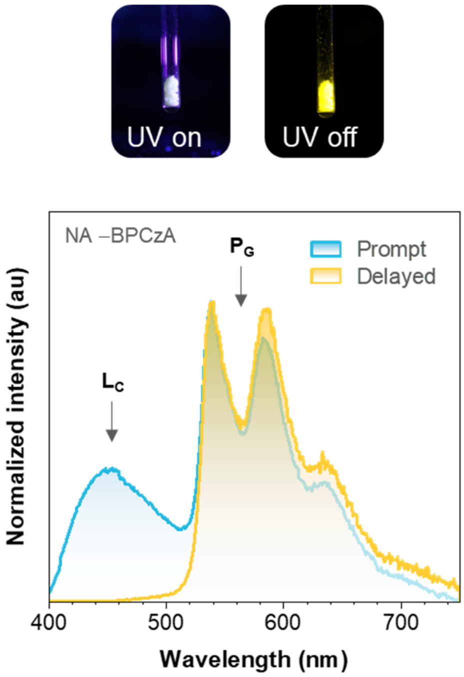

Supplementary Figure 103 | Prompt and delayed PL spectra of NA–BPCzA crystals excited at 365 nm. Delay time: 10 ms. The emission of complex L_C, and guest phosphorescence, P_G, were noted. The inset showed photographs of NA–BPCzA crystals taken with the 365 nm UV lamp turned on and off.

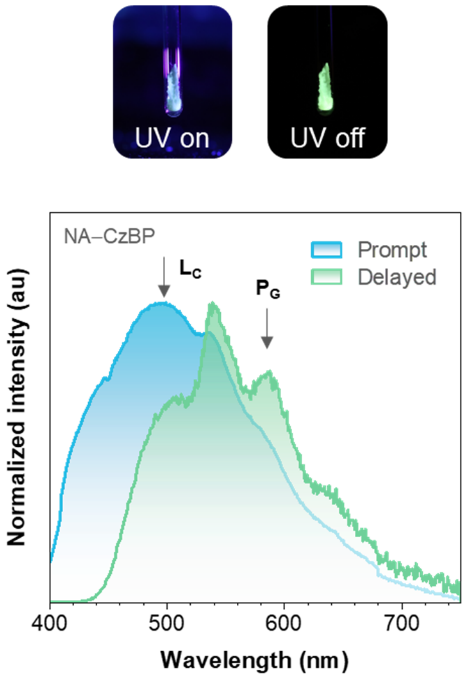

Supplementary Figure 104 | Prompt and delayed PL spectra of NA–CzBP crystals excited at 365 nm. Delay time: 10 ms. The emission of complex L_C, and guest phosphorescence, P_G, were noted. The inset showed photographs of NA–CzBP crystals taken with the 365 nm UV lamp turned on and off.

The green RTP of NA–CzBP system was a mixture of guest phosphorescence and complex emission, further suggesting the enormous potential of dynamic coupling-induced RTP in displaying a more diverse range of phosphorescent colors.

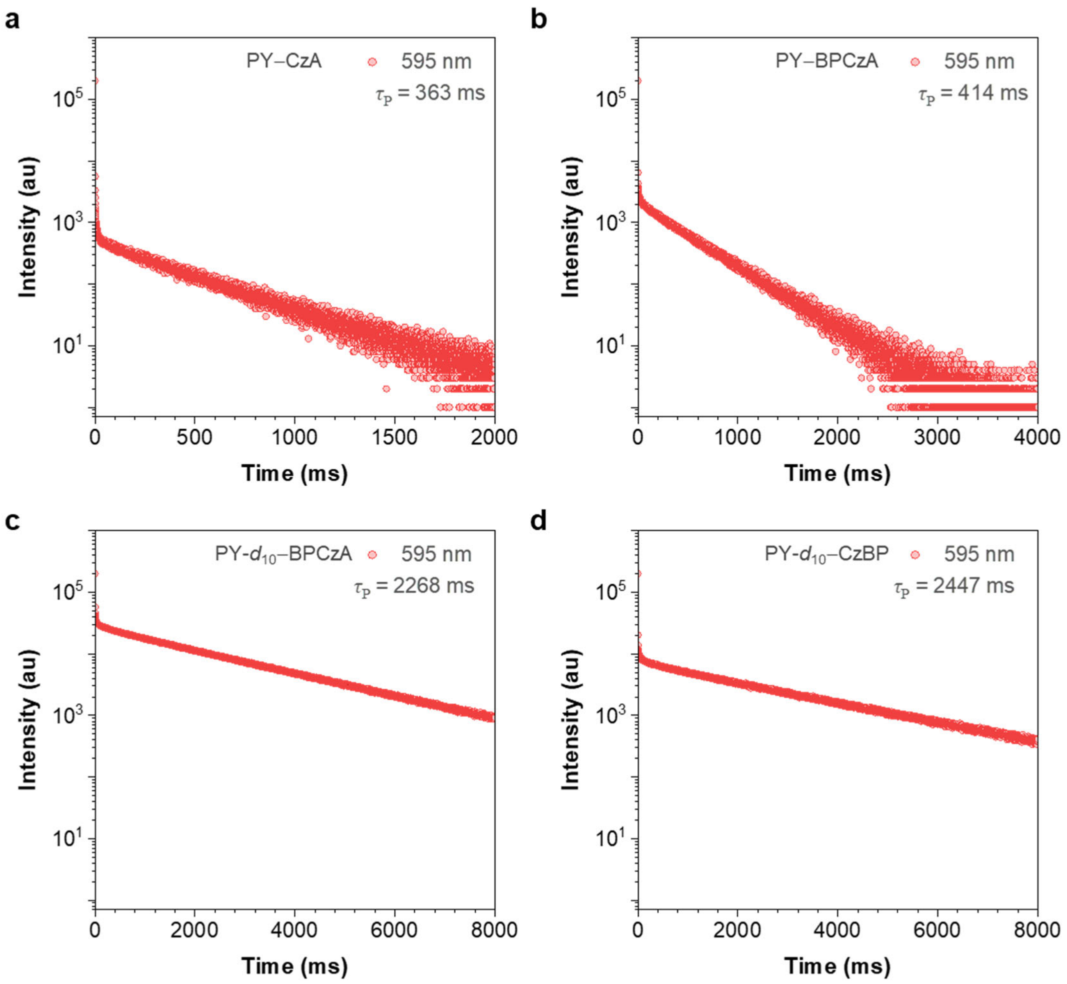

Supplementary Figure 105 | Lifetime curves of red RTP samples excited at 365 nm, including PY−CzA (a), PY−BPCzA (b), PY-d_{10}−BPCzA (c), and PY-d_{10}−CzBP (d). The fitted lifetime values of phosphorescence were indicated.

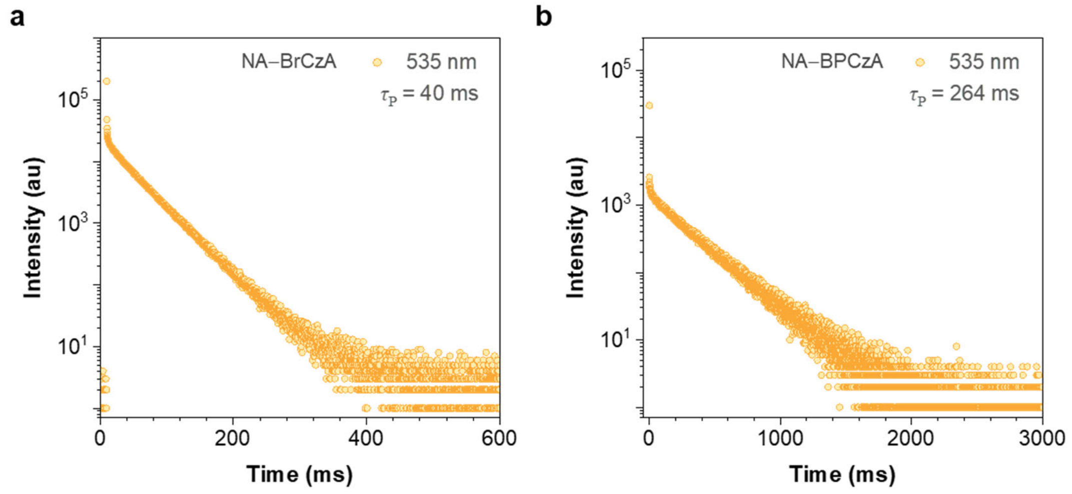

Supplementary Figure 106 | Lifetime curves of yellow RTP samples excited at 365 nm, including NA–BrCzA (a), and NA–BPCzA (b). The fitted lifetime values of phosphorescence were indicated.

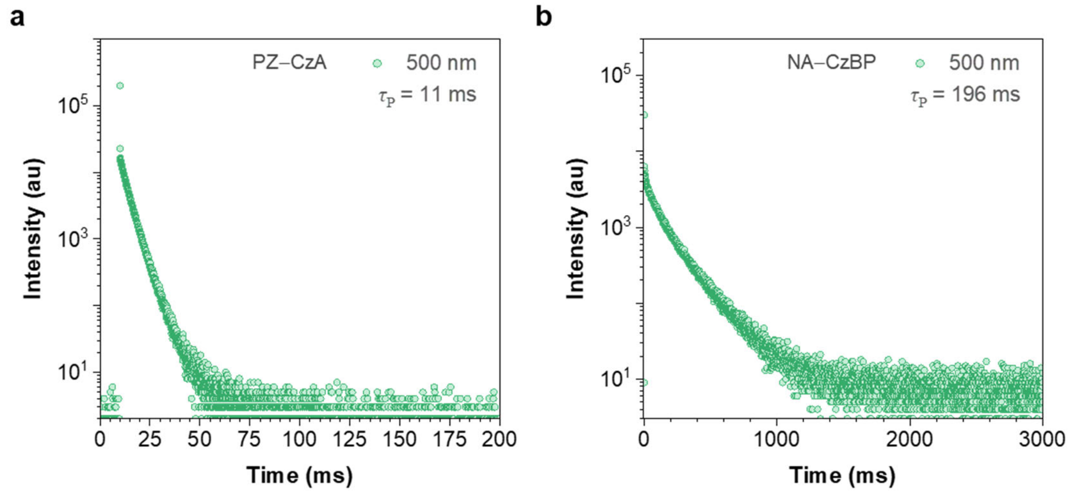

Supplementary Figure 107 | Lifetime curves of green RTP samples excited at 365 nm, including PZ–CzA (a), NA–CzBP (b). The fitted lifetime values were indicated.

Temperature-dependent full-color phosphorescent display

In a typical two-component system with a single TTET pathway between two phosphorescent emitters, T_H and T_G, the alternation of afterglow color showed a linear relationship after converting the delayed PL spectra to CIE chromaticity coordinates (Supplementary Fig. 108a,c), making it difficult to realize full-color phosphorescent display within one sample[17,18].

However, the existence of multiple phosphorescent emitters (T_H, T_G, T_C) in dynamic coupling-induced RTP systems allowed a nonlinear changes of afterglow color. By simply controlling temperature, the component of multiple phosphorescence (P_H, P_G, P_C) in the delayed emission could be carefully adjusted to realize a wide range of afterglow colors (Supplementary Fig. 108b,d).

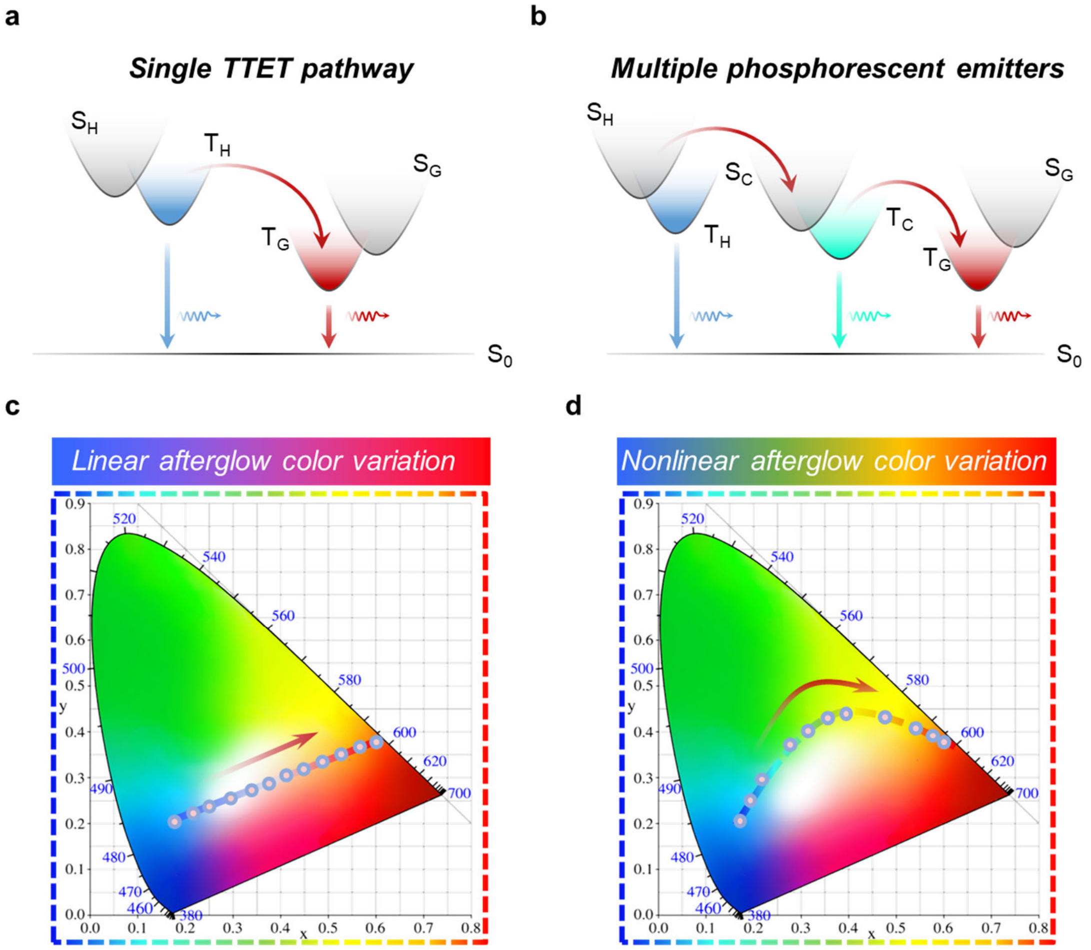

Supplementary Figure 108 | Photophysical processes and CIE coordinate diagrams in typical two-component systems (a,c) and systems involved dynamic coupling (b,d). The red curved arrows in a and b represented the ET processes could be enhanced as temperature increased.

Taking PY−BPCzA as an example (Fig. 6d), the delayed PL spectrum of PY−BPCzA at 60 K matched well with the phosphorescence spectrum of BPCzA crystals (Supplementary Fig. 32), because both non-radiative decay from triplet state of host molecules and the intermolecular ET process were suppressed at low temperature. After heating to 90 K, P_H seemed to disappear and the delayed PL spectra resembled the complex emission, indicating the formation of complex. As temperature kept increasing, P_C continued to decrease with P_G gradually emerged, suggesting the ET process from T_C to T_G was activated, which was also supported by the thermal activation characteristic of lifetime curves recorded at P_G in Supplementary Fig. 109.

The major component in the delayed spectra gradually transitioned from P_H to P_C and then to P_G. The various combinations of them at different temperature realized a full-color phosphorescent display as shown in Fig. 6f.

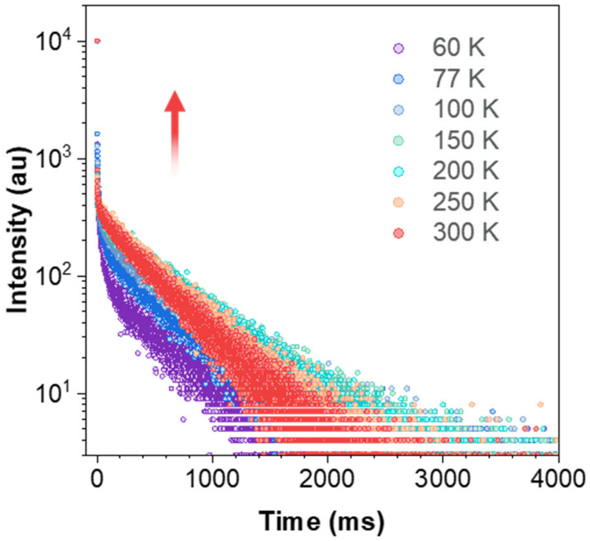

Supplementary Figure 109 | Lifetime curves of P_G in PY−BPCzA crystals at different temperature, recorded at 595 nm. The component ratio of P_G kept increasing as the temperature elevated, supporting the similar ET process from T_C to T_G as in PY−CzA crystals.

IX. Supplementary Tables

Table S1. Crystallographic data for the single crystal of BrCzA.

Single crystal	BrCzA
Empirical formula	$C_{18}H_{19}Br_2N$
Formula weight	409.16
Temperature/K	100.02(10)
Crystal system	orthorhombic
Space group	$Pca2_1$
a/Å	20.1618(4)
b/Å	4.48671(8)
c/Å	18.2464(4)
α/°	90
β/°	90
γ/°	90
Volume/Å^3	1650.57(6)
Z	4
ρ_{calc}/g·cm^{-3}	1.647
μ/mm^{-1}	6.156
F(000)	816.0
Crystal size/mm^3	0.16 × 0.12 × 0.07
Radiation	CuKα (λ = 1.54184)
2θ range for data collection/°	8.772 to 153.992
Index ranges	$-25 \le h \le 24$, $-5 \le k \le 4$, $-20 \le l \le 23$
Reflections collected	9481
Independent reflections	3134 [R_{int} = 0.0379, R_{sigma} = 0.0370]
Data/restraints/parameters	3134/1/191
Goodness-of-fit on F^2	1.048
Final R indexes [I>=2σ (I)]	R_1 [a] = 0.0340, wR_2 [b] = 0.0841
Final R indexes [all data]	R_1 = 0.0380, wR_2 = 0.0878
Largest diff. peak/hole / e Å^{-3}	0.93/-0.62
Flack parameter	-0.08(3)

[a] $R_1=\Sigma||F_o|-|F_c||/\Sigma|F_o|$.

[b] $wR_2=[\Sigma w(F_o^2-F_c^2)^2/\Sigma w(F_o^2)^2]^{1/2}$.

Table S2. Crystallographic data for the single crystal of CzBP.

Single crystal	CzBP
Empirical formula	$C_{25}H_{17}NO$
Formula weight	347.39
Temperature/K	100.01(10)
Crystal system	monoclinic
Space group	$P2_1/c$
a/Å	9.47643(18)
b/Å	8.96758(19)
c/Å	20.3923(4)
α/°	90
β/°	98.3815(18)
γ/°	90
Volume/Å^3	1714.44(6)
Z	4
ρ_{calc}/g·cm^{-3}	1.346
μ/mm^{-1}	0.638
F(000)	728.0
Crystal size/mm^3	0.2 × 0.18 × 0.15
Radiation	CuKα (λ = 1.54184)
2θ range for data collection/°	8.766 to 154.24
Index ranges	$-11 \leq h \leq 9$, $-10 \leq k \leq 11$, $-25 \leq l \leq 22$
Reflections collected	6219
Independent reflections	3496 [R_{int} = 0.0190, R_{sigma} = 0.0265]
Data/restraints/parameters	3496/0/245
Goodness-of-fit on F^2	1.037
Final R indexes [I>=2σ (I)]	R_1 [a] = 0.0354, wR_2 [b] = 0.0881
Final R indexes [all data]	R_1 = 0.0419, wR_2 = 0.0930
Largest diff. peak/hole / e Å^{-3}	0.26/-0.21

[a] $R_1=\Sigma||F_o|-|F_c||/\Sigma|F_o|$.

[b] $wR_2=[\Sigma w(F_o^2-F_c^2)^2/\Sigma w(F_o^2)^2]^{1/2}$.

Table S3. Crystallographic data for the single crystal of BPCzA.

**Single crystal**	**BPCzA**
Empirical formula	$C_{32}H_{29}NO_2$
Formula weight	459.56
Temperature/K	100.01(10)
Crystal system	trigonal
Space group	$P3_221$
a/Å	10.49260(10)
b/Å	10.49260(10)
c/Å	19.0594(2)
α/°	90
β/°	90
γ/°	120
Volume/Å^3	1817.21(4)
Z	3
ρ_{calc}/g·cm^{-3}	1.260
μ/mm^{-1}	0.607
F(000)	732.0
Crystal size/mm^3	0.25 × 0.12 × 0.1
Radiation	CuKα (λ = 1.54184)
2θ range for data collection/°	9.734 to 154.046
Index ranges	$-13 \leq h \leq 12$, $-13 \leq k \leq 12$, $-18 \leq l \leq 24$
Reflections collected	12358
Independent reflections	2544 [R_{int} = 0.0175, R_{sigma} = 0.0126]
Data/restraints/parameters	2544/57/191
Goodness-of-fit on F^2	1.084
Final R indexes [I>=2σ (I)]	$R_1{}^a$ = 0.0327, w$R_2{}^b$ = 0.0827
Final R indexes [all data]	R_1 = 0.0331, wR_2 = 0.0830
Largest diff. peak/hole / e Å^{-3}	0.15/-0.18
Flack parameter	0.01(8)

[a] $R_1=\Sigma||F_o|-|F_c||/\Sigma|F_o|$.

[b] $wR_2=[\Sigma w(F_o^2-F_c^2)^2/\Sigma w(F_o^2)^2]^{1/2}$.

Supplementary Table 4 | RTP performance of host–guest systems in this work.

Sample	Φ_{total} (%)	Φ_P (%) [a]	τ_P (ms)
PY–CzA	28.3	0.1	363
PY–BrCzA	8.2	5.9	104
PY–CzBP	1.2	0.8	433
PY–BPCzA	1.9	1.1	414
PY-d_{10}–BrCzA	10.9	9.8	216
PY-d_{10}–CzBP	3.2	2.7	2447
PY-d_{10}–BPCzA	3.9	3.3	2268
NA–BrCzA	14.9	14.1	40
NA–CzBP	3.8	3.3	196
NA–BPCzA	3.5	2.9	264
PZ–CzA	3.2	1.8	11

[a] Φ_P was calculated by the phosphorescence component in the photoluminescence spectra. If phosphorescence was too weak to be distinguished from fluorescence in the photoluminescence spectra, Φ_P was determined based on phosphorescence component associated with relevant lifetimes.

Supplementary Table 5 | Photophysical properties of PY–BrCzA under different pressure.

External pressure (GPa)	τ (ns) [a]	k_r (× 10^5 s^{-1})	k_{nr} (× 10^7 s^{-1})
0.0	32.55	7.07	2.82
0.4	37.19	3.93	2.55
1.1	29.85	2.76	3.25
1.8	24.53	2.66	3.98
4.1	19.01	2.37	5.18
6.1	12.79	1.94	7.75
10.0	3.26	1.92	30.60

[a] Lifetime was measured at 500 nm.

Supplementary Table 6 | Photophysical properties of complex in different host–guest systems.

Sample	$\tau_{F,C}$ (ns) [a]	k_{ISC} ($\times 10^4$ s^{-1}) [b]	ISC channels	$\tau_{P,C}$ (ms) [a]	k_{ET} (s^{-1}) [b]	ΔE (eV) [c]
PY−CzA	44.1	2.3	4	25.2	0.04	0.898
PY−CzBP	23.6	33.9	5	6.2	1.29	0.543
PY−BPCzA	16.2	67.9	6	13.2	0.83	0.638

[a] $\tau_{F,C}$, $\tau_{P,C}$ were lifetime of complex in the singlet state and triplet state, respectively. They were fitted by lifetime curves measured with emission recorded at L_C in the nanosecond range and millisecond range, respectively.

[b] k_{ISC} is rate constant of ISC process and k_{ET} is rate constant of ET process from T_C to T_G state. Their values were determined according to equation: $k_{ISC} = \Phi_P / \tau_{F,C}$; $k_{ET} = \Phi_P / \tau_{P,C}$. The values of Φ_P can be found in Supplementary Table 4.

[c] ΔE represented the energy gap during decoupling process, corresponding to the energy difference between T_C and T_G for PY−CzA, and between T_H and T_G for PY−CzBP/BPCzA.

Supplementary Table 7 | Thermodynamic parameters of host–guest systems during dynamic coupling process.

	PY−CzA	PY−BrCzA	PY−CzBP	PY−BPCzA	PY−BP
ΔG_{dc}^0 (kcal/mol)	-36.50	-38.28	-20.24	-34.55	-50.73
λ (kcal/mol)	7.40	10.67	15.27	16.81	8.81
$\Delta G_{dc}^{\neq}$ (kcal/mol)	28.60	17.85	0.40	4.69	49.88
τ_P (ms)	363	104	433	414	315

Supplementary Table 8 | The electrochemical potential for the oxidation and reduction of PY and host molecules.

	PY	CzA	BrCzA	CzBP	BPCzA	BP
$E^0_{(M^{\cdot +}/M)}$ (V)	1.20	1.10	0.80	1.27	1.49	2.10
$E^0_{(M/M^{\cdot -})}$ (V)	-1.93	-2.20	-2.07	-1.55	-1.50	-1.65

X. References

(1) Frisch, M. J. et al. Gaussian 16, Gaussian, Inc., Wallingford CT, 2016.

(2) Neese, F. The ORCA program system. *Wiley Interdiscip. Rev.: Comput. Mol. Sci.* **2**, 73−78 (2012).

(3) Lu, T. & Chen, F. Multiwfn: a multifunctional wavefunction analyzer. *J. Comput. Chem.* **33**, 580−592 (2012).

(4) Lefebvre, C. et al. Accurately extracting the signature of intermolecular interactions present in the NCI plot of the reduced density gradient versus electron density. *Phys. Chem. Chem. Phys.* **19**, 17928−17936 (2017).

(5) Macrae, C. F. et al. Mercury 4.0: from visualization to analysis, design and prediction. *J. Appl. Cryst.* **53**, 226–235 (2020).

(6) Humphrey, W., Dalke, A. & Schulten, K. VMD: visual molecular dynamics. *J. Mol. Graph.* **14**, 33−38 (1996).

(7) Dennington, R., Keith, T. A. & Millam, J. M. GaussView, Version 6, Semichem Inc., Shawnee Mission, KS, 2016.

(8) Schneider, C. A., Rasband, W. S. & Eliceiri, K. W. NIH Image to ImageJ: 25 years of image analysis. *Nat. Methods* **9**, 671−675 (2012).

(9) Li, W. et al. Alkyl chain introduction: in situ solar-renewable colorful organic mechanoluminescence materials. *Angew. Chem. Int. Ed.* **57**, 12727−12732 (2018).

(10) Nidhankar, A. D. et al. Self-assembled helical arrays for the stabilization of the triplet state. *Angew. Chem. Int. Ed.* **59**, 13079−13085 (2020).

(11) Li, J.-A. et al. Colour-tunable dual-mode afterglows and helical-array-induced mechanoluminescence from AIE enantiomers: effects of molecular arrangement on formation and decay of excited states. *Chem. Eng. J.* **418**, 129167 (2021).

(12) Turro, N. J., Ramamurthy, V. & Scaiano, J. C. *Modern Molecular Photochemistry of Organic Molecules.* (University Science Books, New York, 2010).

(13) Ma, L. et al. Triplet exciplex mediated multi-color ultra-long afterglow materials. *Angew. Chem. Int. Ed.* **64**, e202500847 (2025).

(14) Xiao, F. et al. Guest-host doped strategy for constructing ultralong-lifetime near-infrared organic phosphorescence materials for bioimaging. *Nat. Commun.* **13**, 186 (2022).

(15) Qiu, W. et al. Achieving purely organic room-temperature phosphorescence mediated by a host–guest charge transfer state. *J. Phys. Chem. Lett.* **12**, 4600–4608 (2021).

(16) Yang, G. et al. Construction and application of large stokes-shift organic room temperature phosphorescence materials by intermolecular charge transfer. *J. Phys. Chem. Lett.* **14**, 6927–6934 (2023).

(17) Liang, Y. et al. Color-tunable dual-mode organic afterglow from classical aggregation-caused quenching compounds for white-light-manipulated anti-counterfeiting. *Angew. Chem. Int. Ed.* **62**, e202217616 (2023).

(18) Yang, Y. et al. Efficient and color-tunable dual-mode afterglow from large-area and flexible polymer-based transparent films for anti-counterfeiting and information encryption. *Angew. Chem. Int. Ed.* **61**, e202201820 (2022).
